# Nucleophile-triggered prodrug release from polymer hydrogels

**DOI:** 10.1039/d5lp00317b

**Published:** 2025-11-10

**Authors:** Benjamin Klemm, Magherita Tavasso, Irene Piergentili, Max Satijn, Tobias G. Brevé, Pouyan E. Boukany, Rienk Eelkema

**Affiliations:** a Delft University of Technology, Department of Chemical Engineering Van der Maasweg 9 2629 HZ Delft The Netherlands R.Eelkema@tudelft.nl

## Abstract

We present a new method to obtain tertiary amine-based prodrugs with dual functionality, enabling (i) signal-triggered drug activation and (ii) covalent incorporation in polymer materials through a clickable azido-group unit on the molecular prodrug scaffold. Using nucleophilic substitution on an electron deficient azido-phenyl allyl bromide scaffold, we were able to obtain prodrugs from a variety of amine drug candidates. Subsequent drug activation was initiated by using S or N-terminal biomarker nucleophiles including amino acids, a neurotransmitter, and glutathione as chemical signals. Hydrogel scaffolds labelled with anti-cancer or antibiotic prodrugs were tested in aqueous and cellular media. Through this strategy, we achieved controlled drug release upon signal activation for *in vitro* cancer models with ∼100% wound closure inhibition of A549 small lung cancer cells. We anticipate that this new strategy for the development of responsive prodrug-conjugate incorporated materials will lead to further advancements in drug delivery and specialized therapeutics.

## Abstract 2

A versatile method for tertiary amine-based prodrugs enables signal-triggered activation and polymer conjugation, allowing controlled, biomarker-responsive release from prodrug-labelled hydrogels with inhibited lung cancer cell migration *in vitro*.

## Graphical abstract

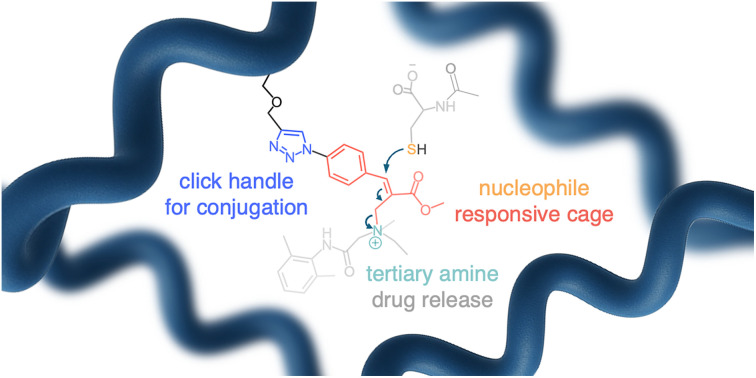

## Introduction

The strategy of linking signal-responsive prodrugs to a carrier material is a promising approach to improve the selectivity of chemotherapy drugs.^1–3^ Besides the improved water solubility, it enables controlled drug release profiles *via* temporal-, or local activation.^4^ Prodrugs or caged drugs are chemically modified therapeutics that are inactive until their activation *via* stimulus-induced removal of the cage group.^5^ In cancer therapy models, a variety of stimuli have been used as triggers for controlled drug activation *via* chemical de-caging, including pH,^6,7^ glutathione (GSH),^8,9^ reactive oxygen species (ROS),^10,11^ hypoxia^12^ or enzymes,^13–15^ amongst others.^16–18^

A common feature found in many prodrug systems to obtain controlled response, is their dependency on linker units such as self-immolative spacers^4^ or a metabolically cleavable connection. Specifically, for self-immolative systems, prodrug constructs require at least one functional group that allows attachment to a targeting or depot scaffold.^5,19^ Several linker types have been developed, including oximes/imines,^20^ hydrazone^21^ and disulfide bonds^22–24^ for a variety of drug candidates. Despite these advancements, many synthetic challenges remain, especially the incorporation of prodrugs into a carrier material. Indeed, the majority of prodrugs rely on carrier systems for water solubility.^5^ So far, material development for prodrugs is based either on non-covalent linkage, including encapsulation,^25,26^ self-assembly^26–28^ or is achieved through complex synthesis procedures, which is often limited to polymer conjugates^29,30^ or dendrimer systems.^13^

**Fig. 1:**
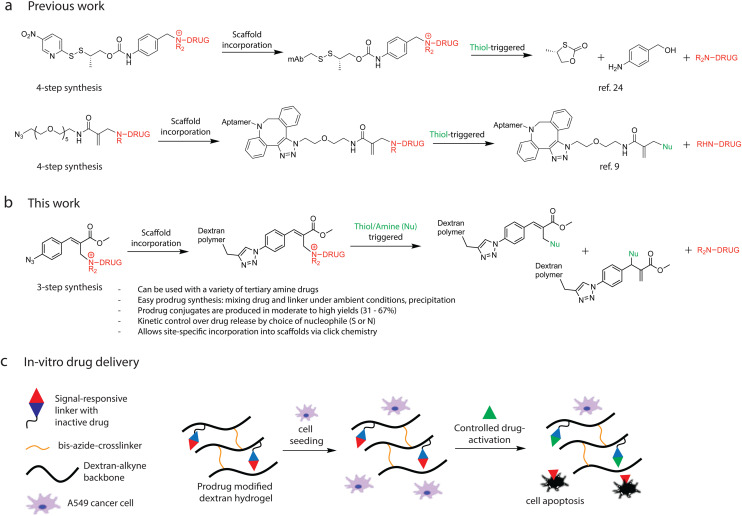
Overview of signal-responsive prodrugs using thiol trigger. (a) Previous work by Pillow *et al.* utilizing self-immolative spacer to form quaternary ammonium conjugates with tertiary or heteroaryl amine drugs, which attached to antibodies (mAb) and are designed to be triggered by glutathione (GSH)-induced thiol exchange, followed by intramolecular cyclization and a 1,6-elimination reaction to release the active drug. Previous work by Thayumanavan *et al.* utilizing amide-based, clickable Michael acceptor to form aptamer-drug conjugates, which release incorporated NH-drugs *via* thiol-mediated nucleophilic substitution. (b) This work showing the signal responsive prodrug-linker used as molecular scaffold on hydrogel backbone and tertiary amine drug release upon thiol-trigger activation. (c) Alkyne modified dextran is conjugated with signal-responsive prodrugs and crosslinked with a bis-azide crosslinker using standard Cu-click conditions. Biological signals trigger release and activation of anti-cancer drugs from the hydrogels, leading to cancer cell proliferation inhibition or apoptosis.

An alternative concept to achieve signal-reversible prodrugs was introduced by Pillow and co-workers.^24^ The authors developed a drug delivery platform that employs a self-immolative spacer to form quaternary ammonium conjugates with tertiary or heteroaryl amine drugs. These conjugates can be site-selectively attached to antibodies or other biologically active carrier molecules and are designed to be triggered by glutathione (GSH)-induced thiol exchange, followed by intramolecular cyclization and a 1,6-elimination reaction to release the active drug (Fig. 1a – previous work). More recently, S. Thayumanavan and co-workers^9^ utilized an NH-containing drug (desmethyltamoxifen) in combination with an amide-based, clickable Michael acceptor linker to prepare aptamer–drug conjugates. These conjugates are designed to undergo thiol-triggered substitution, enabling controlled drug release from the aptamer scaffold (Fig. 1a – previous work). Although these are fascinating examples of controlled drug release, they involve lengthy synthetic procedures including considerable purification procedures. A generic, scalable, and straightforward strategy for coupling linker and drug components would offer significant advantages for the development and upscale of signal-responsive prodrug systems. Consequently, we sought to use our previously reported chemistry on Morita–Baylis–Hillmann (MBH)-adducts^31–37^ to develop a new tertiary amine-prodrug platform with biomarker signal-triggered drug activation for controlled delivery. Earlier work began with small-molecule reactions targeting polymer-bound tertiary amines forming quaternary ammonium ions and enabling dynamic control over material properties through chemical reaction networks (CRNs) such as micelle assembly/disassembly and hydrogel swelling/deswelling,^31^ coacervate phase formation and collapse,^34,35^ transient stiffness modulation in coacervate injectable gels,^33^ and even signal-amplified hydrogel degradation.^32^ Importantly, these modular CRNs have been designed to support stimuli-specific deactivation, allowing materials to respond either autonomously, self-amplifying or with single trigger addition *via* thiol or amine inputs. This work draws from the same fundamental chemistry, tertiary amine quaternization followed by nucleophilic substitution, but pivots towards the covalent incorporation of the quaternarized prodrug-scaffold into polymeric materials enabling multifunctional prodrug-carrier development with signal-triggered drug activation.

Significantly, this system constitutes a straightforward and widely applicable method for the formation of stable quaternary amine prodrug conjugates from various amine-containing therapeutics, without the need for long or cumbersome synthetic routes. By using the embedded azide functionality on the prodrug together with an alkyne-functionalized dextran and a bis-azide crosslinker, we developed prodrug conjugated hydrogel structures to demonstrate its material compatibility. The traceless cleavage of conjugated drugs in the material matrix is realized upon the addition of nucleophilic biomarker signals, leading to controlled drug activation (Fig. 1b). We incorporated anticancer prodrugs in a hydrogel and used this material to inhibit *in vitro* A549 cancer cell proliferation to demonstrate signal-controlled drug release (Fig. 1c).

## Results and discussion

### Prodrug synthesis and kinetic control over drug release

**Fig. 2:**
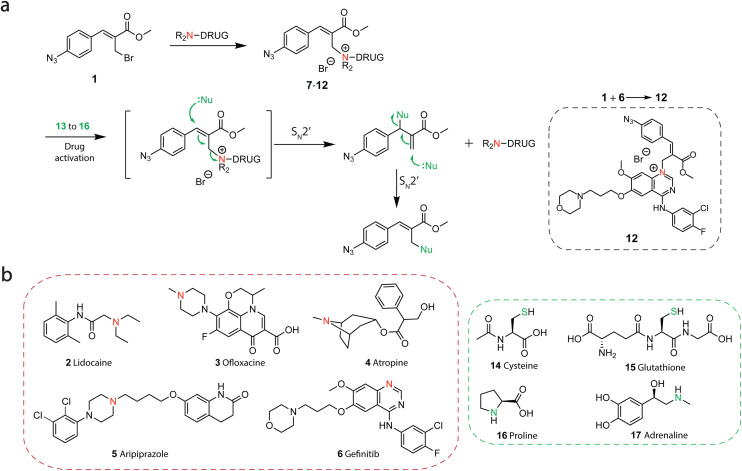
Nucleophile-triggered prodrug development, structures of used therapeutics and biological signals for drug activation (a) Prodrug synthesis (8 to 12). (b) Chemical structure of drugs (2–6): lidocaine (anesthetic), aripiprazole (anti-psychotic), ofloxacine (antibiotic), atropine (muscle-relaxant), gefitinib (anti-cancer) that contain tertiary-amines as reactive groups (red) for prodrug development and biological signals (14 to 17) with N-or S terminal reactive groups (green) used to activate drug release from prodrugs (8 to 12) *via* two consecutive S_N_2′ nucleophilic substitution reactions.

For the development of signal-responsive prodrugs, we envisioned the connection of tertiary amine therapeutics through quaternary ammonium salts by using nucleophilic substitution on electron deficient MBH-bromides (Fig. 2). Our group recently demonstrated that MBH-acetates together with tertiary nitrogen nucleophiles form metastable, positively charged quaternary nitrogen adducts in buffered aqueous solution^31,34,35^ and outlined their potential for biomedical applications. To realize quaternary ammonium salt prodrugs, we designed a three-step synthesis route. We synthesized the clickable allyl bromide scaffold 1 through an MBH reaction between methyl acrylate and 4-azidobenzaldehyde and subsequent bromination of the product using phosphorous tribromide (see experimental details in SI). Tertiary amine drugs 2–6 spontaneously react with 1 under ambient conditions, followed by straightforward isolation and purification by precipitation in THF to give prodrug conjugates 8–12 in moderate to good yields (Fig. 2a). We demonstrate the generality of the concept using a broad range of different therapeutics, including an anti-cancer drug (6), but also an antibiotic (3), muscle relaxant (4), anti-depressant (5) and an anesthetic (2) (Fig. 2b).

**Fig. 3:**
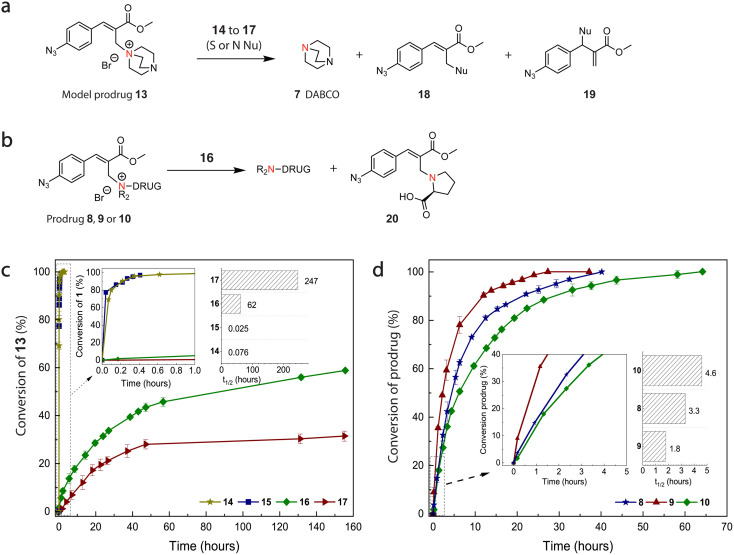
Small molecule release study from model prodrug 13 using different biological signals (14 to 17) and kinetic drug release study from different prodrugs (8, 9 and 10) using l-proline (16). (a) DABCO 7 release from 13 using signal 14 (*N*-acetyl-cysteine), 15 (l-glutathione), 16 (l-proline) or 17 (l-adrenaline). (b) Drug release from prodrugs 10, 9 and 8 using 16. Conversion of the reactants was monitored by ^1^H NMR over time in DMSO-d6/phosphate buffer (pH = 7.4, 0.1 M) 1 : 9 (DABCO release) and 3 : 7 (drug release) at room temperature. (c) DABCO 7 release (SI Fig. 1–4) from prodrug model 13 (39 mM, 1.0 eq.) with signals 14–17 (1.0 eq.). (d) Drug (2, 3 and 4) release (SI Fig. 5–7) using signal 16 from corresponding prodrugs: 16 (9.6 mM, 1.20 eq.) addition to prodrugs 8, 9 and 10 (1.0 eq.). The error bars represent the standard deviation of duplicate measurements.

The addition of S or N-terminal nucleophiles (signal 14–17, Fig. 2b) on the prodrug conjugates reverses the quaternary nitrogen back to the neutral tertiary amine, releasing and thereby activating the drug molecule. Importantly, this strategy enables traceless drug release. The substitution of the nucleophile on quaternary ammonium salts is proposed to proceed *via* two consecutive S_N_2′ reactions,^38,39^ resulting in clean elimination of the linker and release of the free drug. As signals, we employ a broad range of biological nucleophiles, including *N*-acetyl cysteine (signal 14), GSH (signal 15), l-proline (signal 16) or l-adrenaline (signal 17). To examine whether our linker design would allow traceless release of a tertiary amine upon reaction with a nucleophile signal, we combined prodrug precursor 1 with tertiary amine DABCO (7) as model drug to form prodrug 13 (Fig. 3a). Here, prodrug 13 was treated with thiol signals (14 and 15) and secondary amine signals (16 and 17) and followed with ^1^H NMR spectroscopy over time. Exposure of prodrug 13 (39 mM in 1 : 9 DMSO-d_6_/phosphate buffer (pH = 7.4, 0.1 M)) to 14 and 15 (1.0 eq.) led to thiol-mediated nucleophilic substitution and generation of free DABCO7 within minutes (SI Fig. 1 and 2).

As a result of the substitution with various nucleophiles (14–17), nucleophile products 18–20 are formed in the reaction. We observed differences in the configuration of the allyl products depending on whether S- or N-terminal nucleophiles were used. When employing NH-nucleophiles **16** and **17**, the reactions yield exclusively the thermodynamically more stable allylic isomers featuring internal double bonds.^38^ In contrast, reactions with strong SH-nucleophiles **14** and **15** selectively produce the kinetically favored allylic isomers with terminal double bonds. Notably, these kinetically controlled products can be subsequently displaced by the same nucleophile (*e.g.* SH-nucleophile is in excess), resulting in the thermodynamically more stable allylic isomers bearing internal double bonds.^38^

Similarly to previous research,^40^ we found that the reactivity of *N*-acetyl cysteine (14) and GSH (15) was approximately equal with their half-life being 4.5 and 1.5 min, respectively. In contrast, N-terminal nucleophiles 16 and 17 (1.0 eq., SI Fig. 3 and 4), showed much slower release kinetics compared to thiols with conversions reaching 53 and 35% after 160 hours and half-lives of 62 and 247 hours, respectively (Fig. 3c and insert). These kinetic differences are attributed to the difference in nucleophilicity between thiols and secondary amines^41^ and confirm our previous findings.^31,35^

After confirming the nucleophile-triggered model drug release of free DABCO **7** from prodrug 13, we studied the release of amine drug variations from prodrugs using 16 (Fig. 3b). Initial trials showed that prodrug-candidates 11 and 12 were not soluble using our standard protocol with DMSO-d_6_/phosphate buffer = 3 : 7 and required a too large fraction of non-aqueous solvent for dissolution, reducing the use of release experiment data. However, prodrug 8, 9 and 10 (including atropine 4, ofloxacine 3 and lidocaine 2) were soluble and could be studied further in the small molecule release experiments. Upon introduction of 16 to the system (prodrug : signal = 1 : 1.2), we observed the complete release of amine drugs, alongside the formation of nucleophile product 20 within 65 hours (Fig. 3d) with their half-lives being *t*_1/2_ = 4.6, 3.3 and 1.3 hours for prodrugs 10, 8 and 9, respectively. We assume that these differences in their release kinetics are related to the drugs aqueous solubility, as seen in other drug delivery systems.^42^

### Prodrug material incorporation and drug activation in dextran-based hydrogels

**Fig. 4:**
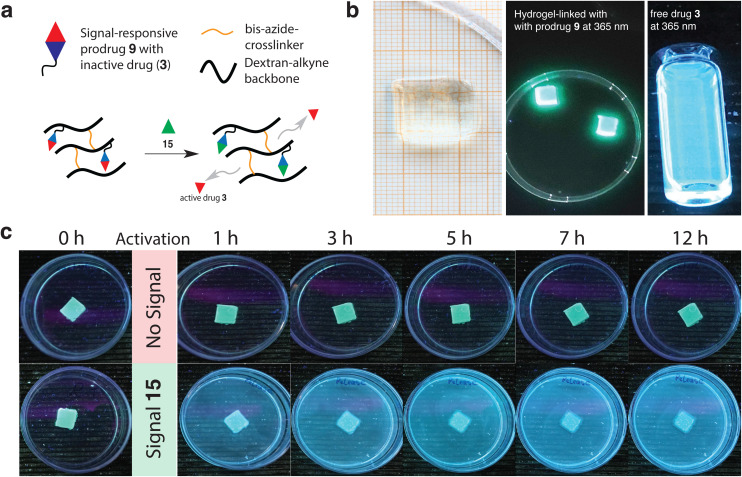
Prodrug linker incorporation in hydrogel scaffold and signal-activated drug release. (a) Schematic representation of signal activated drug release from dextran-based hydrogel scaffolds using 15. (b) (left) Photograph of transparent self-supporting dextran-based hydrogel modified with prodrug 9, (middle) dextran-based hydrogels modified with prodrug 9 under 365 nm UV-light in DMSO : PB = 1 : 9 solution, (right) free drug 3 in DMSO : PB = 1 : 9 solution under 365 nm UV-light. (c) Signal-triggered drug 3 release from dextran-based hydrogel patch using 15, visualized under 365 nm UV light.

After successful drug activation from signal-responsive prodrugs, we investigated their material compatibility to alkyne-substituted hydrogel scaffolds and drug release during signal activation by visual inspection of fluorescence intensity changes (Fig. 4a). To assess concentration changes upon signal-triggered drug release, we used drug 3 (ofloxacin) as model. Free ofloxacin exhibits strong blue emission under 365 nm UV irradiation, whereas its prodrug analogue emits green fluorescence (SI Fig. 10), due to the quaternization of the piperazinyl motif on ofloxacin.^43^ This allows the visual distinction between prodrug 9 and free drug 3 under UV-light (Fig. 4b).

Dextran-based hydrogel scaffolds containing drug 3 were constructed using a two-step Cu-click reaction procedure. First, reaction of alkyne-dextran (500 kDa, degree of substitution (DS) = 41%, SI Fig. 8) with 0.1 eq. prodrug 9*vs.* free alkyne units, using CuBr and activating ligand tris(3-hydroxypropyltriazolylmethyl)amine (THTPA) in DMF, gave prodrug-modified, alkyne-dextran chains (SI Fig. 9). We then reacted this construct with PEG-bis-azide crosslinker (poly(ethylene glycol) bisazide, *M*_n_ = 1100 g mol^−1^) in H_2_O using CuSO_4_, sodium ascorbate and THTPA, which resulted in gelation after 30 min. This procedure afforded transparent, self-supporting hydrogel objects with dimensions of 1.1 × 1.1 × 0.5 cm (Fig. 4b). The morphology of the hydrogels was analyzed by scanning electron microscopy (SEM) before and after prodrug modification.

Besides a slightly smaller pore size, the interior morphology of the hydrogels showed no significant differences between hydrogels with prodrug and compared to non-modified control hydrogels (SI Fig. 11). After several washing steps, the prodrug modified hydrogels continued to exhibit green fluorescence, indicating the presence of the covalently attached prodrug. Encouraged by these results, we started to qualitatively investigate drug activation from hydrogels by adding ∼100 µM signal 15 to our prodrug-modified hydrogel patch and compared it to a non-signal activated hydrogel as control (Fig. 4c). Visual inspection of the hydrogels over 12 hours at specific time intervals under 365 nm UV light revealed a clear distinction between signal-activated and non-activated hydrogels, as well as their time-dependent fluorescence increase.

### *In vitro* – signal-activated drug release from polymeric hydrogels

**Fig. 5:**
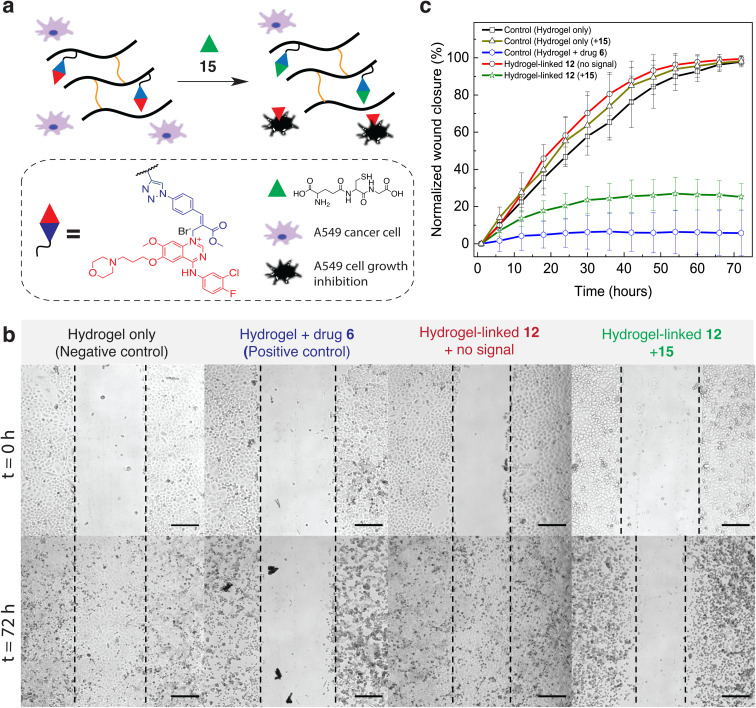
*In vitro* signal-activated drug release from polymeric hydrogels. (a) Schematic representation and components of the signal-triggered drug activation from dextran-based, prodrug incorporated hydrogels in the presence of A549 lung cancer cells. (b) Brightfield microscopy pictures at *t* = 0 h and *t* = 72 h from wound healing assay (black dotted lines are added for comparison). Hydrogel/reactants and additives (i) hydrogel, no prodrug/1% DMSO, (ii) hydrogel, no prodrug/100 µM drug 6 in 1% DMSO, (iii) hydrogel with 5.0 mM prodrug 12/1% DMSO and (iv) hydrogel with 5.0 mM prodrug 12/1%DMSO and 400 µM 15. Scale bar inserts = 250 µm. (c) Normalized wound closure (%) calculated from cell proliferation assay observation based on cell density changes over 72 hours observation period. The error bars represent the standard deviation of triplet measurements.

To demonstrate drug release from the prodrug-conjugated hydrogels *via* controlled signal activation in live cell media, we supplied hydrogel patches containing the caged anticancer drug gefitinib (drug 6, Fig. 5a ‘red’) to A549 lung cancer cells (purchased from ATCC) and subjected these to 15 (GSH, Fig. 5a ‘green triangle’) (Fig. 5a). GSH plays an important role in the cellular redox balance but also in cancer cell proliferation.^44^ Furthermore, GSH has been found at elevated levels in various human cancer tissues,^44,45^ which makes it a prime candidate for signal-triggered drug delivery.^46^ Gefitinib is an FDA approved reversible epidermal growth factor receptor (EGFR) tyrosine kinase inhibitor, which is used for treating advanced non-small cell lung cancer, from which A549 cells are derived.^47,48^

We evaluated cell proliferation in the presence of prodrug-conjugated hydrogel patches during 72 hours wound healing assay *via* brightfield microscopy at incubator conditions. Proliferation results are shown in Fig. 5b, at *t* = 0 h and *t* = 72 h, where cellular wound closure can be used to qualitatively identify cell proliferation (wound closure) or inhibition (no wound closure) and furthermore quantify the change in cell density during proliferation *via* machine learning based (bio)image analysis software – ‘Ilastik’ (Fig. 5c). As expected, control experiments including a negative control (hydrogel only, no prodrug + 1% DMSO) and a positive control (hydrogel, no prodrug + 100 µM drug 6 in 1% DMSO) showed 97.8 ± 2.0% and 5.4 ± 12.3% wound closure after 72 h, respectively. The dextran-based hydrogel patch and 1% DMSO also show no cytotoxicity towards A549 cells. On the contrary, addition of 100 µM (46 µg mL^−1^) of drug 6 leads to overall ∼94% cell proliferation inhibition at the end of the observation period (Fig. 5b – positive control), which agrees with literature values.^49^ Next, we observed cell proliferation in the presence of hydrogel patches containing 5.0 mM conjugated prodrug 12 (0.04 eq. 12*vs.* alkyne units) and with or without signal-trigger drug activation (400 µM of 15). From the experimental results, we found a gradual wound closure increase within the first ∼36 h. Remarkably, this was followed by a plateau with maxima at 25.2 ± 7.0% wound closure from 36 to 72 h (Fig. 5b/c, hydrogel-linked 12, + signal 15, SI Movie). Significantly, A549 cells in the presence of hydrogels and treated without GSH signal displayed 99.4 ± 1.0% wound closure, which confirms the absence of drug leakage or cytotoxic background interference (Fig. 5b/c, hydrogel-linked 12, no signal).

We observed a lag time (∼36 h, change in avg. cell density <1%) in the wound healing assay during signal-triggered drug release, which is likely linked to the kinetics of the nucleophilic substitution reaction limited by diffusion to prodrug 12, and similarly to the diffusion of drug 6 from the hydrogel matrix. Indeed, such diffusion limitations related drug release lag time from a hydrogel matrix is often observed.^50^ Additionally, we performed cell proliferation tests using only hydrogel, no prodrug and 400 µM of 15 (SI Fig. 12), which showed no significant changes in cell proliferation compared to the positive control (Fig. 5c).

## Conclusions

We herein introduce signal-responsive drug release from hydrogel scaffolds using nucleophilic signal sensitive prodrug linkers. This proof of concept is based on electron deficient azido-phenyl allyl bromides, which enable facile prodrug development *via* nucleophilic substitution of tertiary amine drugs. An azido functionality on the molecular scaffold enables straightforward covalent conjugation to alkyne-substituted polymer gels. Simply mixing of clickable linker 1 with tertiary amine drugs in THF results in precipitation of prodrug products and circumvents lengthy or cumbersome synthesis procedures. Using this strategy, we were able to obtain prodrugs from a variety of drug precursors, *e.g.* anti-cancer, antibiotic, muscle relaxant, anti-depressant or anesthetic, making this method not only easy to use but generally versatile and potentially widely applicable. We obtained control over drug activation kinetics by using S or N-terminal chemical signals, with their order being l-glutathione ≈ *N*-acetyl cysteine ≫ l-proline > l-adrenaline. Signal-triggered release experiments using visual fluorescence increase gave clear indication of substantial drug release upon signal activation compared to non-triggered gels. Finally, we successfully demonstrate this strategy by GSH-triggered activation of an anticancer drug from a prodrug hydrogel construct in the presence of A549 cancer cells and observed drug release induced cell growth inhibition of ∼74.8 ± 7.0% after 72 h during wound healing experiments. The kinetic differences between nitrogen and sulfur nucleophiles, coupled with their distinct distribution in biological environments, offer complementary avenues for drug delivery design. Amine-mediated reactions, characterized by slower kinetics, are well suited for intercellular applications such as polymer hydrogel depots, enabling sustained release over extended time scales. In contrast, thiol-triggered processes use the higher intracellular abundance and faster reactivity of sulfur nucleophiles, providing an efficient mechanism for rapid drug release from microgel particles, polymer therapeutics, or biomacromolecular conjugates. These findings underscore the potential of nucleophile-responsive platforms to be tailored toward either long-term systemic delivery or rapid intracellular activation, depending on the therapeutic need.

## Conflicts of interest

The authors declare no conflicts of interest.

## Supplementary Material

LP-004-D5LP00317B-s001

LP-004-D5LP00317B-s002

## Data Availability

The data supporting the findings of this study can be found in the supplementary information (SI). Supplementary information: material and methods, NMR kinetics, precursor synthesis, hydrogel preparation and characterization, pro-drug activation/release studies with A549 cells and synthesis procedures including ^1^H and ^13^C NMR of the intermediates and products as well as the 2D NMR and LC-MS data. Supplementary movie: cell proliferation in the presence of hydrogel patches with conjugated prodrug and with signal-trigger drug activation. See DOI: https://doi.org/10.1039/d5lp00317b.
