# Supplementary material for: Nucleophile-triggered prodrug release from polymer hydrogels
Source: RSC Appl Polym. 2025 Nov 10;4(1):328–35. doi: 10.1039/d5lp00317b (PMC12621203; doi:10.1039/d5lp00317b)
Supplement: LP-004-D5LP00317B-s001 [file LP-004-D5LP00317B-s001.pdf]

Supplementary Information (SI) for RSC Applied Polymers.
This journal is © The Royal Society of Chemistry 2025

Supporting Information

Nucleophile-triggered prodrug release from polymer hydrogels

Benjamin Klemm[a], Magherita Tavasso[a], Irene Piergentili[a], Max Satijn[a], Tobias G. Brevé[a], Pouyan E. Boukany[a] and Rienk Eelkema[a,*]

[a] Delft University of Technology, Department of Chemical Engineering, Van der Maasweg 9, 2629 HZ Delft, The Netherlands.

* Correspondence to: R.Eelkema@tudelft.nl

S1.0 Materials and Methods

S1.1 Instrumentation, materials and characterization

All reagents and solvents were used without further purification unless otherwise stated. Ofloxacine (≥99%, **3**), atropine (≥99%, **4**), aripiprazole (>98.0%, **5**), gefitinib (>98.0%, **6**), lidocaine (≥99%, **2**), DABCO (≥99%, **7**), dextran (500 kDa), glycidyl propargyl ether, poly(ethylene glycol) bisazide (average M_n 1100 Da), L-proline (**14**, ≥99%), N-acetyl cysteine (**15**, ≥99%), L-adrenaline (**16**, >98.0%), L-glutathione (**17**, ≥98%), 4-formylphenylboronic acid (≥95.0%), methyl acrylate (99%) and phosphorous tribromide (99%) were purchased from Sigma Aldrich or TCI Europe. For the preparation of aqueous buffers, solid salts were used: sodium phosphate monobasic and sodium phosphate dibasic, purchased from Sigma Aldrich. Unless stated otherwise, all stock solutions were prepared in d_6-DMSO/phosphate buffer mixture 1:9 (0.1 M, pH = 7.4). All buffers were pH adjusted using sodium hydroxide (1 M) and hydrochloric acid (1 M). ESI-MS was performed using LTQ XL spectrometer equipped with Shimadzu HPLC setup operating at 0.2 mL/min flow rate with water/MeCN mobile phase containing 0.1 vol% formic acid and Discovery C18 column. Photographs of the hydrogels were taken on a Canon EOS 600D single reflex camera with a Canon Macro Lens EF 100 mm 1:2.8 USM.

S1.2 NMR spectroscopy

NMR spectra were recorded on an Agilent-400 MR DD2 NMR instrument at 25°C (399.7 MHz for ^{1}H, 100.5 MHz for ^{13}C and 376 MHz for ^{19}F) using residual solvent signals as internal reference. Sodium trimethylsilylpropanesulfonate (DSS) was used as internal standard for NMR kinetic experiments with reference resonance at 0 ppm. To suppress the water peak, PRESAT or ES_suppression configuration (suppress one highest peak) was used. NMR spectra were processed by MNova NMR software (Mestrelab Research).

S2.0 NMR kinetics

S2.1 Prodrug-activation using different biologically relevant signals (general procedure)

13 (2.04 mg, 10 mM, 1.0 eq.) and DSS as internal standard (1.0 eq.) were dissolved in 0.4 mL d_6-DMSO/phosphate buffer mixture. Then, **14-17** (1.2 eq.) dissolved in 0.1 mL buffer mixture was added to the reaction mixture. The reaction was immediately followed by ^{1}H-NMR.

S2.1.1 Activation of 13 using signal 14 (N-acetyl cysteine)

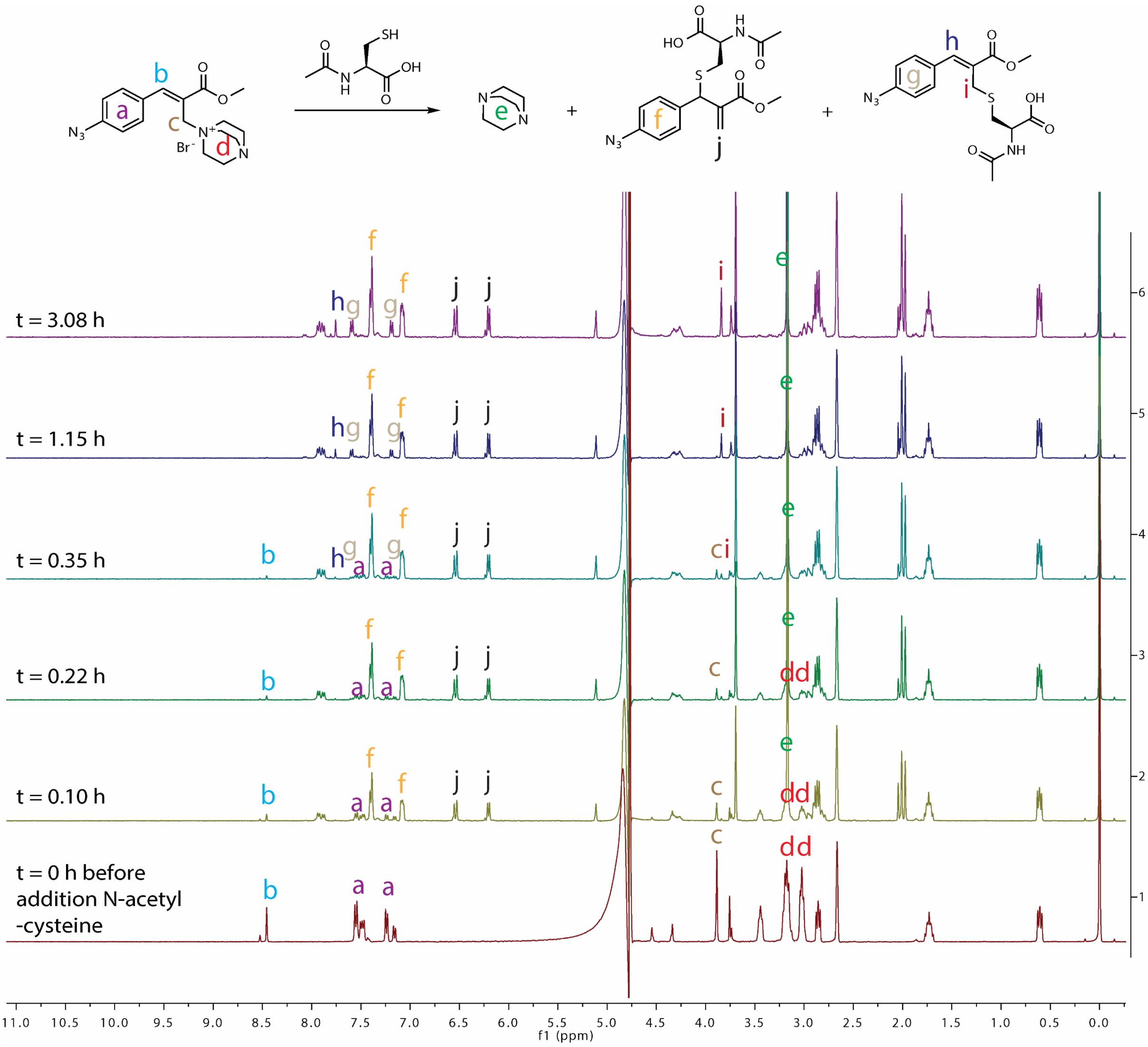

Supplementary Figure 1: Reaction spectra of **13** with signal **14** followed by ^{1}H NMR at different time points. The reaction was carried out in d_6-DMSO/phosphate buffer mixture 1:9 (0.1 M, pH = 7.0). The peak attributed to ~ 0.0 ppm corresponds to DSS internal standard and was used to align the spectra.

S2.1.2 Activation of 13 using signal 15 (L-glutathione)

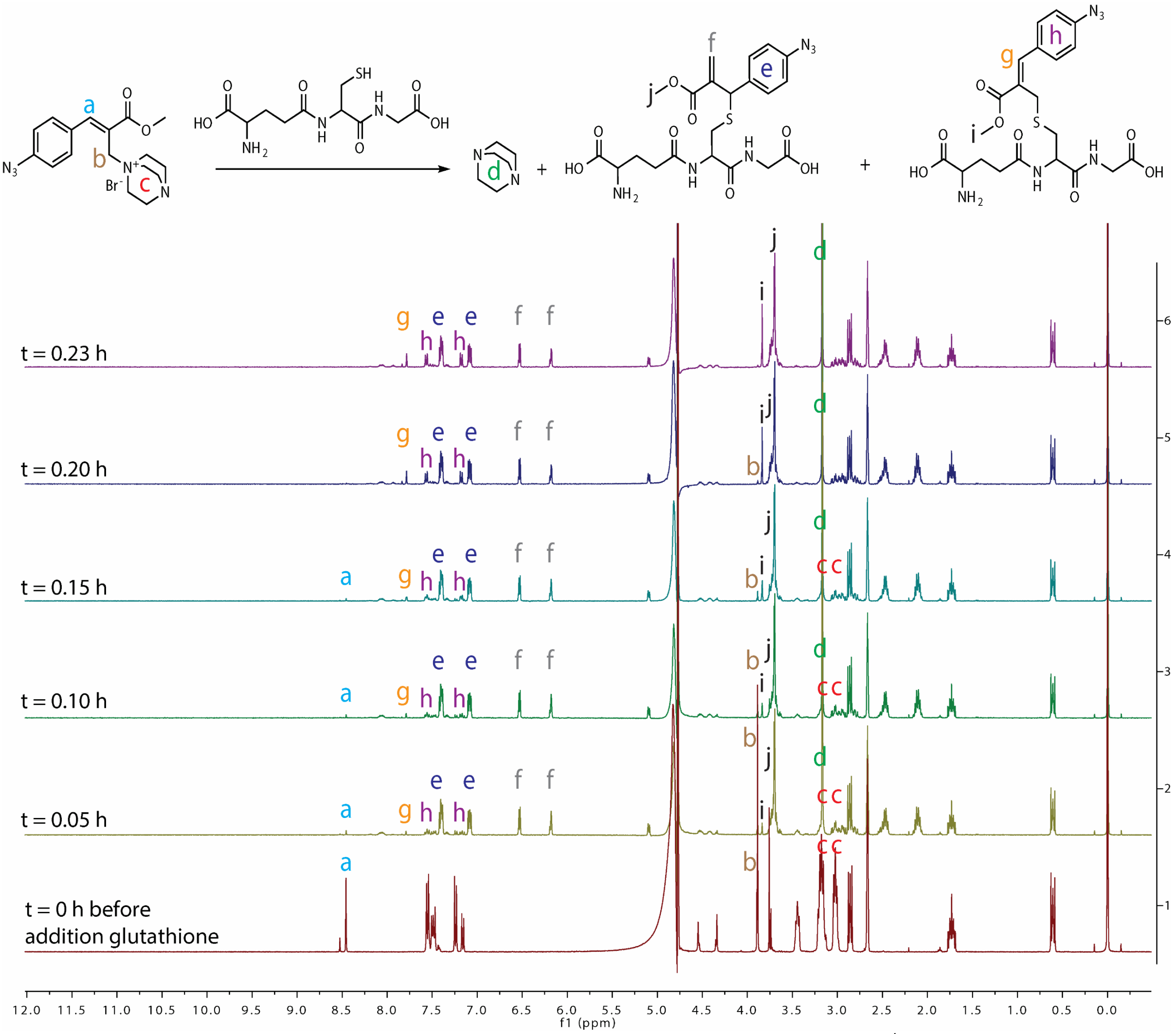

Supplementary Figure 2: Reaction spectra of **13** with signal **15** followed by ^1H NMR at different time points. The reaction was carried out in d_6-DMSO/phosphate buffer mixture 1:9 (0.1 M, pH = 7.0). The peak attributed to ~ 0.0 ppm corresponds to DSS internal standard and was used to align the spectra.

S2.1.3 Activation of 13 using signal 16 (L-proline)

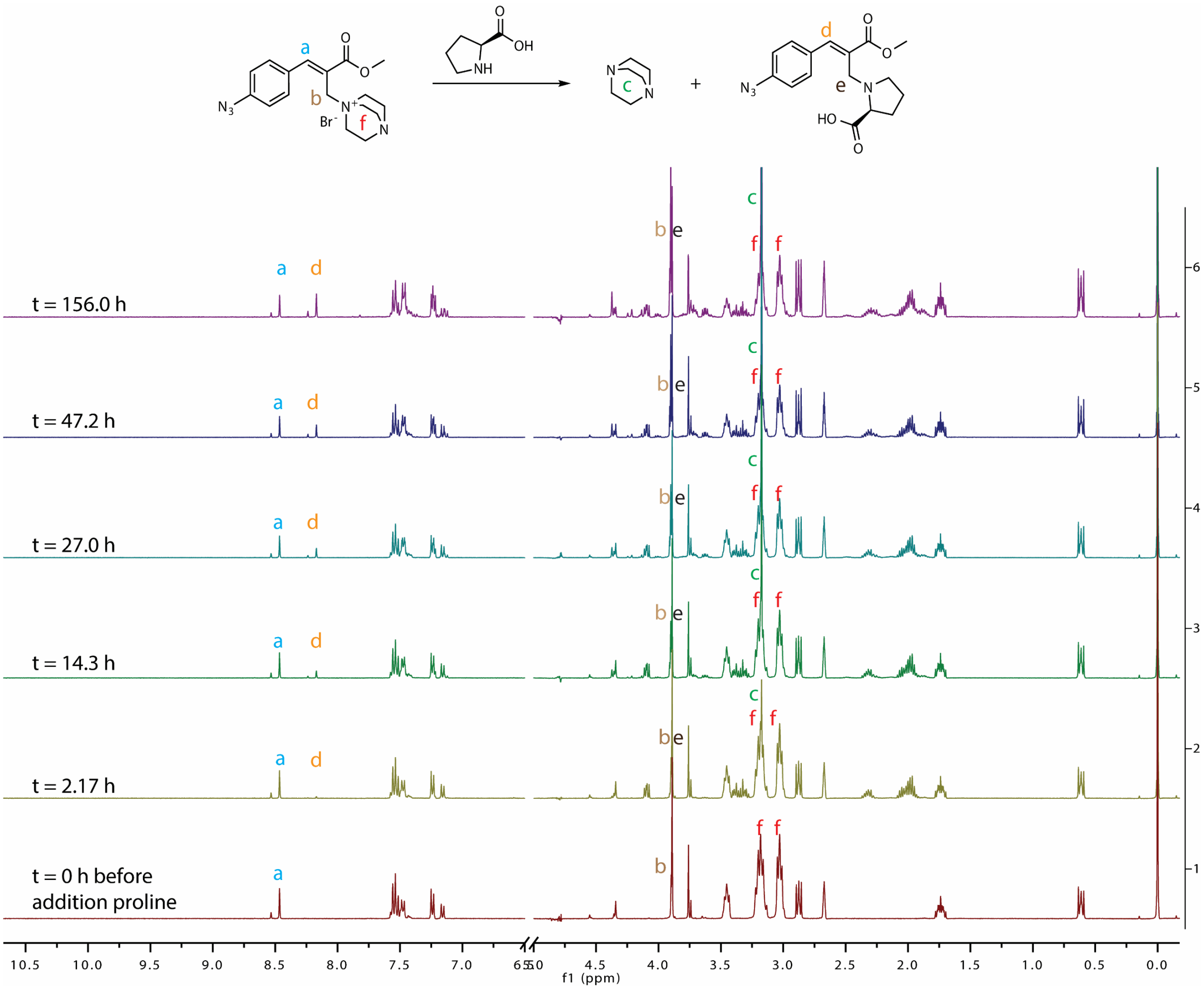

Supplementary Figure 3: Reaction spectra of **13** with signal **16** followed by ^{1}H NMR at different time points. The reaction was carried out in d_6-DMSO/phosphate buffer mixture 1:9 (0.1 M, pH = 7.0). The peak attributed to ~ 0.0 ppm corresponds to DSS internal standard and was used to align the spectra.

S2.1.4 Activation of 13 using signal 17 (L-adrenaline)

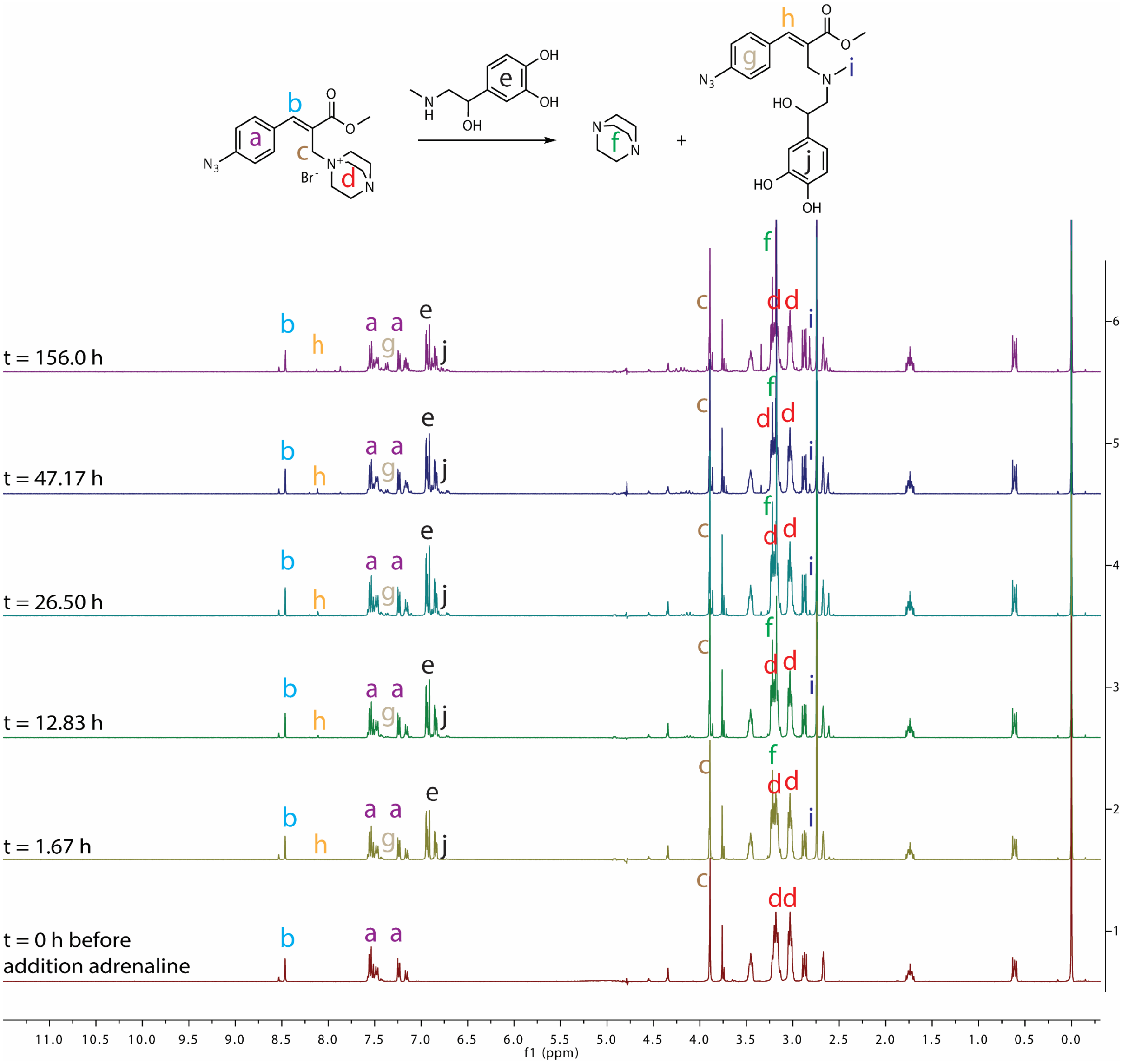

Supplementary Figure 4: Reaction spectra of **13** with signal **17** followed by ^{1}H NMR at different time points. The reaction was carried out in d_6-DMSO/phosphate buffer mixture 1:9 (0.1 M, pH = 7.0). The peak attributed to ~ 0.0 ppm corresponds to DSS internal standard and was used to align the spectra.

S2.2 Activation of prodrugs 8 - 10 using signal 16 (general procedure)

Corresponding prodrug **8**, **9** or **10** (2.1 – 2.6 mg, 8.0 mM, 1.0 eq.) and DSS as internal standard (1.0 eq.) were dissolved in 0.4 mL *d*$_6$-DMSO/phosphate buffer mixture. Then, **16** (1.2 eq.) dissolved in 0.1 mL buffer mixture was added to the reaction mixture. The reaction was immediately followed by NMR.

S.2.2.1 Activation of prodrug 8 – Lidocaine

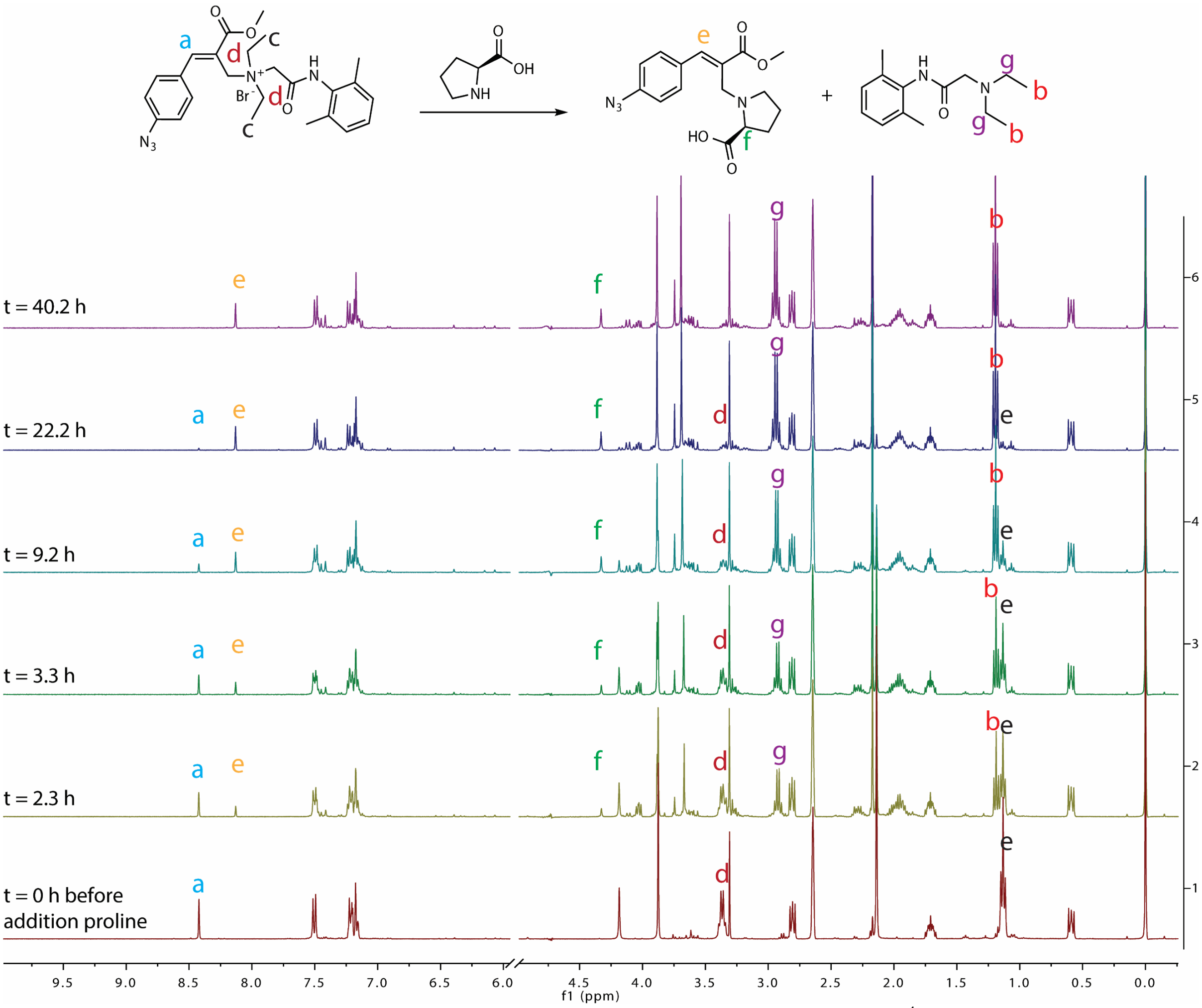

Supplementary Figure 5: Reaction spectra of **8** with signal **16** followed by ^{1}H NMR at different time points. The reaction was carried out in d$_6$-DMSO/phosphate buffer mixture 3:7 (0.1 M, pH = 7.0). The peak attributed to ~ 0.0 ppm corresponds to DSS internal standard and was used to align the spectra.

S.2.2.2 Activation of prodrug 9 – Ofloxacine

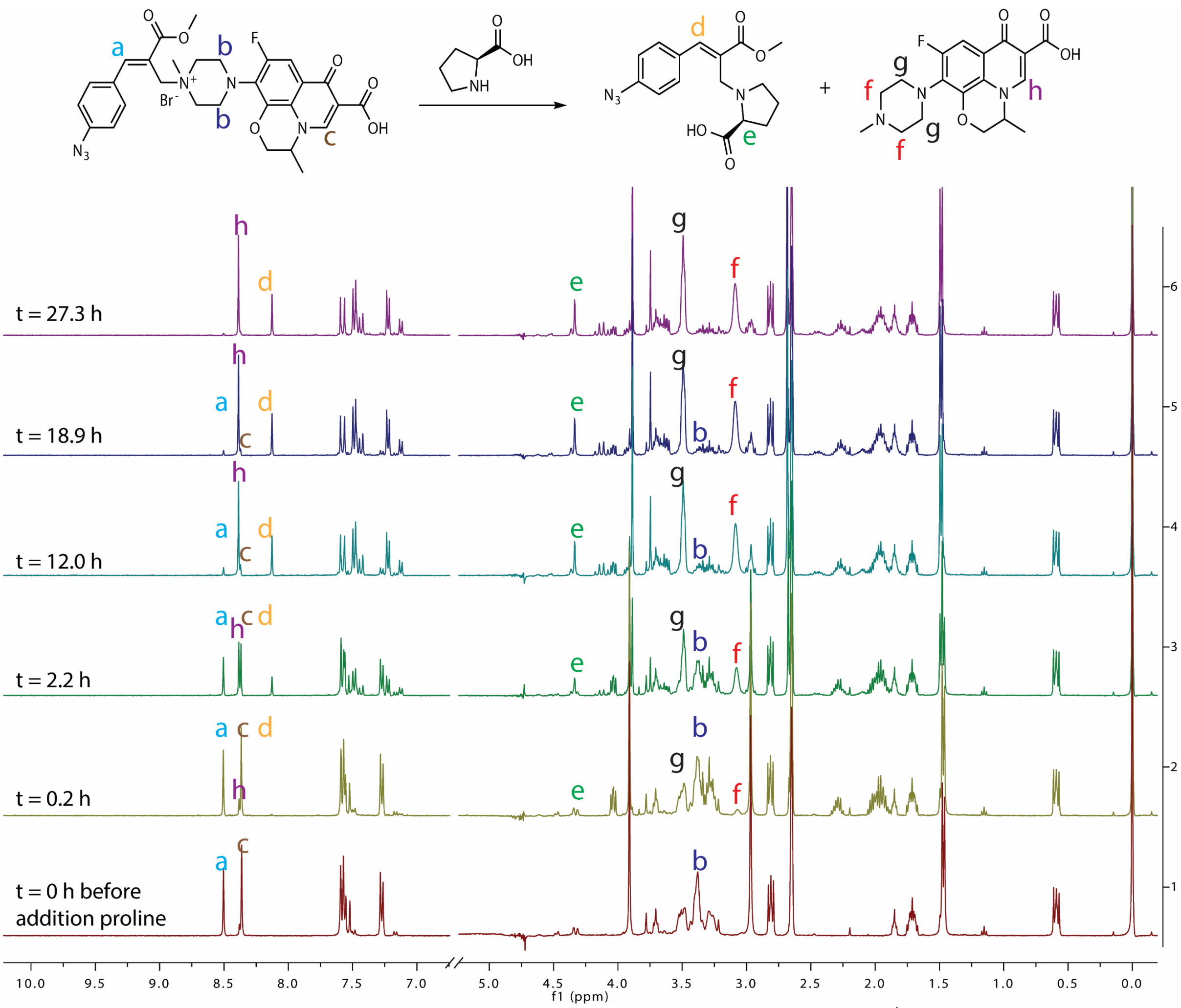

Supplementary Figure 6: Reaction spectra of **9** with signal **16** followed by ^{1}H NMR at different time points. The reaction was carried out in d_6-DMSO/phosphate buffer mixture 3:7 (0.1 M, pH = 7.0). The peak attributed to ~ 0.0 ppm corresponds to DSS internal standard and was used to align the spectra.

S.2.2.3 Activation of prodrug 10 – Atropine

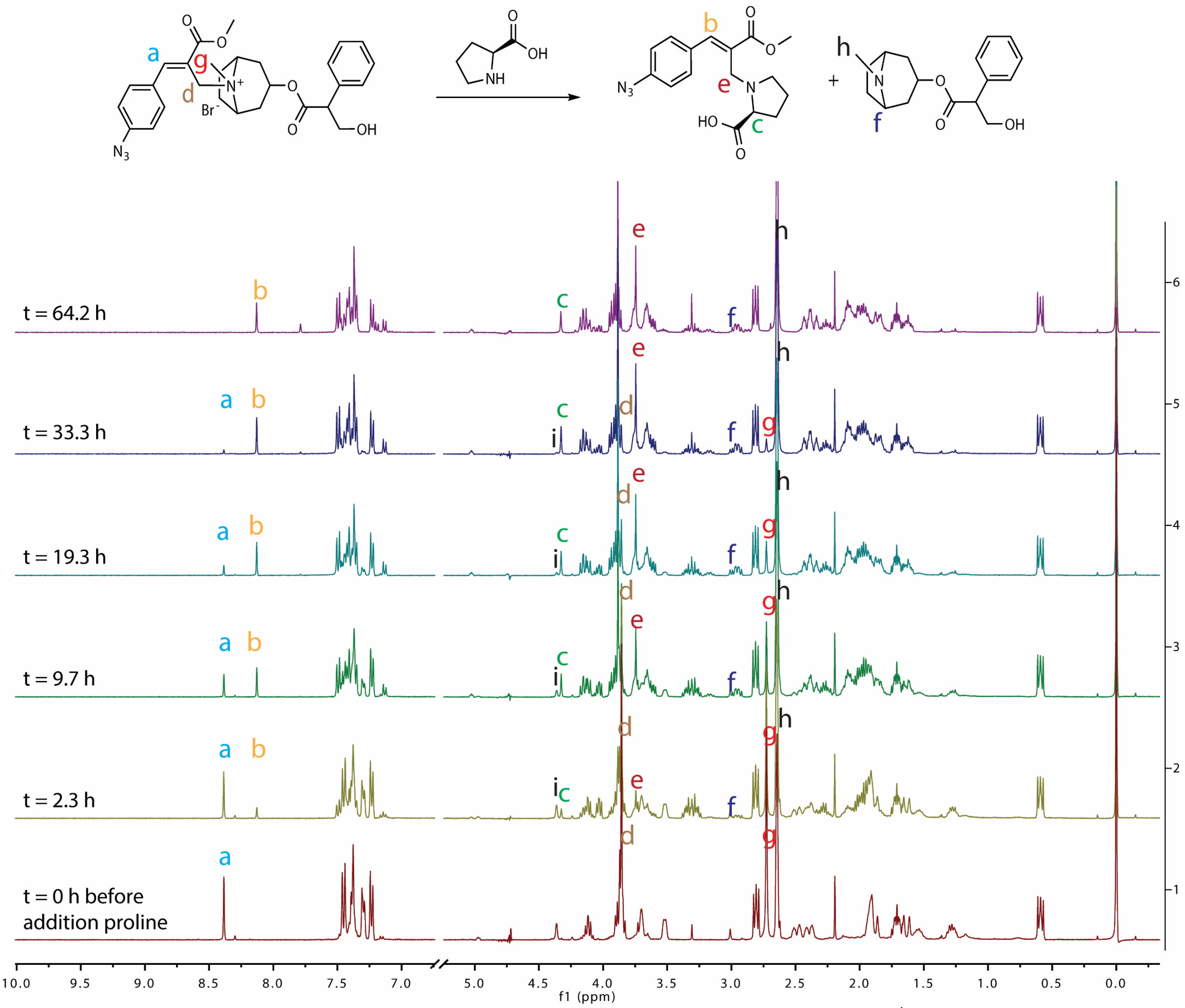

Supplementary Figure 7: Reaction spectra of **10** with signal **16** followed by ^{1}H NMR at different time points. The reaction was carried out in d_6-DMSO/phosphate buffer mixture 3:7 (0.1 M, pH = 7.0). The peak attributed to ~ 0.0 ppm corresponds to DSS internal standard and was used to align the spectra.

S3.0 Dextran-alkyne synthesis

Dextran (500 kDa, 3.4 g, 0.0068 mmol) was dissolved in a NaOH solution (30 ml, 0.1 M) and heated to 35 ºC. Hereafter, glycidyl propargyl ether (5 mL, 52 mmol) is added dropwise and the solution was stirred overnight at 35 ºC. After cooling the reaction mixture to RT, the solution is poured in ethanol (600 mL) to precipitate alkyne modified dextran. The supernatant is decanted and the residue is re-dissolved in 150 mL demineralized water and dialyzed (MWCO = 3.5 kDa) against demineralized water for 72 hours (4 x 2 L). After freeze drying the resultant solution, the pure alkyne modified dextran was obtained as a white fluffy powder.

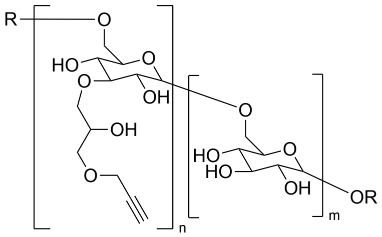

^{1}H NMR (400 MHz, D_2O) δ: 5.49, 5.34, 5.18, 5.00 (anomeric CH, 1H), 4.28 (s, 3H), 4.03 – 3.47 (m, 9H), 2.97 (s, 1H). **^{13}C NMR (**101 MHz, D_2O) δ: 97.66, 79.50, 76.13, 73.33, 71.34, 70.70, 70.11, 69.44, 68.80, 65.45, 58.24

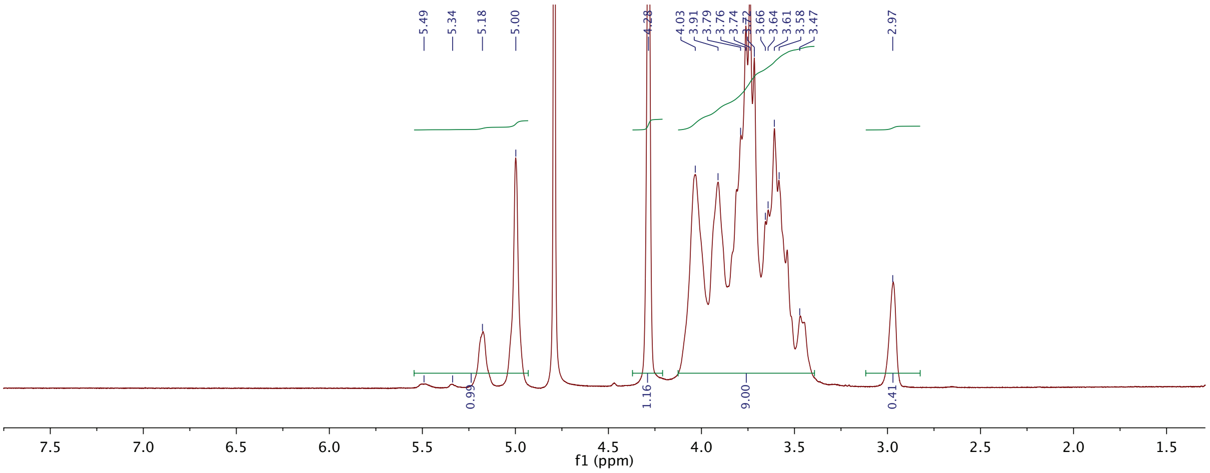

Supplementary Figure 8: ^{1}H-NMR spectrum of alkyne modified dextran (500 kDa) in D_2O. Degree of substitution (DS) = 41% (ratio between alkyne peak (2.97 ppm) and the sum of anomeric proton peaks (5.49 to 5.00 ppm)).

S4.0 Dextran hydrogel preparation

S4.1 Dextran alkyne – chain modification with drug linker 9 and 12 (pre-click)

A 15 wt% dextran alkyne solution was prepared by dissolving 50 mg of dextran alkyne in DMF (0.3 mL) and hereafter shaken for 30 min before further usage. Corresponding drug linker (**9**: 8.0 mg, 0.012 mmol; **12**: 3.6 mg, 0.005 mmol) was solubilized in 0.2 mL DMF and shortly shaken. Next, a Cu-click solution was prepared containing CuBr (0.15 mg, 0.0020 mmol) and tris-hydroxypropyltriazolylmethylamine (THTPA) (0.25 mg, 0.0006 mmol). After degassing both solutions with argon for 10 min, the Cu-click solution (50 µL) was added to the dextran alkyne solution, covered with an argon blanket and then shaken vigorously for 48 hours.

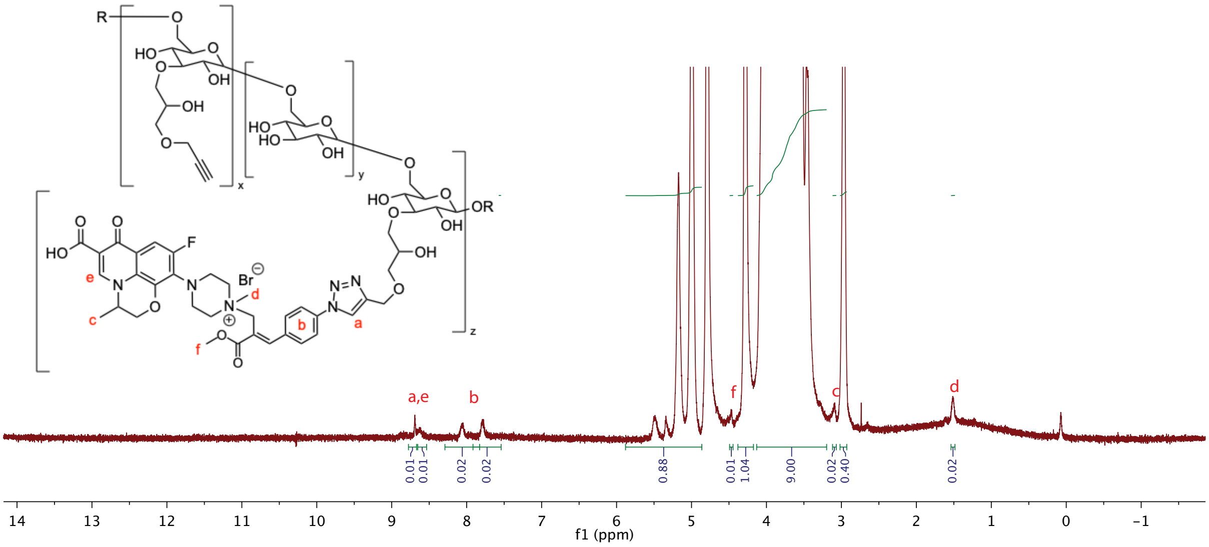

Supplementary Figure 9: ^{1}H-NMR spectrum of ofloxacin modified alkyne-dextran (500 kDa) in D_2O.

S4.2 Dextran alkyne – chain crosslinking (post-click)

A Cu-click solution was prepared by solubilizing $CuSO_4$ (0.15 mg, 0.0020 mmol), sodium ascorbate (1.2 mg, 0.0061 mmol) and THTPA (0.25 mg, 0.0006 mmol) in H_2O. Next, crosslinker (poly(ethylene glycol) bisazide, M_n = 1,100 g/mol, 5.0 mg, 0.0047 mmol) was dissolved in 0.15 mL DMF, added to the pre-clicked dextran-alkyne solution and shaken shortly before the Cu-click solution (50 µL) was added. This mixture contains an approximately 8–9-fold excess of dextran alkyne groups relative to crosslinker azides, to drive the crosslinking reaction to completion. Immediately, after addition, the solution was transferred into a mold where it was left for gelation for 30 min. After gelation was complete, the hydrogel was removed from the mold and placed into a dialysis membrane (MWCO = 8 kDA) where it was dialyzed for at least 6 h each, against DMSO, then against H_2O (2x) for 6 h, then EDTA aqueous solution (0.05 M) and again against H_2O, before further usage (Supplementary Figure 10).

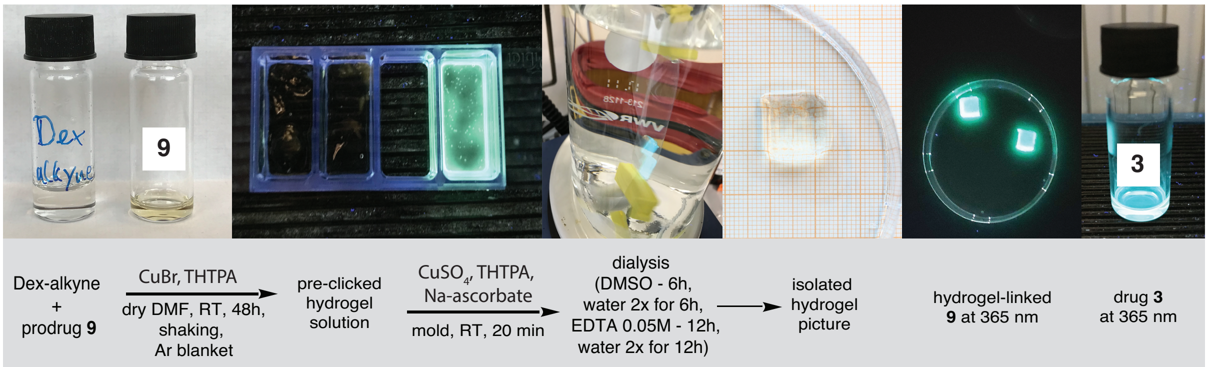

Supplementary Figure 10: Drug-modified dextran-alkyne hydrogel fabrication procedure.

S4.3 SEM analysis of dextran alkyne with/without chain modification using drug linker 9

Hydrogels were prepared using the procedure described in section 4.2 using alkyne-modified dextran for control hydrogels. For prodrug-modified hydrogels, prodrug incorporated alkyne-dextran was used. After their preparation, both hydrogels were submerged in liquid nitrogen and hereafter freeze dried. The freeze-dried samples were then analyzed by SEM.

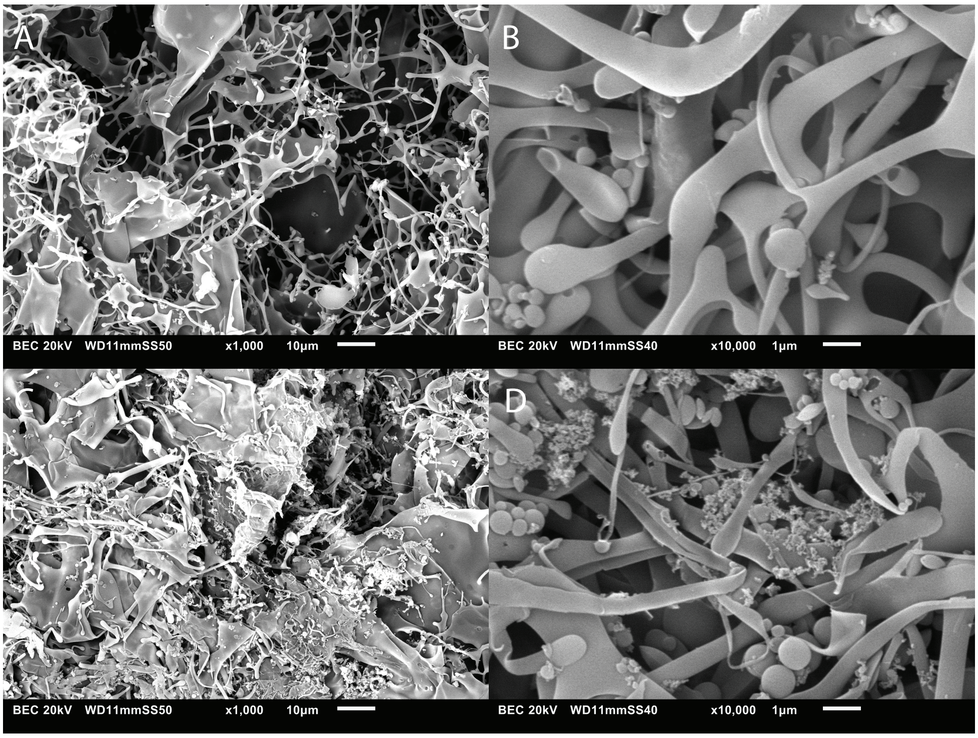

Supplementary Figure 11: A) and B) SEM images taken of alkyne-modified dextran hydrogels. C) and D) SEM images taken of ofloxacin/prodrug **9** modified dextran alkyne hydrogels.

S6.0 Signal triggered pro-drug activation/release studies with A549 cells

S.6.1 Cell culture and maintenance

A549 (lung epithelial adenocarcinoma) cell line was acquired by ATCC and cultured in Dulbecco's Modified Eagle Medium High Glucose (DMEM, Sigma) containing 4.5 g/L glucose, L-glutamine without sodium pyruvate, supplemented with 10% Fetal Bovine Serum (FBS, Sigma) and 1% Antibiotic- Antimycotic solution (Gibco). The cells were kept at 37℃ and 5% CO_2 in sterile conditions and sub-cultured at least twice a week (never exceeding passage number 20). Cell were frequently tested for mycoplasma absence.

S.6.2 Wound-closure assay

The wound healing assay was carried out in a μ-Slide 4 well chambered coverslip (ibidi). A cell suspension containing $5x10^4$ cells/mL, obtained via trypsinization, was pipetted in each

well to reach 70-80% confluence of the monolayer within 3 days. The cell-free gap was created on day 4 via direct manipulation: a 10 µL pipette tip was used to scratch the cell monolayer and generate the wound. Hydrogel, reactants and additives were added to each chamber, according to the tested conditions, immediately prior to the start of the experiment.

S.6.3 Wound-closure evaluation

To maintain favorable conditions for the cell monolayer, all experiments were conducted at 37℃ and 5% CO_2 using a stage top incubator (ibidi). Brightfield images were taken every hour for a total of 72 hours on an inverted microscope (Zeiss Axio-Observer Z1) equipped with an EMCCD camera (Andor ixon 3) with a resolution of 512 x 512 pixels and a moving stage, which allowed for the imaging of multiple conditions per experiment. Data analysis was performed with a machine-learning-based (bio)image analysis tool called ilastik (Heidelberg Collaboratory for Image Processing HCI) via Pixel and Object classification workflow. The software enabled for optimal binarization of low-contrast brightfield images with a small training dataset. The binarized image sequences were then processed in ImageJ (v1.53t, National Institute of Health, USA) for evaluation of the wound closure over time. The normalized wound closure (%) is defined in Eq.1 as follows:

$$\left(1-\frac{A(t)}{A_0}\right)\ x\ 100 \qquad \textbf{Eq.1}$$

Where A0 and A(t) are respectively the wound area at time 0, corresponding to the beginning of the experiment, and the wound area at time 1 to 72 (in hours).

S.6.4 Wound-closure evaluation – GSH only

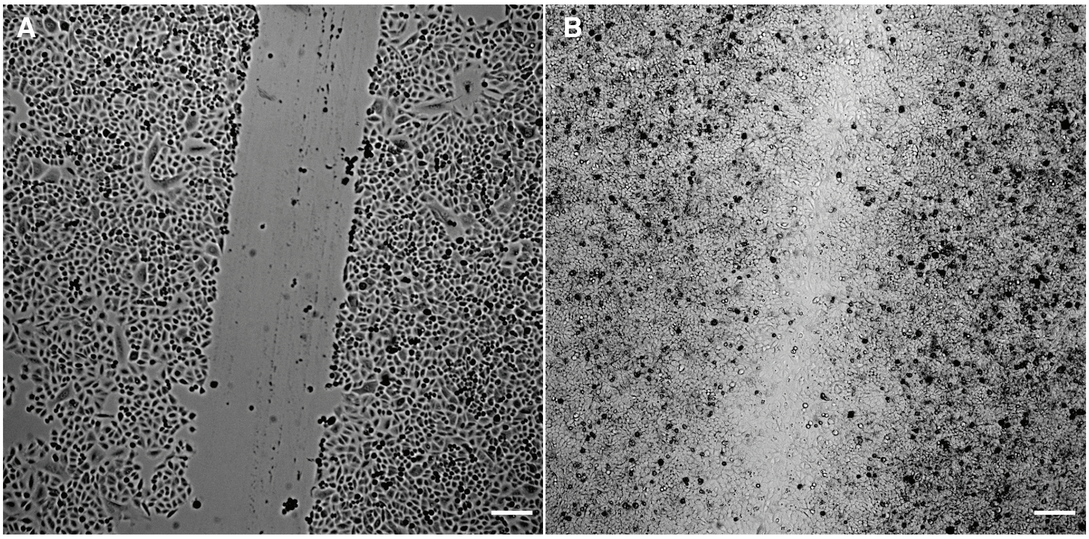

Supplementary Figure 12: Brightfield images taken of wound closure experiment with only hydrogel, no prodrug and 400 µM of **15** at (A) 0 hours and (B) 72 hours.

7.0 Synthesis of compounds

S7.1 Synthesis of 4-Azidobenzaldehyde

A mixture of 4-formylphenylboronic acid (33.0 mmol, 4.95 g, 1.0 eq.), sodium azide (89.2 mmol, 5.8 g, 2.7 eq.) and copper(II)acetate (3.3 mmol, 0.6 g, 0.1 eq.) are stirred for 24 hours in methanol (180 mL). After completion, the reaction mixture is concentrated on celite under reduced pressure and purified by silica column chromatography (5:5 petroleum ether:ethyl acetate) to yield the title compound as an yellow oil (25.1 mmol, 3.7 g, 76%).

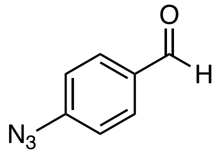

^{1}**H NMR** (400 MHz, $CDCl_3$) δ 9.86 (s, 1H), 7.80 (d, J = 8.5 Hz, 2H), 7.08 (d, J = 8.4 Hz, 2H). ^{13}**C NMR (**101 MHz, $CDCl_3$) δ: 190.6, 146.4, 133.3, 131.6, 131.6, 119.6. **IR (ATR)** (cm^{-1}): 2109, 1689, 1594, 1502, 1280, 1211, 1165, 1126, 827, 781.

S7.2 Synthesis of methyl 2-((4-azidophenyl)(hydroxy)methyl)acrylate

4-Azidobenzaldehyde (25.1 mmol, 3.7 g, 1.0 eq.), methyl acrylate (75.4 mmol, 6.8 mL, 3.0 eq.), triethanolamine (20.1 mmol, 3.0 g, 0.8 eq.) and DABCO (25.1 mmol, 2.8 g, 1.0 eq.) are added to a flask with 20 mL THF and stirred at RT for 4 days. After completion the reaction mixture is diluted with water and extracted three times with DCM. The organic layers are then dried with Na_2SO_4 and concentrated under reduced pressure. The residue is then purified by silica column chromatography (8:2 petroleum ether:ethyl acetate) to yield the title compound as pale yellow oil (8.1 mmol, 1.9 g, 32%).

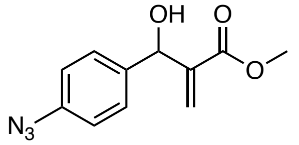

^{1}**H NMR** (400 MHz, $CDCl_3$) δ: 7.26 (d, J = 8.5 Hz, 2H), 6.90 (d, J = 8.2 Hz, 2H), 6.24 (s, 1H), 5.77 (s, 1H), 5.43 (s, 1H), 3.62 (s, 3H), 3.25 (s, 1H). ^{13}**C NMR (**101 MHz, $CDCl_3$) δ: 166.6, 141.8, 139.5, 138.1, 128.1, 126.0, 119.0, 72.5, 52.0. **IR (ATR)** (cm^{-1}): 3284, 2954, 2872, 2117, 1716, 1589, 1436, 1261, 1161, 1057, 829, 748.

S7.3 Synthesis of (Z) methyl 2-((4-azidophenyl)(hydroxy)methyl)acrylate (1)

Methyl 2-((4-azidophenyl)(hydroxy)methyl)acrylate (8.1 mmol, 1.9 g. 1.0 eq.) is dissolved in anhydrous DCM (40 mL) and cooled to 0 degrees Celsius. PBr_3 (1M DCM solution, 1.33 mL, 0.9 eq.) is then added dropwise under an argon atmosphere. After completion the reaction is stopped by adding ice. The mixture is then extracted with DCM and washed twice with water. The organic layers are then dried with Na_2SO_4, filtered and concentrated under reduced pressure. The residue is then purified by silica column chromatography (9.5:0.5 petroleum ether:ethyl acetate) to yield methyl 3-(4-azidophenyl)-2-(bromomethyl)acrylate as white-yellowish solid (1.9 g, 6.4 mmol, 80%).

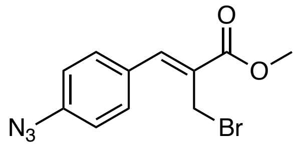

^{1}H NMR (400 MHz, $CDCl_3$) δ: 7.70 (s, 1H), 7.57 – 7.49 (m, 2H), 7.09 – 7.01 (m, 2H), 4.32 (s, 2H), 3.81 (s, 3H). **^{13}C NMR** (101 MHz, $CDCl_3$) δ: 166.6, 141.9, 141.6, 131.5, 130.9, 128.2, 119.5, 52.5, 26.7. **IR (ATR)** (cm^{-1}): 2112, 1711, 1601, 1502, 1433, 1266, 1215, 1188, 1153, 1080, 833, 768.

S7.4 Synthesis of (Z,E) 1-(3-(4-azidophenyl)-2-(methoxycarbonyl)allyl)-1,4-diazabicyclo[2.2.2]octan-1-ium bromide (13)

3-(4-azidophenyl)-2-(bromomethyl)acrylate (0.081 mmol, 23.9 mg, 1.0 eq.) and DABCO (0.083 mmol, 9.35 mg, 1.0 eq.) are dissolved in THF (1.0 mL) and stirred overnight. The resulting precipitate is washed with diethyl ether and collected to give the title compound as a yellowish solid (0.07 mmol, 28.3 mg, 86%).

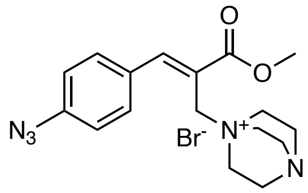

^{1}NMR (400 MHz, MeOD) (E - configuration, 67%) δ: 8.42 (s, 1H), 7.62 (d, J = 8.8 Hz, 2H), 7.23 (d, J = 8.6 Hz, 2H), 4.56 (s, 2H), 3.91 (s, 3H), 3.24 – 3.15 (m, 6H), 3.10 – 3.00 (m, 6H). (Z - configuration, 33%) δ: 7.52 (s, 1H), 7.50 (d, J = 4.6 Hz, 2H), 7.12 (d, J = 8.7 Hz, 2H), 4.36 (s, 2H), 3.75 (s, 3H), 3.51 – 3.39 (m, 6H), 3.27 – 3.16 (m, 6H). **^{13}C NMR** (101 MHz, MeOD) (E - configuration, 67%) δ: 168.4, 152.3, 143.8, 132.4, 131.7, 121.2, 121.0, 59.1, 53.6, 53.6, 46.2. (Z - configuration, 33%) δ: 168.8, 151.0, 143.4, 132.4, 132.1, 120.9, 119.9, 68.5, 53.7, 53.0, 46.2. **MS** (ESI+) m/z: 328.13 (M-Br) (expected m/z: 328.18).

S7.5 Synthesis of 1-(3-(4-azidophenyl)-2-(methoxycarbonyl)allyl)-1,4-lidocaine bromide (8)

3-(4-azidophenyl)-2-(bromomethyl)acrylate (0.143 mmol, 42.2 mg, 1.0 eq.) and lidocaine (0.129 mmol, 30.3 mg, 0.9 eq.) are dissolved in CH_3CN (1.0 mL) and stirred overnight. The residue is then purified by silica column chromatography (20:1 DCM:MeOH) to yield the title compound as an orange-yellow oil (45.9 mg, 0.087 mmol, 67%).

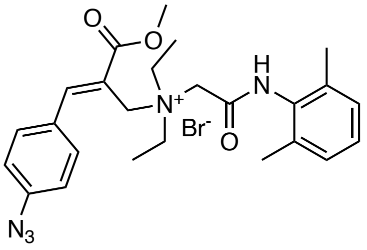

^{1}NMR (400 MHz, MeOD) δ: 8.41 (s, 1H), 7.56 (d, J = 8.5 Hz, 2H), 7.21 (d, J = 8.6 Hz, 2H), 7.12 (q, J = 5.4 Hz, 3H), 5.01 (s, 2H), 4.28 (s, 2H), 3.92 (s, 3H), 3.47 (qd, J = 13.4, 7.0 Hz, 4H), 2.22 (s, 6H), 1.20 (t, J = 7.1 Hz, 6H).). **^{13}C NMR** (101 MHz, MeOD) δ: 167.3, 162.4, 151.3, 142.2, 135.2, 132.6, 130.6, 130.3, 127.9, 127.5, 121.4, 119.6, 56.1, 55.3, 53.2, 52.2, 17.2, 7.3. **MS** (ESI+) m/z: 450.07 (M-Br) (expected m/z: 450.25).

S7.6 Synthesis of 1-(3-(4-azidophenyl)-2-(methoxycarbonyl)allyl)-1,4-aripiprazole bromide (11)

3-(4-azidophenyl)-2-(bromomethyl)acrylate (0.173 mmol, 51.2 mg, 1.0 eq.) and aripiprazole (0.158 mmol, 70.8 mg, 0.9 eq.) are dissolved in THF (1.0 mL) and stirred for 5 days. The resulting precipitate is washed with diethyl ether and collected to give the title compound as a pale-yellow solid (0.0645 mmol, 48 mg, 41%).

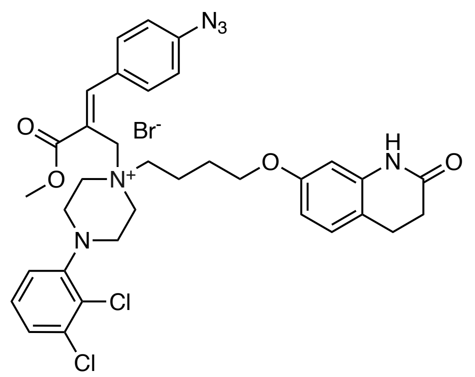

[1]NMR (400 MHz, DMSO) δ: 10.02 (s, 1H), 8.34 (s, 1H), 7.70 (d, J = 8.4 Hz, 2H), 7.43 – 7.32 (m, 2H), 7.25 (d, J = 8.4 Hz, 2H), 7.14 (dd, J = 7.6, 2.0 Hz, 1H), 7.06 (d, J = 8.3 Hz, 1H), 6.47 (dd, J = 8.2, 2.5 Hz, 1H), 6.43 (d, J = 2.5 Hz, 1H), 4.71 (s, 2H), 3.86 (s, 3H), 3.75 (t, J = 6.2 Hz, 2H), 3.49 – 3.36 (m, 6H), 3.20 (d, J = 13.4 Hz, 2H), 2.79 (t, J = 7.5 Hz, 2H), 2.41 (dd, J = 8.5, 6.5 Hz, 2H), 1.54 (t, J = 5.6 Hz, 2H), 1.40 (t, J = 7.2 Hz, 2H). **^{13}C NMR** (101 MHz, DMSO) δ: 170.7, 167.5, 158.1, 151.0, 149.4, 141.9, 139.7, 133.1, 131.8, 130.5, 128.9 (d, J = 5.3 Hz), 126.6, 126.0, 120.7 (d, J = 9.2 Hz), 120.3, 116.2, 107.8, 102.2, 67.3, 58.2, 53.5, 44.6, 31.2, 25.9, 24.4, 19.0. **MS** (ESI+) m/z: 663.15 (M-Br) (expected m/z: 663.22).

S7.7 Synthesis of 1-(3-(4-azidophenyl)-2-(methoxycarbonyl)allyl)-1,4-ofloxacin bromide (9)

3-(4-azidophenyl)-2-(bromomethyl)acrylate (0.175 mmol, 51.8 mg, 1.0 eq.) and ofloxacin (0.166 mmol, 60.0 mg, 0.95 eq.) are dissolved in CH_3CN (2.5 mL) and stirred for 48 hours. The resulting precipitate is washed with ethyl acetate and then diethyl ether and collected to give the title compound as a pale-yellow solid (0.083 mmol, 54.4 mg, 50%).

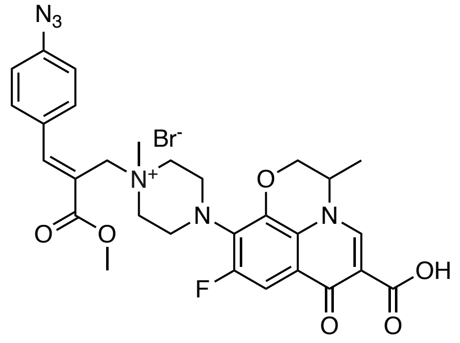

[1]NMR (400 MHz, DMSO) δ: 8.99 (s, 1H), 8.35 (s, 1H), 7.73 (d, J = 8.2 Hz, 2H), 7.60 (d, J = 12.0 Hz, 1H), 7.26 (d, J = 8.6 Hz, 2H), 4.94 (d, J = 7.1 Hz, 1H), 4.72 (s, 2H), 4.55 (d, J = 12.1 Hz, 1H), 4.39 (d, J = 11.8 Hz, 1H), 3.86 (s, 3H), 3.69 – 3.34 (m, 9H), 2.95 (s, 3H), 1.45 (d, J = 6.7 Hz, 3H). **^{13}C NMR** (101 MHz, DMSO) δ: 176.8 (d, J = 3.3 Hz), 167.5, 166.3, 156.7, 154.3, 151.0, 146.9, 141.7, 140.8 (d, J = 6.5 Hz), 132.0, 130.6, 130.5, 130.3, 125.1, 120.9 (d, J = 9.4 Hz), 120.4, 120.3, 119.6, 107.3, 103.7 (d, J = 23.9 Hz), 68.8, 67.5, 60.4, 58.5, 55.3, 53.4, 45.8, 44.1, 25.6, 18.4. **^{19}F NMR** (376 MHz, DMSO) δ: -120.24 (d, J = 12.1 Hz). **MS** (ESI+) m/z: 577.13 (M-Br) (expected m/z: 577.22).

S7.8 Synthesis of 1-(3-(4-azidophenyl)-2-(methoxycarbonyl)allyl)-1,4-atropine bromide (10)

3-(4-azidophenyl)-2-(bromomethyl)acrylate (0.169 mmol, 50.2 mg, 1.0 eq.) and atropine (0.141 mmol, 40.8 mg, 0.83 eq.) are dissolved in THF (1.5 mL) and stirred overnight. The residue is then purified by silica column chromatography (90:10 DCM:MeOH) to yield the title compound as an white-yellowish oil (54.1 mg, 0.092 mmol, 66%).

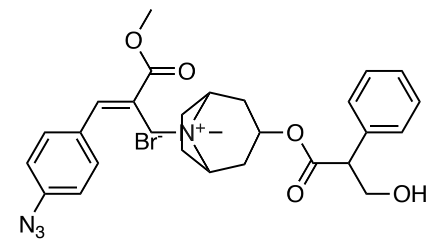

^{1}NMR (400 MHz, MeOD) δ: 8.40 (s, 1H), 7.52 (d, J = 8.2 Hz, 2H), 7.41 – 7.29 (m, 5H), 7.24 (d, J = 8.5 Hz, 2H), 5.04 (t, J = 5.8 Hz, 1H), 4.48 (s, 2H), 4.23 – 4.09 (m, 1H), 3.92 (s, 3H), 3.87 – 3.76 (m, 4H), 3.67 (s, 1H), 2.79 (s, 3H), 2.63 – 2.39 (m, 2H), 2.25 – 2.12 (m, 2H), 1.97 (d, J = 17.0 Hz, 2H), 1.79 – 1.58 (m, 2H). **^{13}C NMR** (101 MHz, MeOD) δ: 171.3, 167.3, 150.9, 142.2, 135.7, 130.5, 130.3, 128.6, 128.5, 127.8, 127.5, 121.8, 119.7, 67.4, 67.1, 63.1 (d, J = 13.8 Hz), 54.6, 54.4, 52.1, 39.4, 31.9 (d, J = 3.4 Hz), 24.2, 23.9. **MS** (ESI+) m/z: 505.20 (M-Br) (expected m/z: 505.24).

S7.9 Synthesis of 1-(3-(4-azidophenyl)-2-(methoxycarbonyl)allyl)-1,4-gefitinib bromide (12)

3-(4-azidophenyl)-2-(bromomethyl)acrylate (0.67 mmol, 200 mg, 1.0 eq.) and gefitinib (0.2 mmol, 90 mg, 0.3 eq.) are dissolved in THF (1.5 mL) and stirred overnight. The resulting precipitate is washed with diethyl ether and collected to give the title compound as a light-greenish solid (47 mg, 0.063 mmol, 31 %).

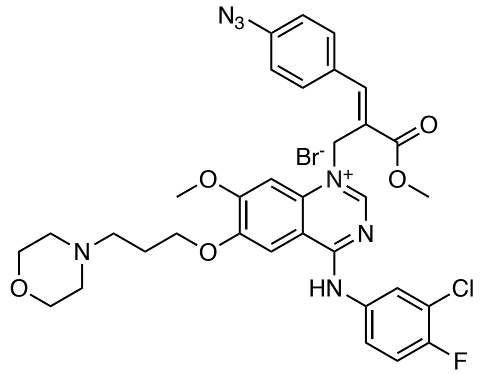

^{1}NMR (400 MHz, DMSO) δ: 11.46 (s, 1H), 9.83 (s, 1H), 8.95 (s, 1H), 8.33 (s, 1H), 8.07 (s, 1H), 7.95 (d, J = 6.9 Hz, 1H), 7.68 (t, J = 5.9 Hz, 1H), 7.56 (dq, J = 8.8, 5.8, 4.5 Hz, 3H), 7.18 (dd, J = 8.5, 2.3 Hz, 2H), 6.85 (s, 1H), 5.64 (s, 2H), 4.33 (s, 2H), 3.98 (d, J = 13.2 Hz, 2H), 3.75 – 3.64 (m, 7H), 3.54 (dd, J = 29.5, 9.8 Hz, 3H), 3.33 (s, 1H), 3.12 (d, J = 10.0 Hz, 2H), 2.28 (d, J = 11.1 Hz, 2H). **^{13}C NMR** (101 MHz, DMSO) δ: 166.41, 158.21, 157.03, 154.69, 152.53, 149.40, 145.01, 141.92, 135.83, 133.93, 132.22, 130.30, 129.93, 129.47, 127.58, 126.30 (d, J = 7.7 Hz), 123.78, 120.05, 119.79 (d, J = 18.8 Hz), 117.50 (d, J = 22.1 Hz), 107.82, 106.44, 99.03, 67.93, 67.45, 63.79, 57.05, 54.11, 52.95, 51.71, 49.22, 25.56, 23.33. **^{19}F NMR** (376 MHz, DMSO) δ: -118.24. **MS** (ESI+) m/z: 663.33 (M+H-Br) (expected m/z: 662.23) and m/z: 1347.45 (2M+Na-Br) (expected m/z: 1347.35).

S7.10 Synthesis of N-acetyl-S-(1-(4-azidophenyl)-2(methoxycarbonyl)allyl) cysteine (19)

(Z,E) 1-(3-(4-azidophenyl)-2-(methoxycarbonyl)allyl)-1,4-diazabicyclo [2.2.2] octan-1-ium bromide (4**a**) (0.091 mmol, 37.4 mg, 1.0 eq.) and N-acetyl cysteine (0.086 mmol, 14.1 mg, 0.94 eq.) are dissolved in H_2O (1.0 mL) and stirred for 2 hours. The resulting mixture is extracted with ethyl acetate and then evaporated to give the title compound as a bright-yellow oil (22.7 mg, 0.06 mmol, 69%).

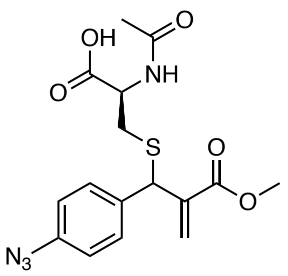

^{1}**NMR** (400 MHz, DMSO) δ: 12.78 (s, 1H), 8.23 (d, J = 9.2 Hz, 1H), 7.35 – 7.25 (m, 2H), 7.10 – 6.93 (m, 2H), 6.39 (d, J = 14.8 Hz, 1H), 6.09 (d, J = 17.2 Hz, 1H), 5.09 (d, J = 8.7 Hz, 1H), 4.43 – 4.27 (m, 1H), 3.59 (s, 3H), 2.80 – 2.58 (m, 2H), 1.82 (s, 3H). ^{13}**C NMR** (101 MHz, DMSO) δ: 171.99, 169.31, 165.57 (d, J = 7.1 Hz), 139.54, 139.12, 138.45, 136.32 (d, J = 5.3 Hz), 129.70, 127.36 (d, J = 19.1 Hz), 119.19 (d, J = 2.1 Hz), 52.09, 51.89, 51.48, 48.35 (d, J = 27.7 Hz), 33.33 (d, J = 17.6 Hz), 22.33. **MS** (ESI+) m/z: 378.78 (M+H) (expected m/z: 378.10).

S7.0 NMR Spectra

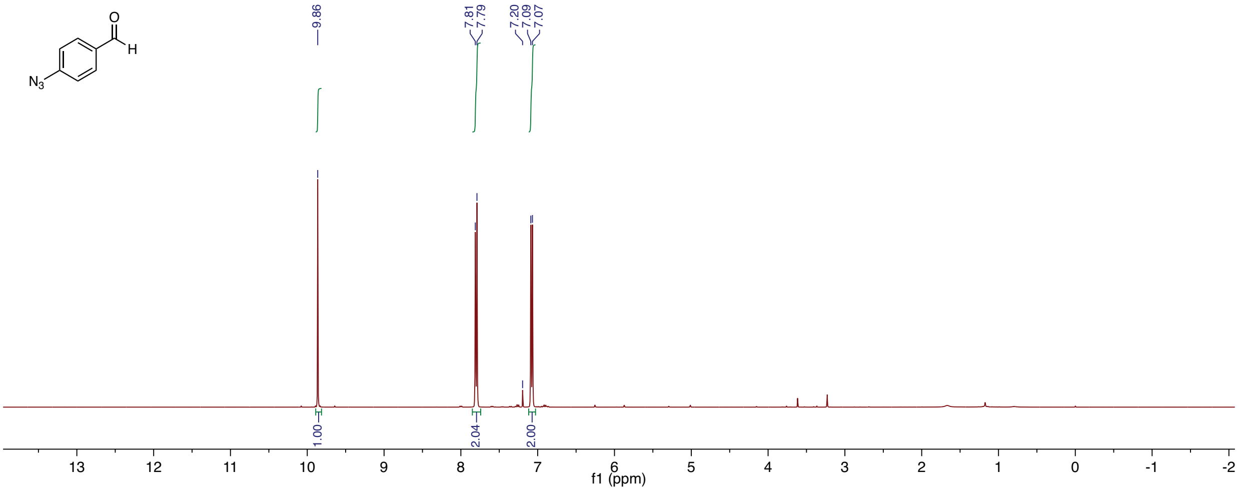

Supplementary Figure 13: ^{1}H NMR, 4-azidobenzaldehyde in $CDCl_3$.

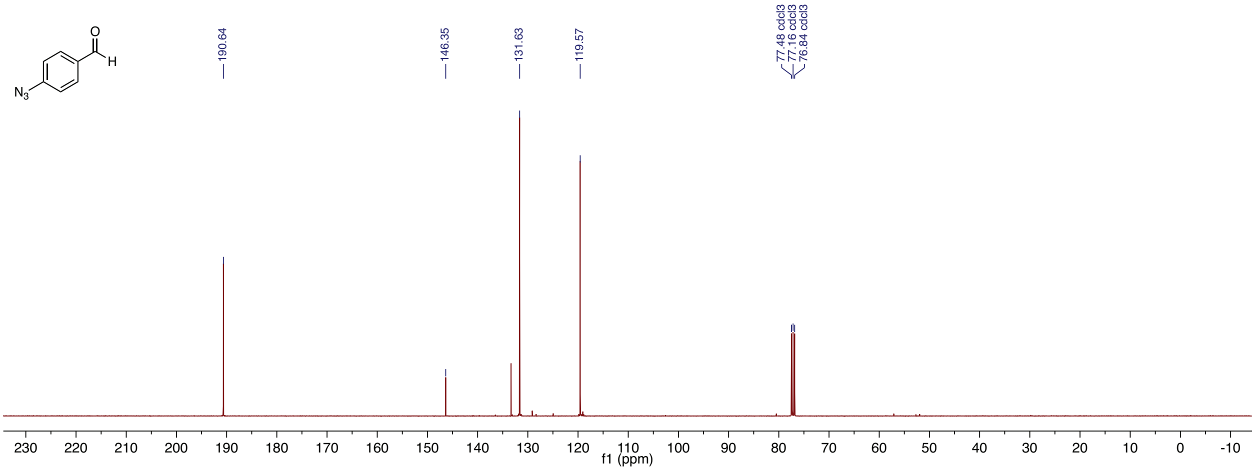

Supplementary Figure 14: ^{13}C NMR, 4-azidobenzaldehyde in $CDCl_3$.

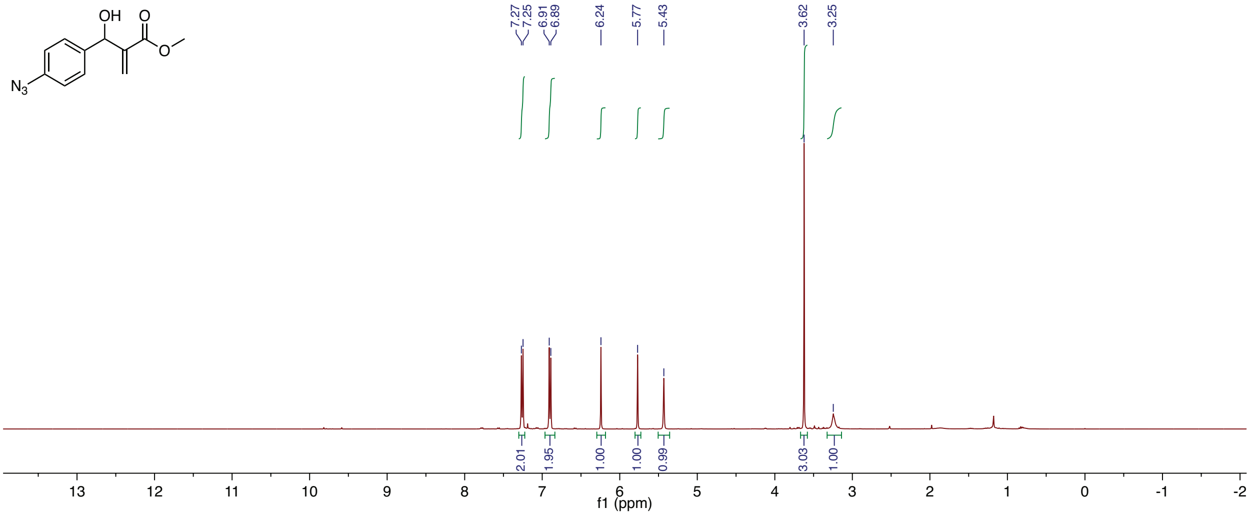

Supplementary Figure 15: ^{1}H NMR, methyl 2-((4-azidophenyl)(hydroxy)methyl)acrylate in $CDCl_3$.

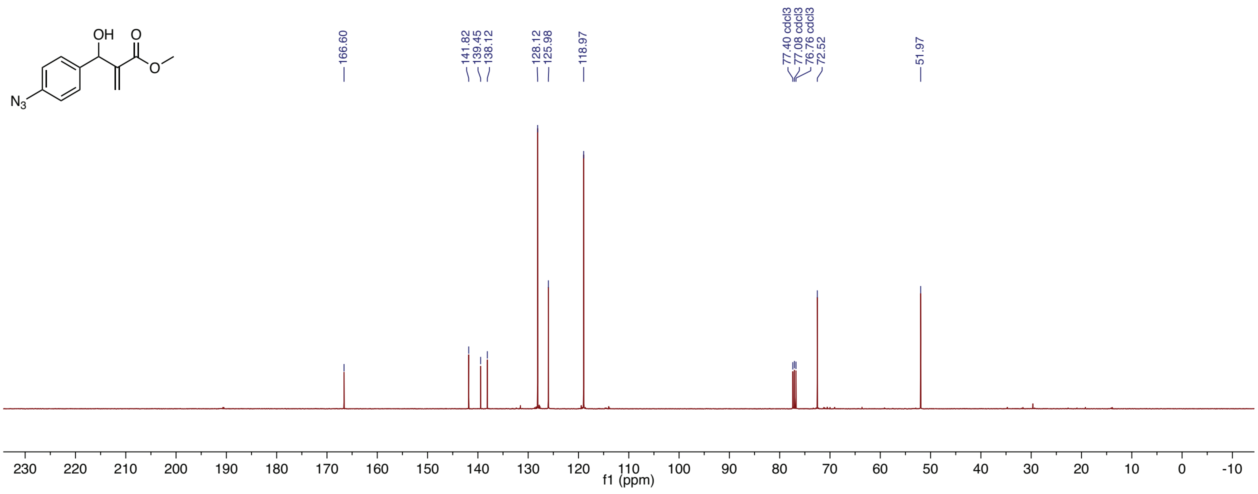

Supplementary Figure 16: ^{13}C NMR, methyl 2-((4-azidophenyl)(hydroxy)methyl)acrylate in $CDCl_3$.

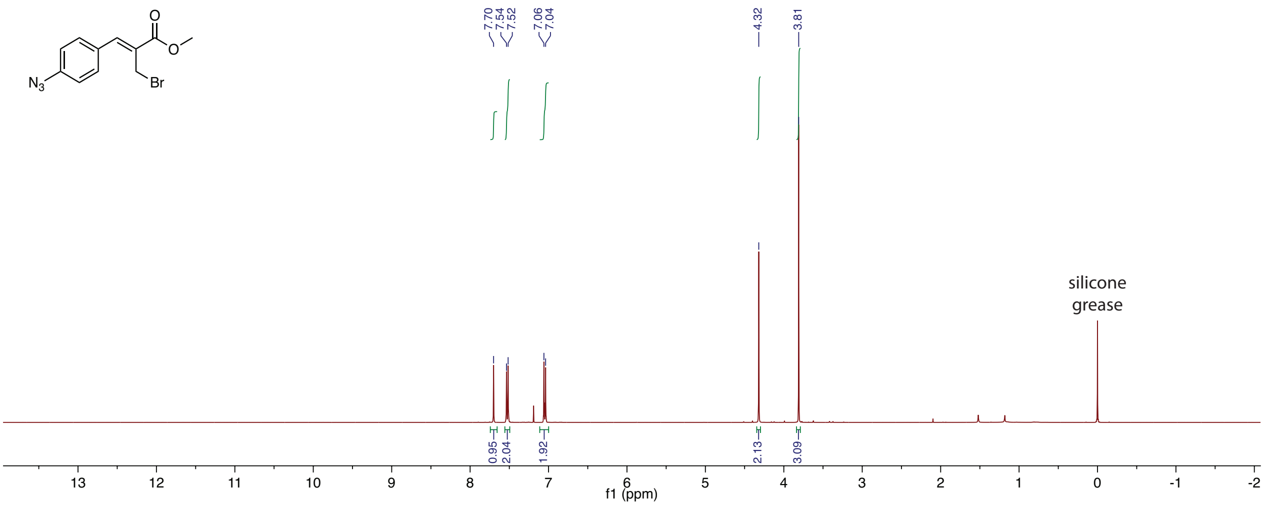

Supplementary Figure 17: ^{1}H NMR, compound **1** in $CDCl_3$.

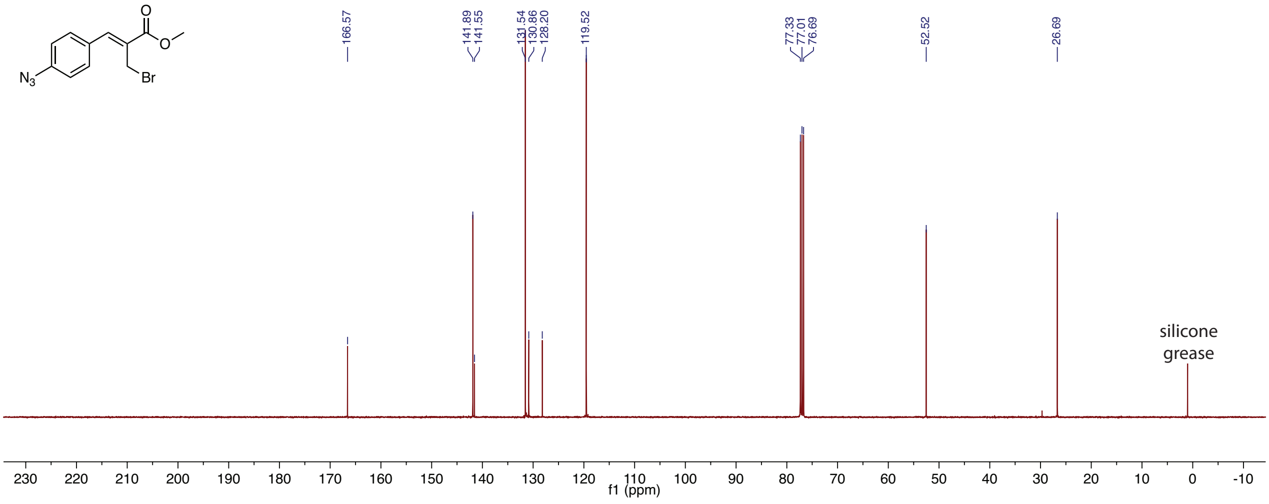

Supplementary Figure 18: ^{13}C NMR, compound **1** in $CDCl_3$.

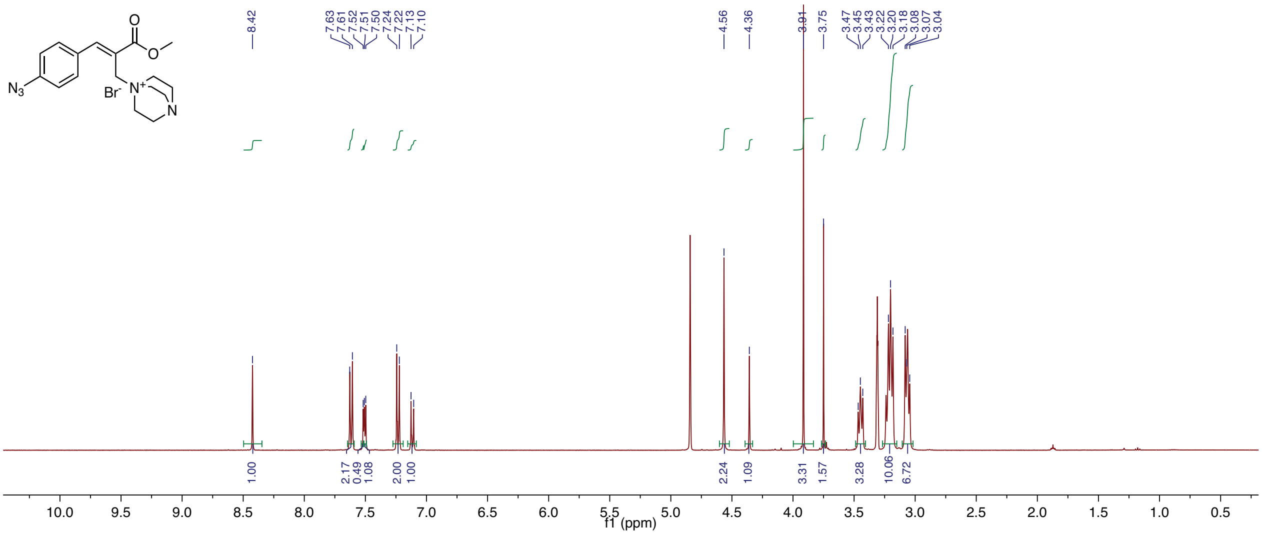

Supplementary Figure 19: ^{1}H NMR, compound **13** in MeOD.

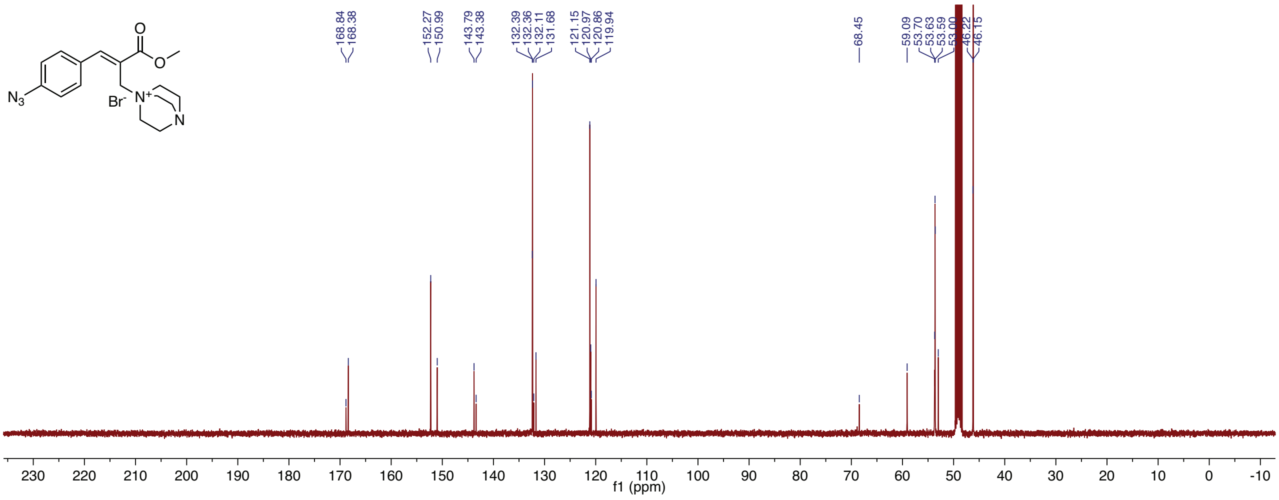

Supplementary Figure 20: ^{13}C NMR, compound **13** in MeOD.

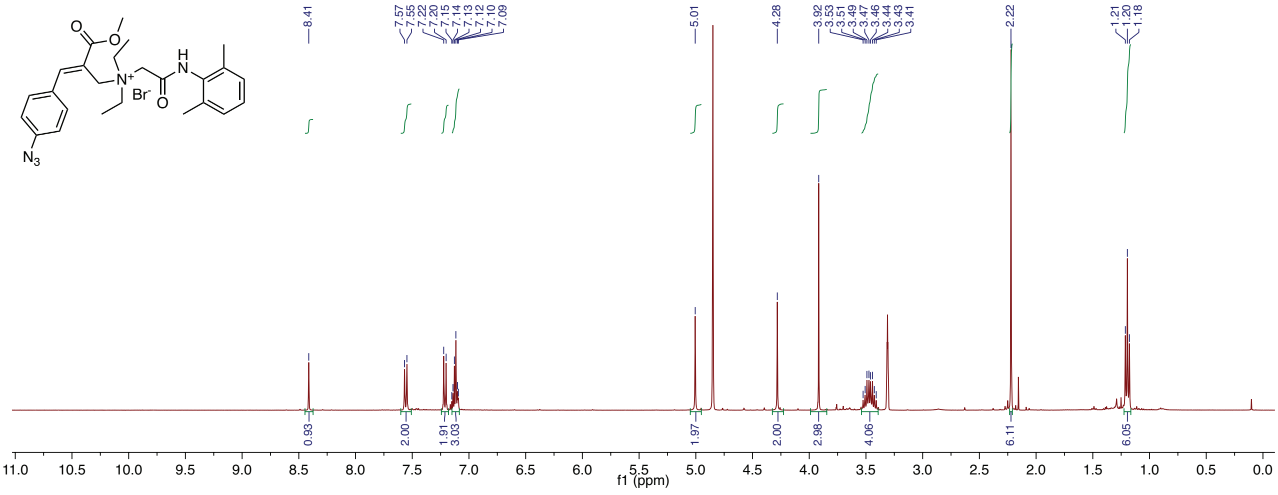

Supplementary Figure 21: ^{1}H NMR, compound **8** in MeOD.

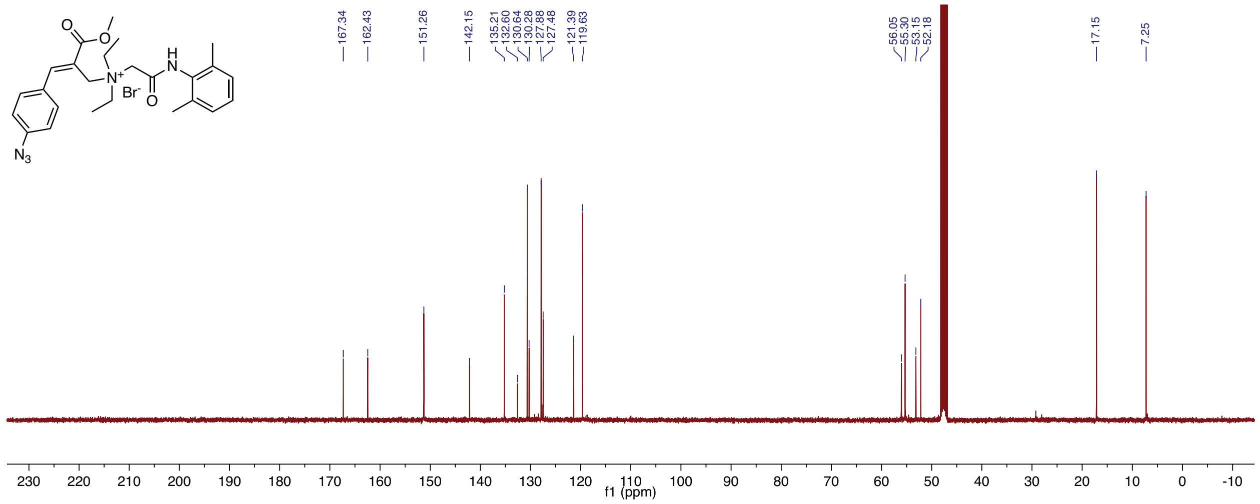

Supplementary Figure 22: ^{13}C NMR, compound **8** in MeOD.

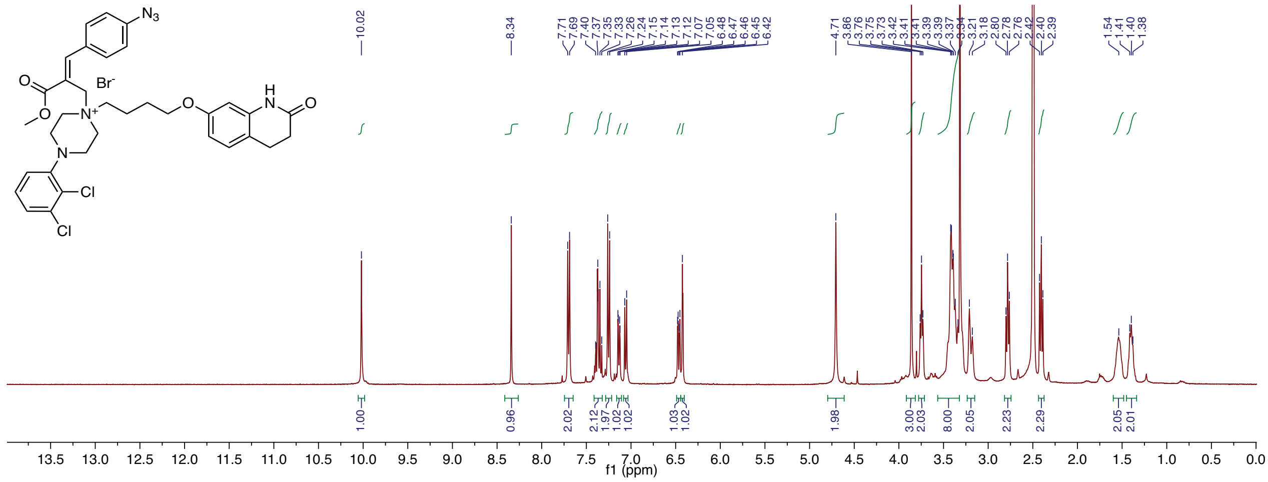

Supplementary Figure 23: ^{1}H NMR, compound **11** in d_6-DMSO.

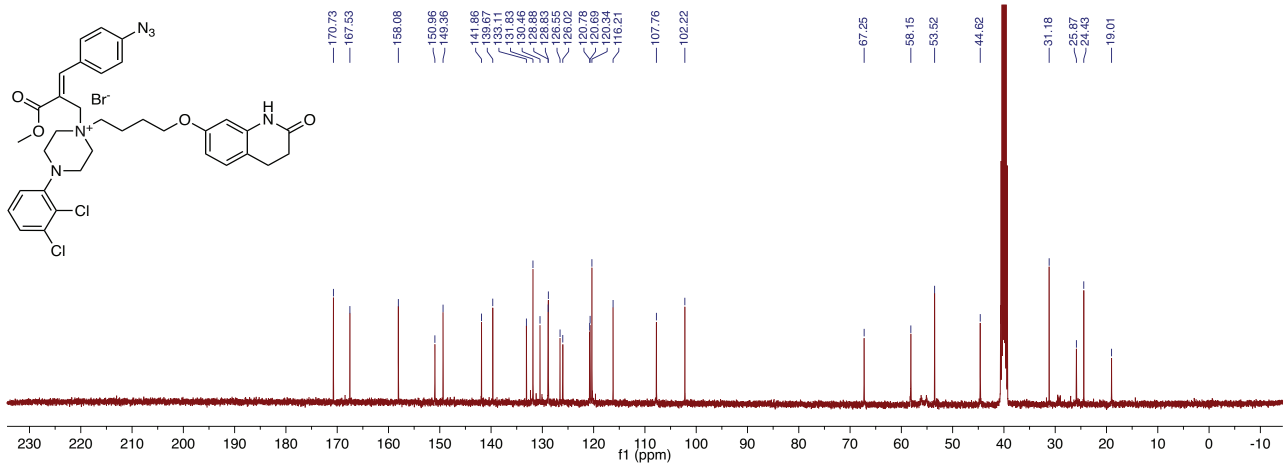

Supplementary Figure 24: ^{13}C NMR, compound **11** in d_6-DMSO.

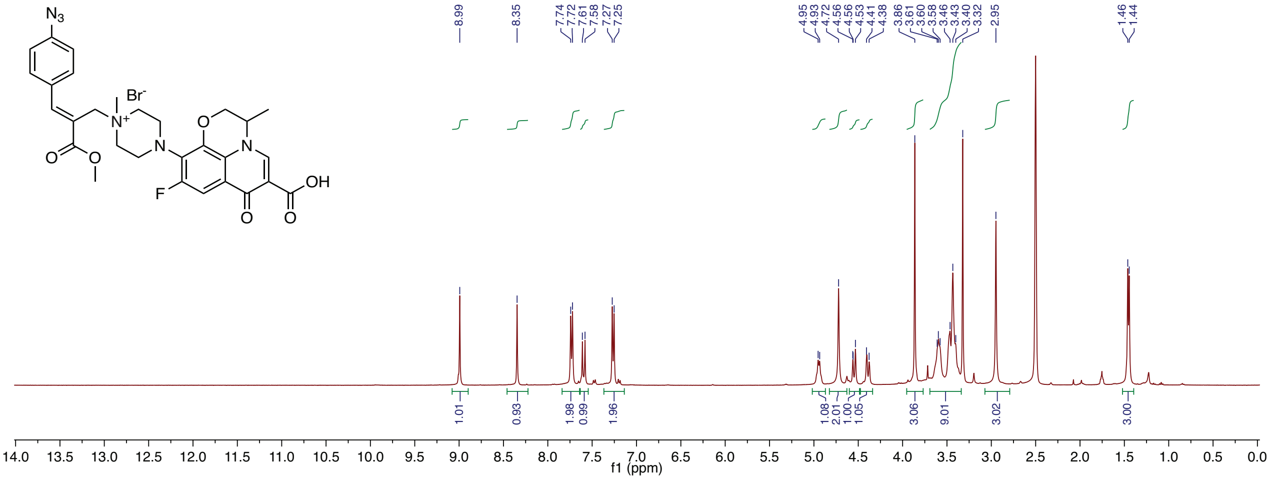

Supplementary Figure 25: ^{1}H NMR, compound **9** in d_6-DMSO.

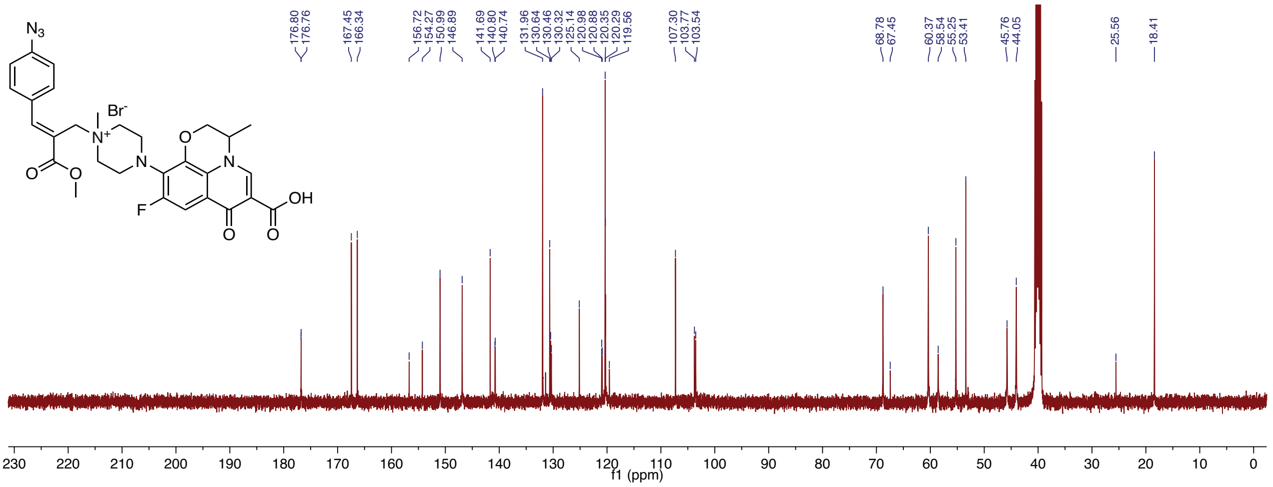

Supplementary Figure 26: ^{13}C NMR, compound **9** in d_6-DMSO.

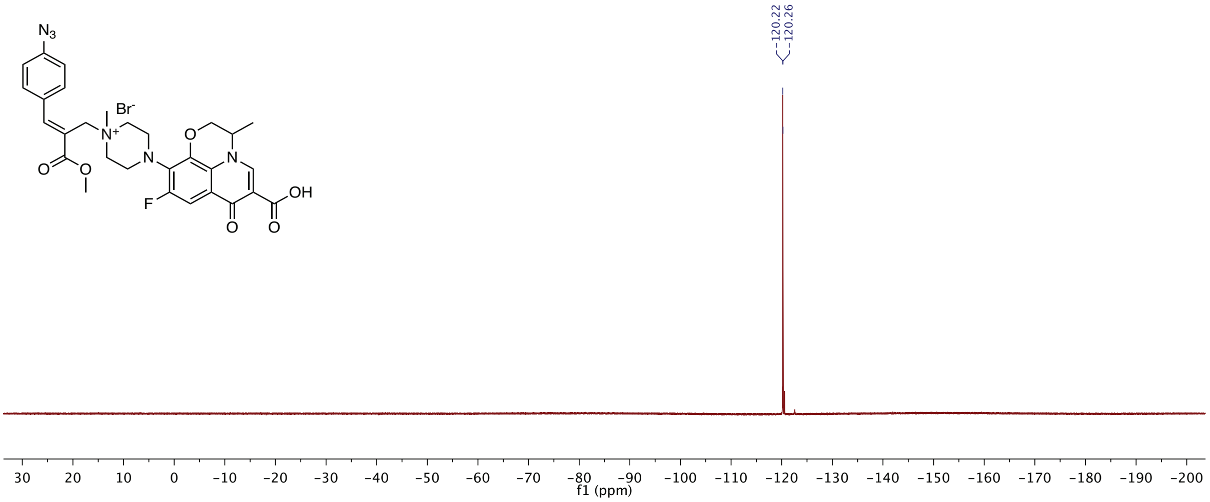

Supplementary Figure 27: ^{19}F NMR, compound **9** in d_6-DMSO.

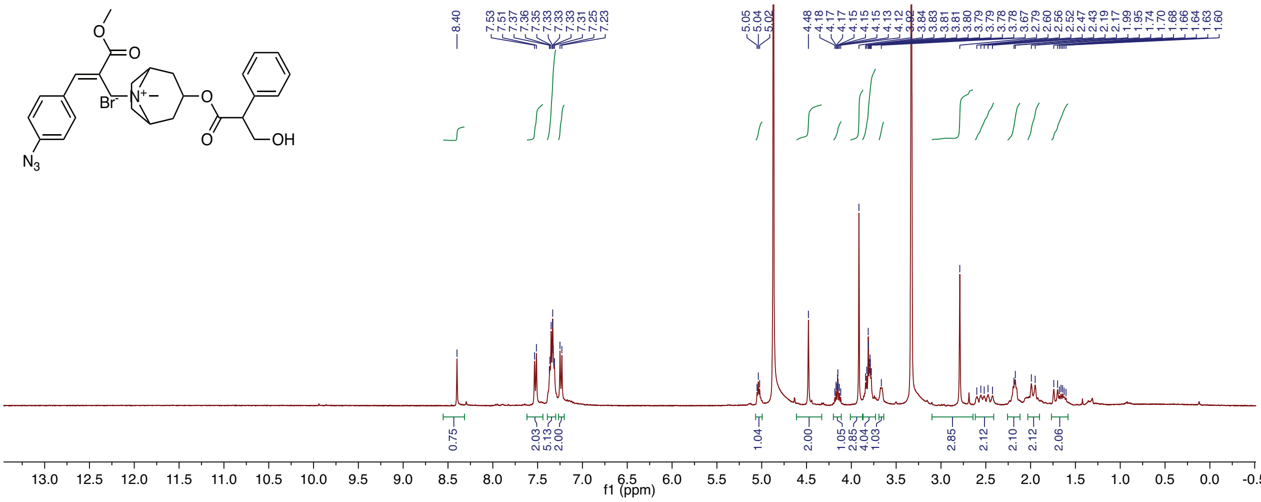

Supplementary Figure 28: ^{1}H NMR, compound **10** in MeOD.

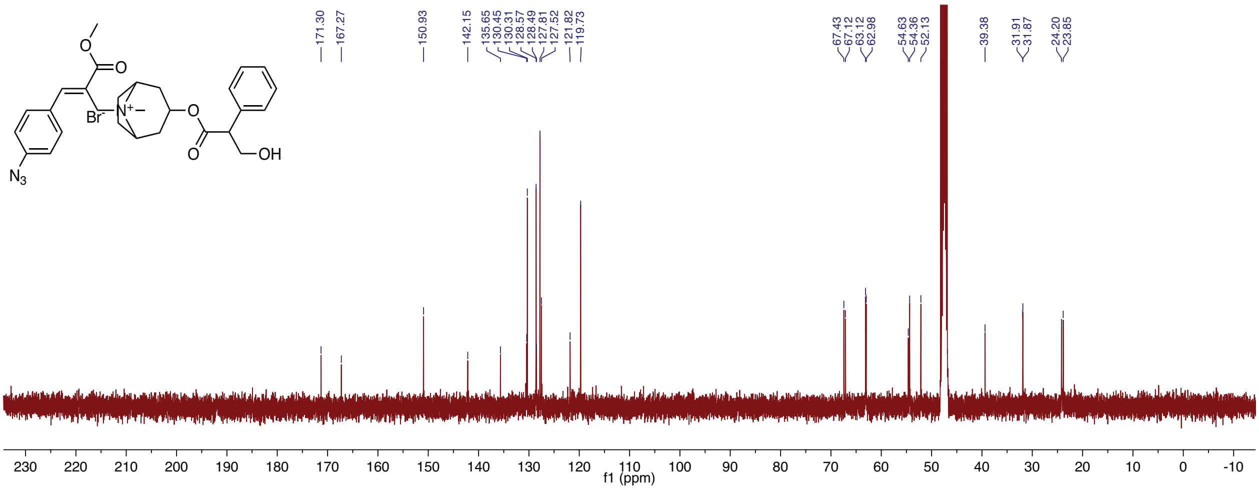

Supplementary Figure 29: ^{13}C NMR, compound **10** in MeOD.

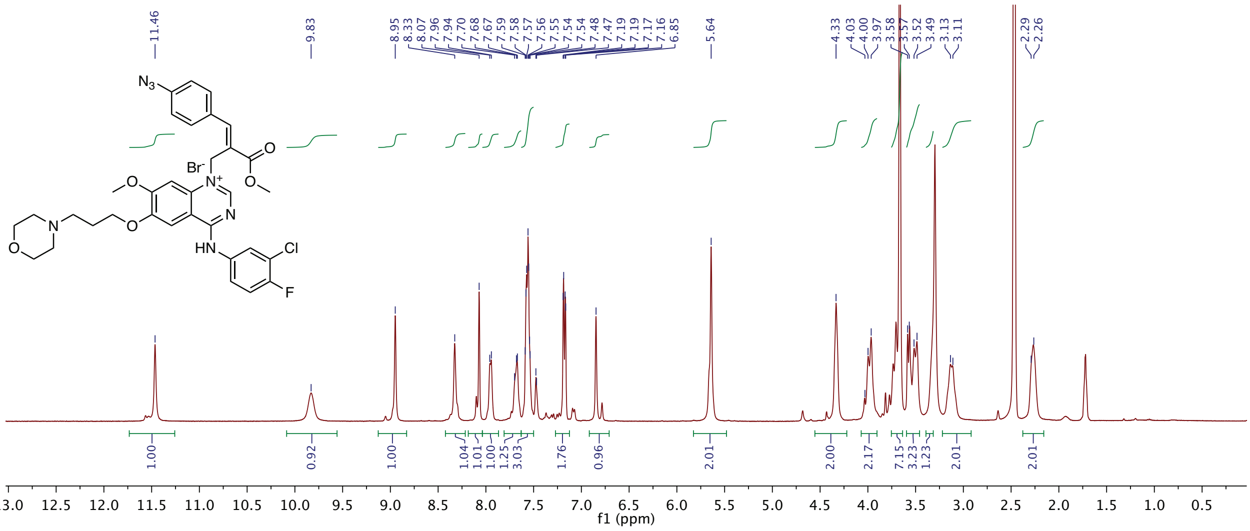

Supplementary Figure 30: ^{1}H NMR, compound **12** in d_6-DMSO.

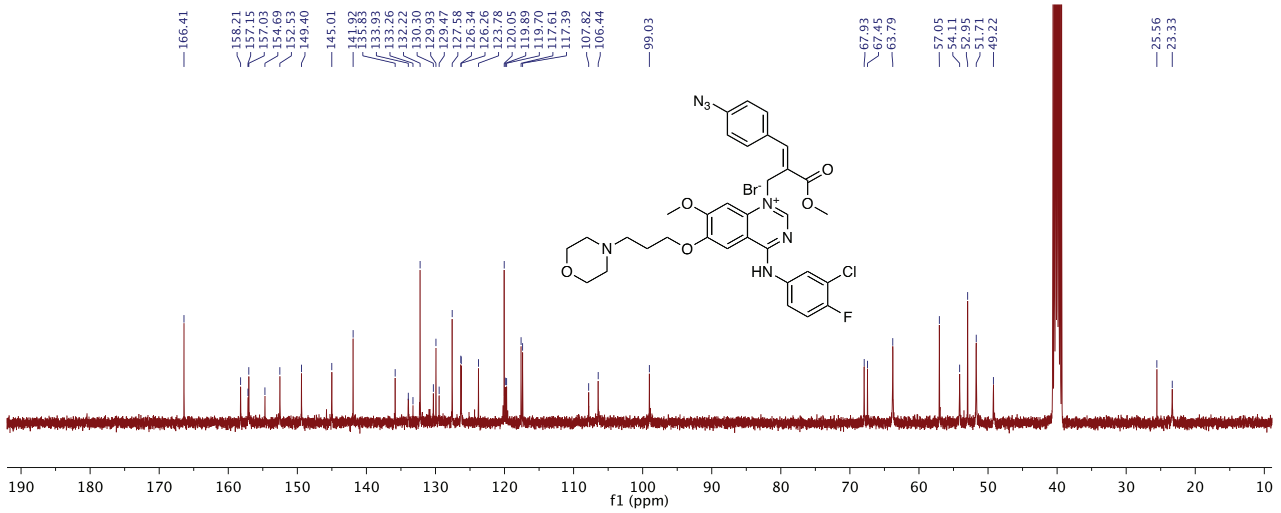

Supplementary Figure 31: ^{13}C NMR, compound **12** in d_6-DMSO.

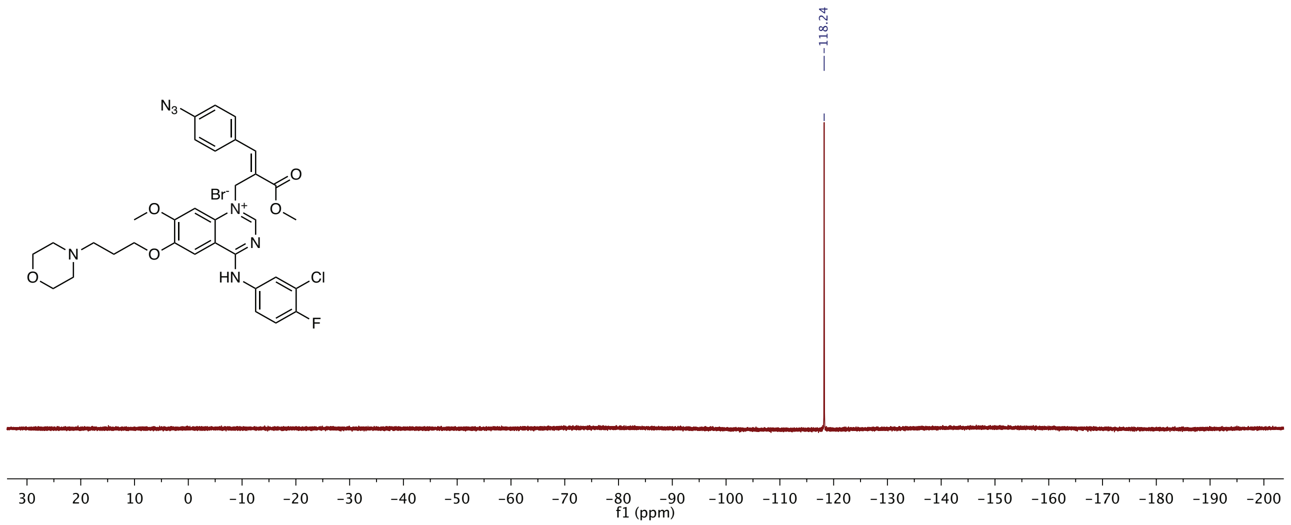

Supplementary Figure 32: ^{19}F NMR, compound **12** in d_6-DMSO.

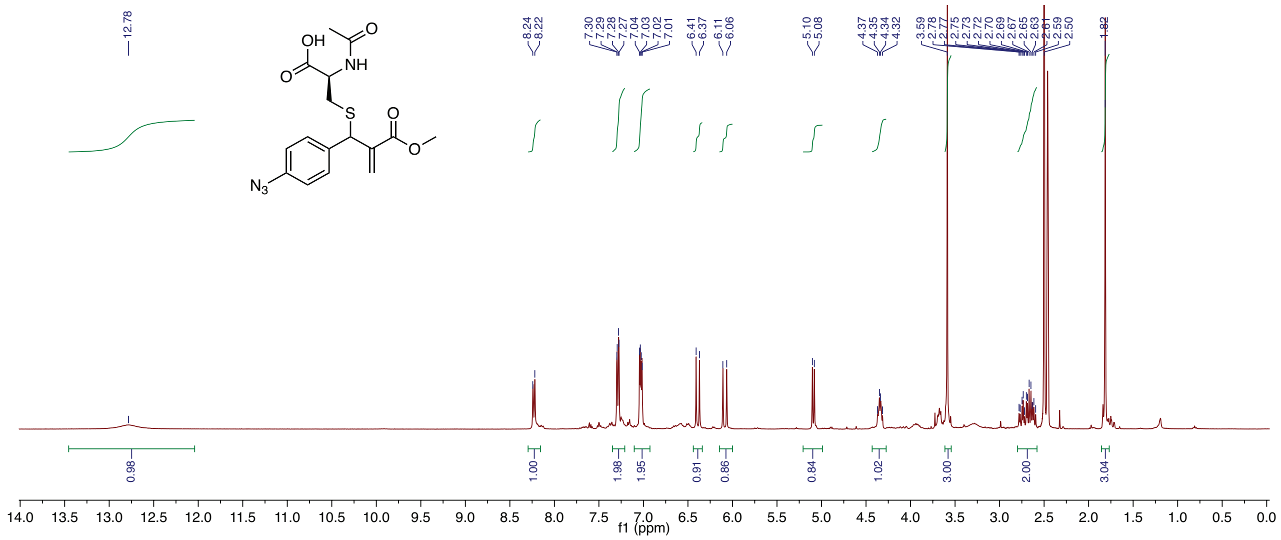

Supplementary Figure 33: ^{1}H NMR, compound **19** in d_6-DMSO.

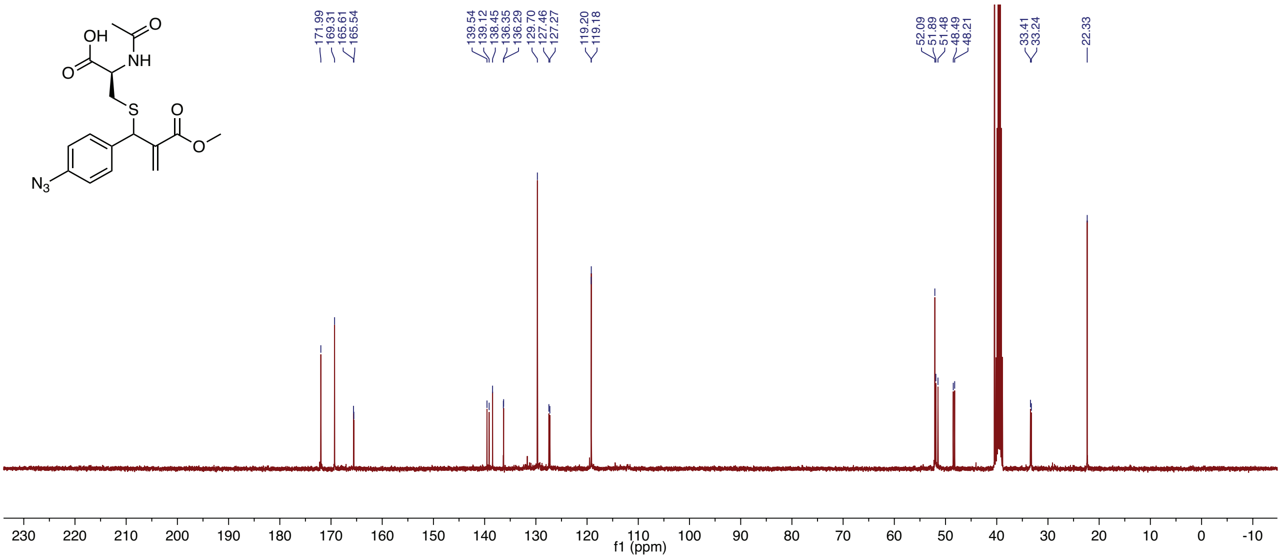

Supplementary Figure 34: ^{13}C NMR, compound **19** in d_6-DMSO.

S8.0 2-D NMR Spectra

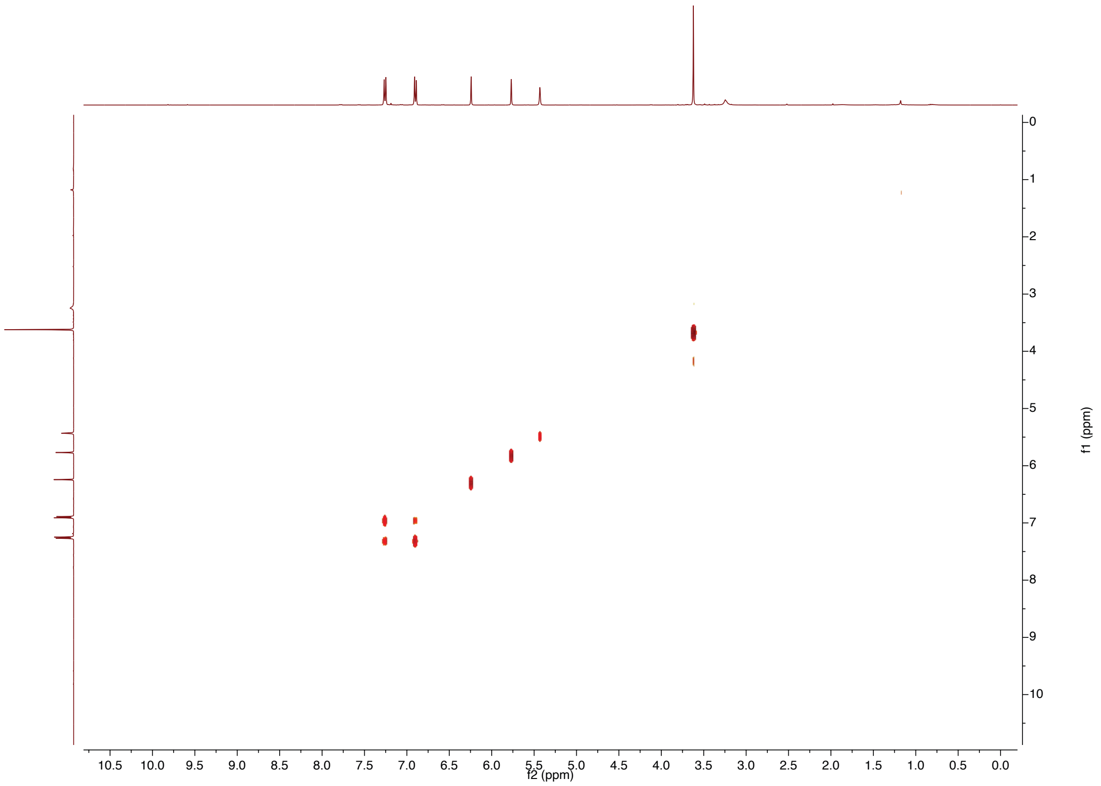

Supplementary Figure 35: gCOSY of methyl 2-((4-azidophenyl)(hydroxy)methyl)acrylate in $CDCl_3$.

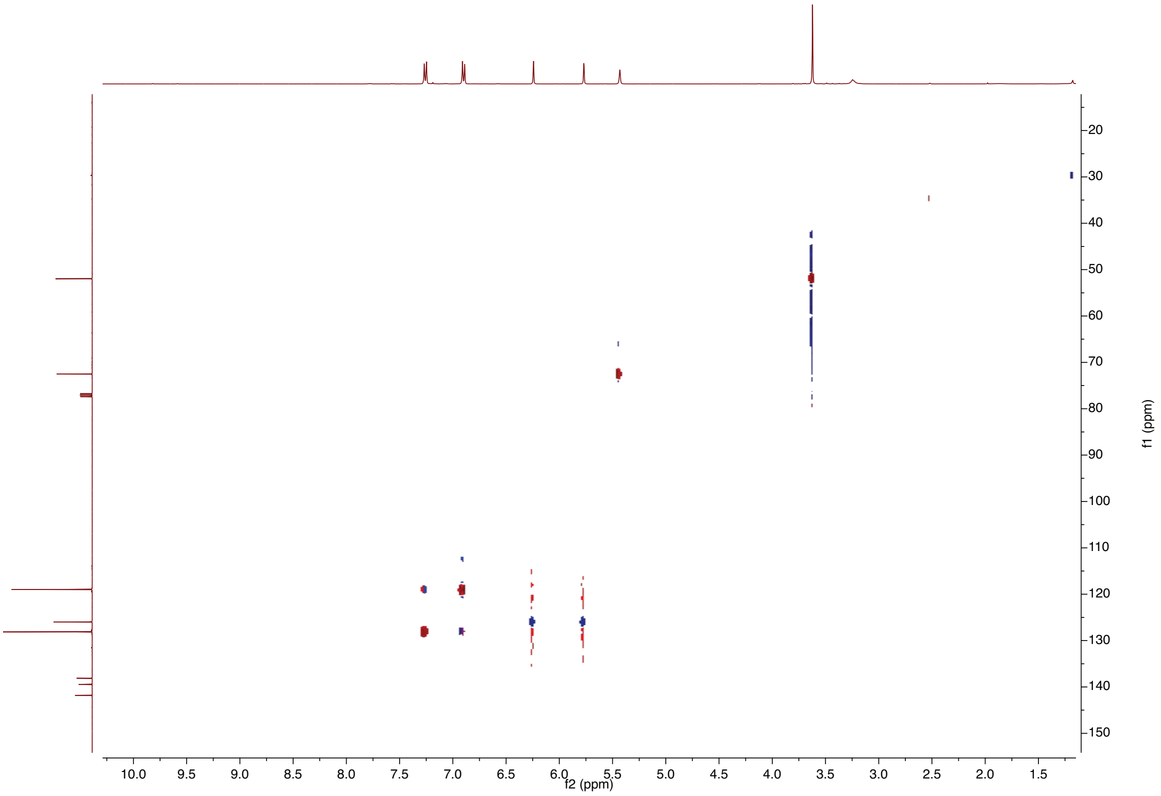

Supplementary Figure 36: gHSQC of methyl 2-((4-azidophenyl)(hydroxy)methyl)acrylate in $CDCl_3$.

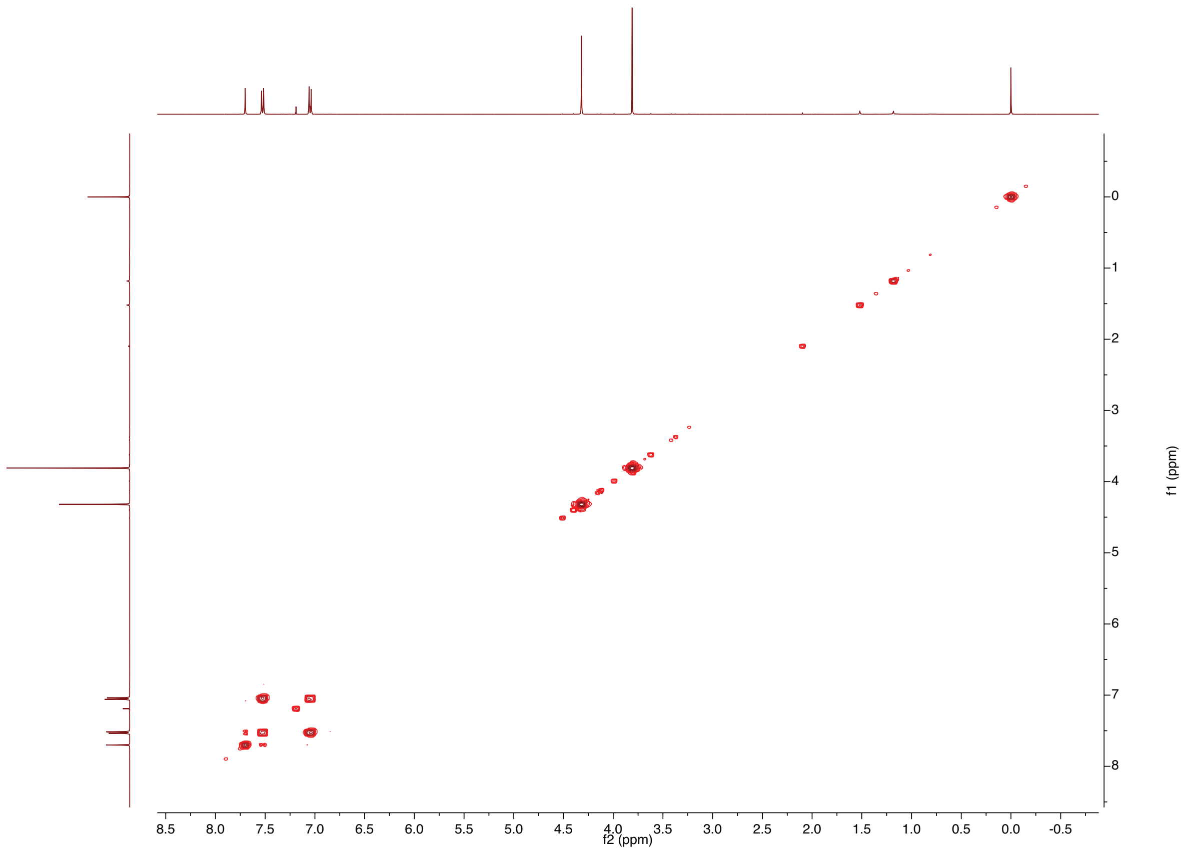

Supplementary Figure 37: gCOSY of compound **1** in $CDCl_3$.

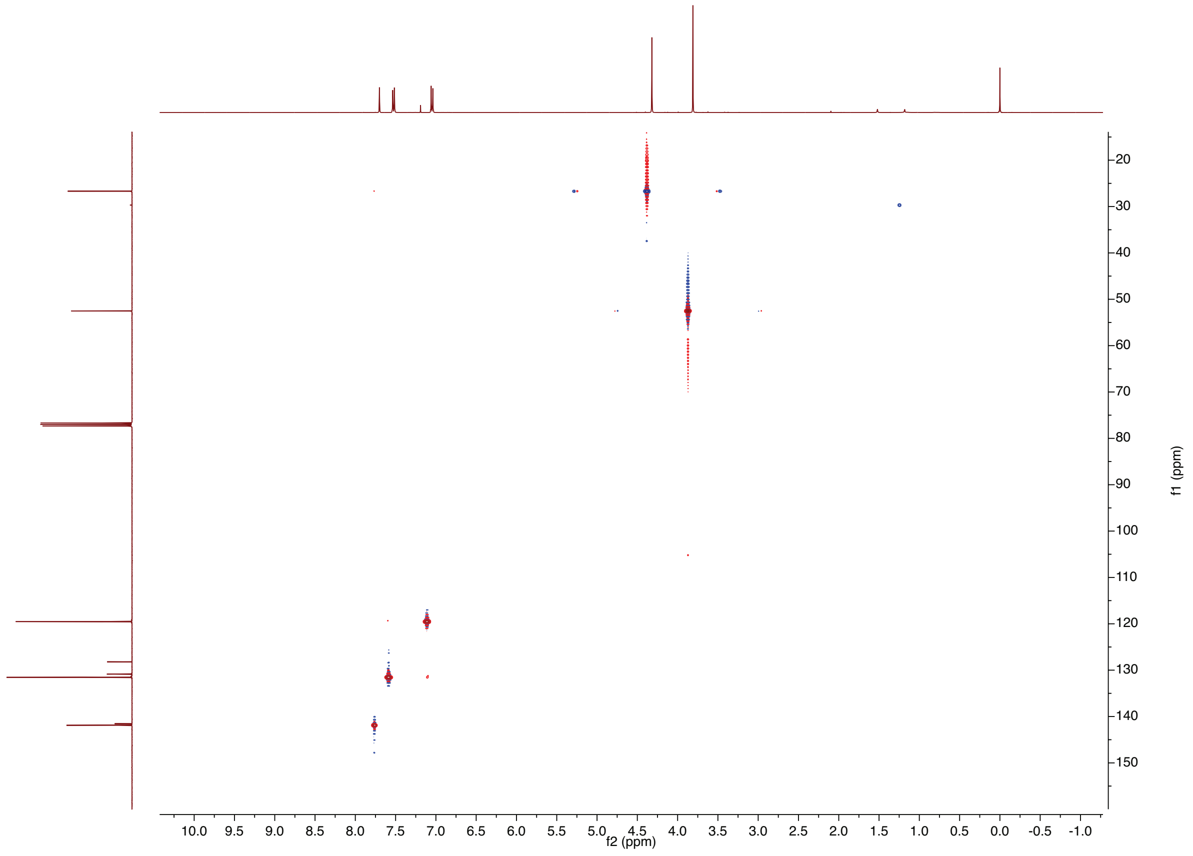

Supplementary Figure 38: gHSQC of compound **1** in $CDCl_3$.

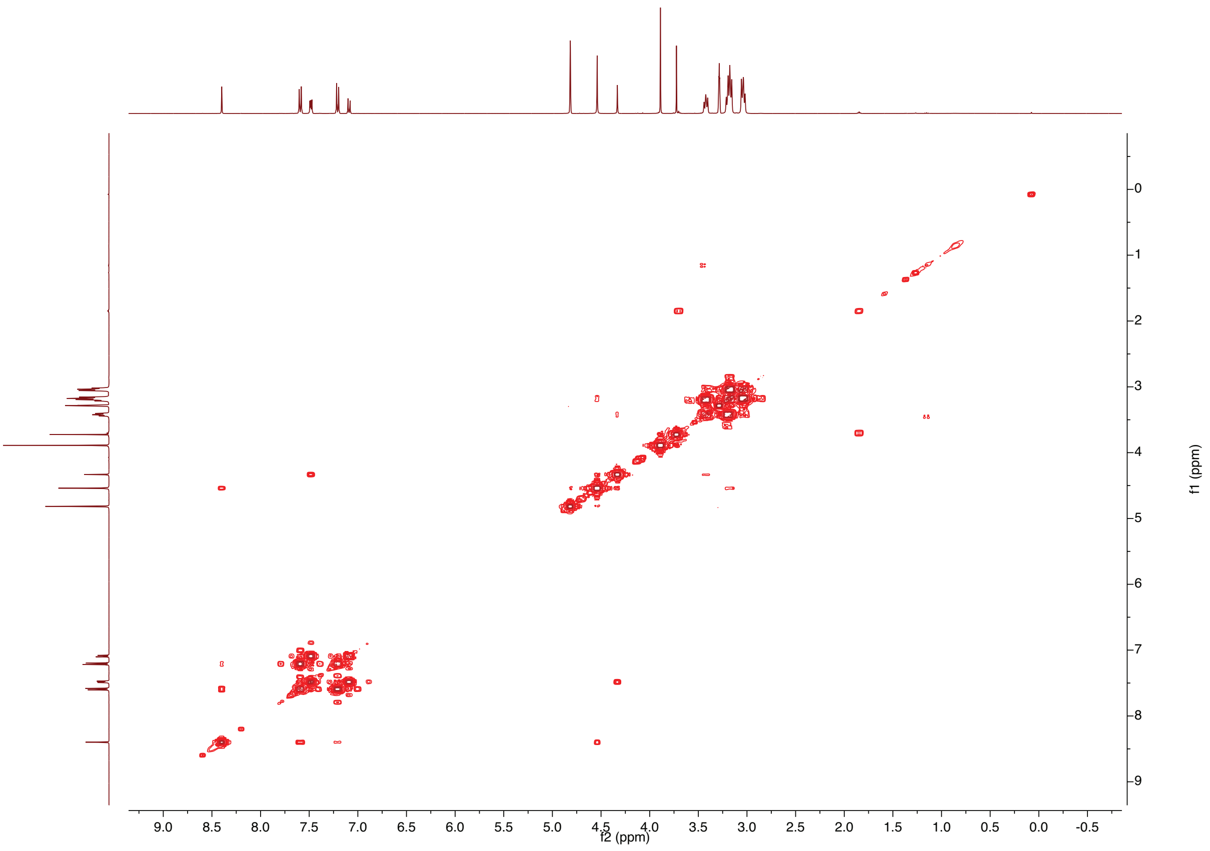

Supplementary Figure 39: gCOSY of compound **13** in MeOD.

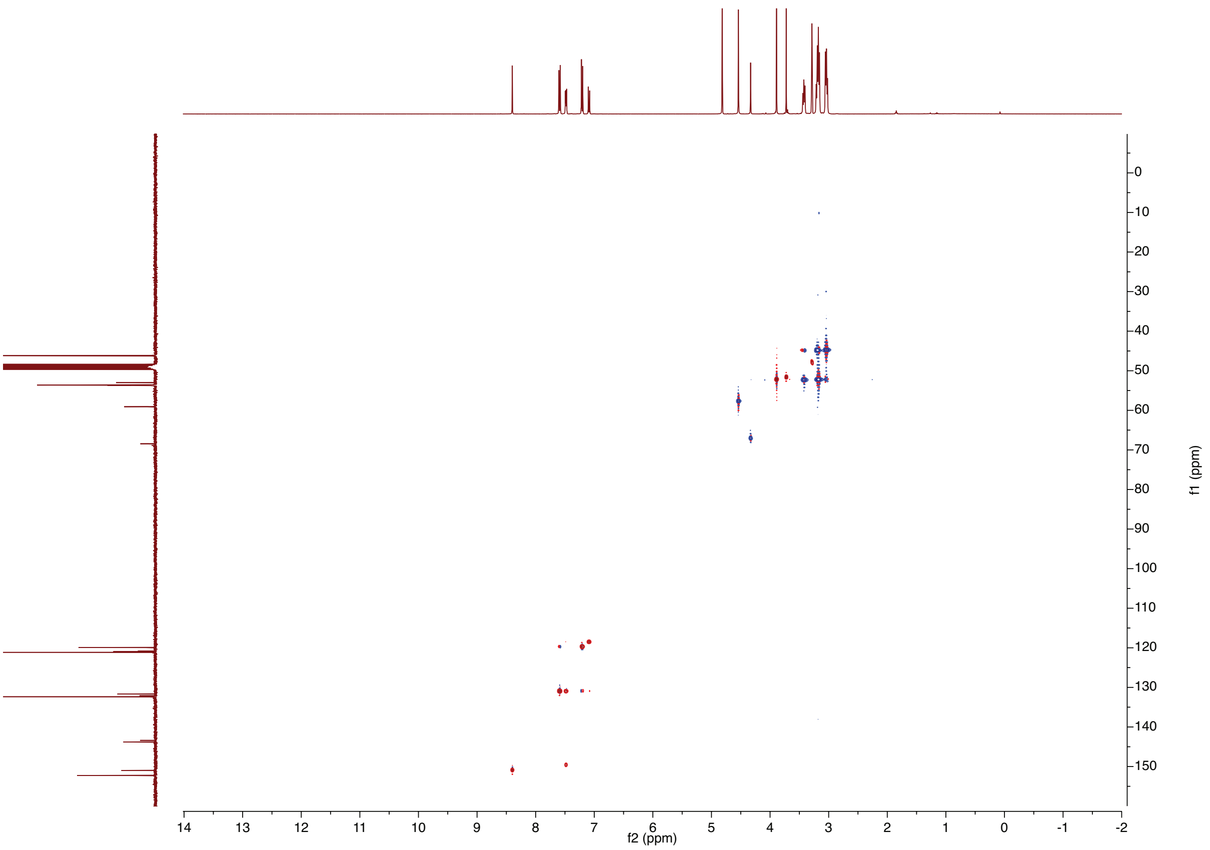

Supplementary Figure 40: gHSQC of compound **13** in MeOD.

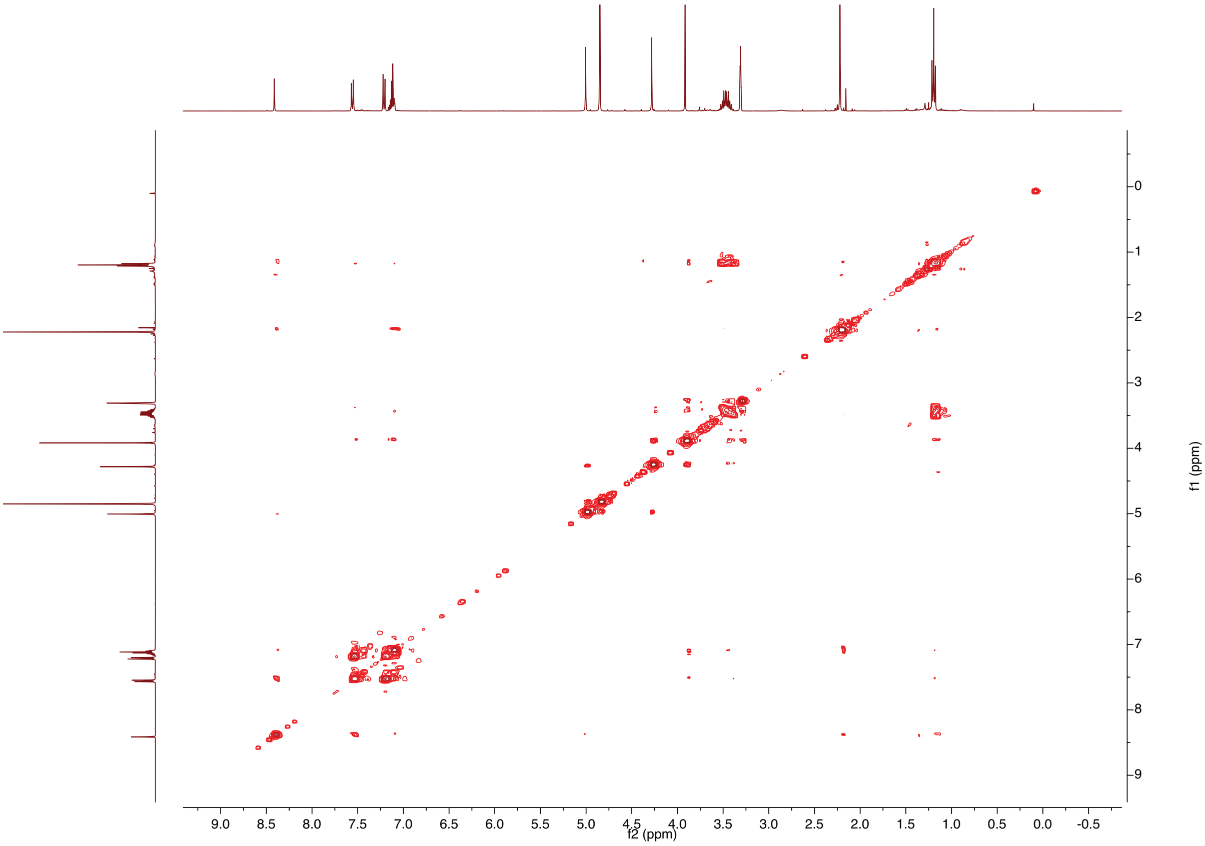

Supplementary Figure 41: gCOSY of compound **8** in MeOD.

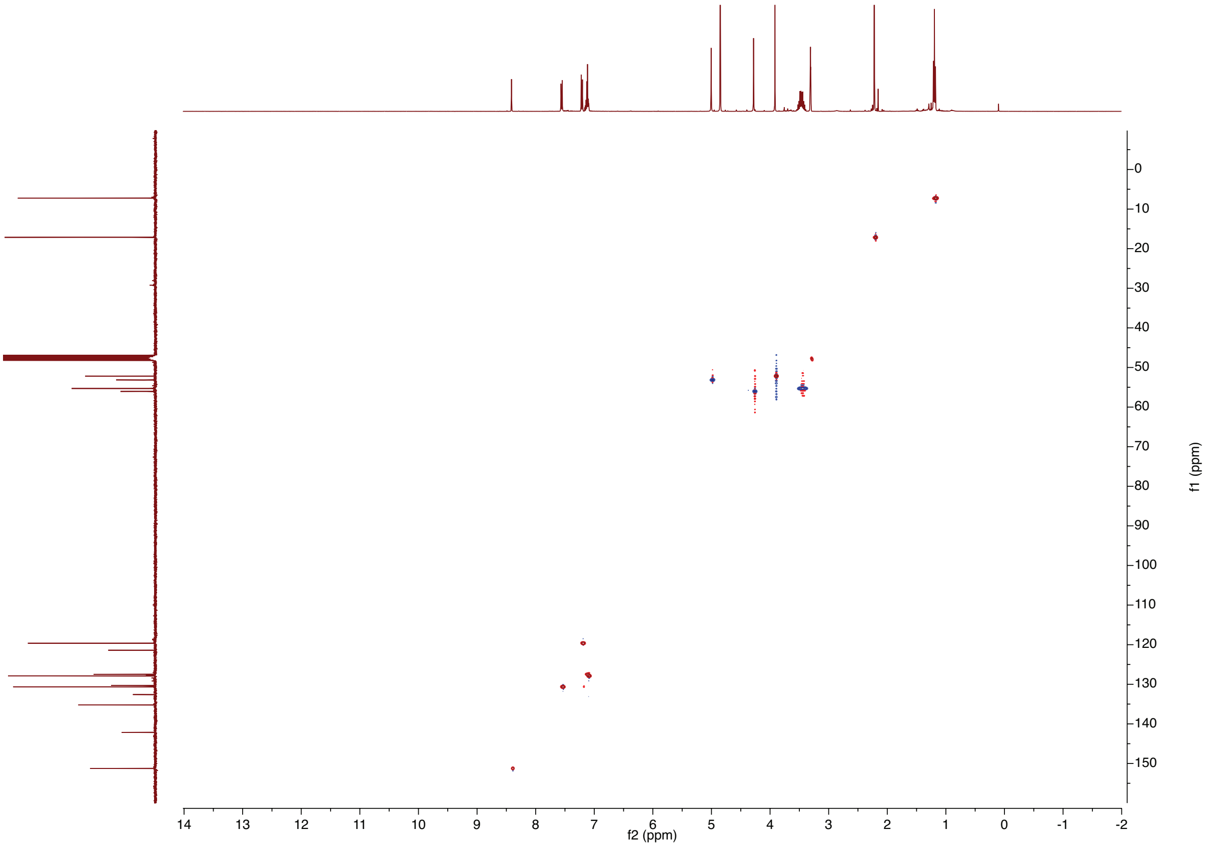

Supplementary Figure 42: gHSQC of compound **8** in MeOD.

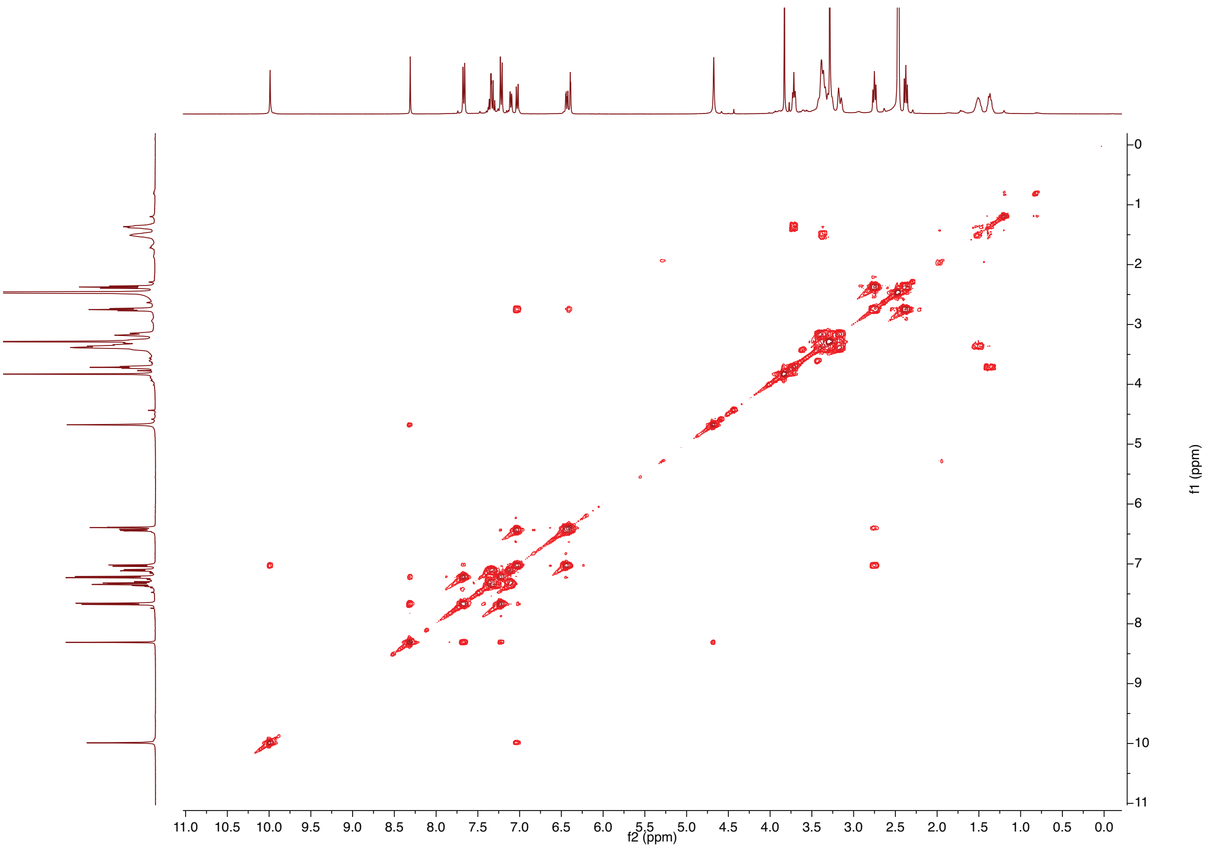

Supplementary Figure 43: gCOSY of compound **11** in d_6-DMSO.

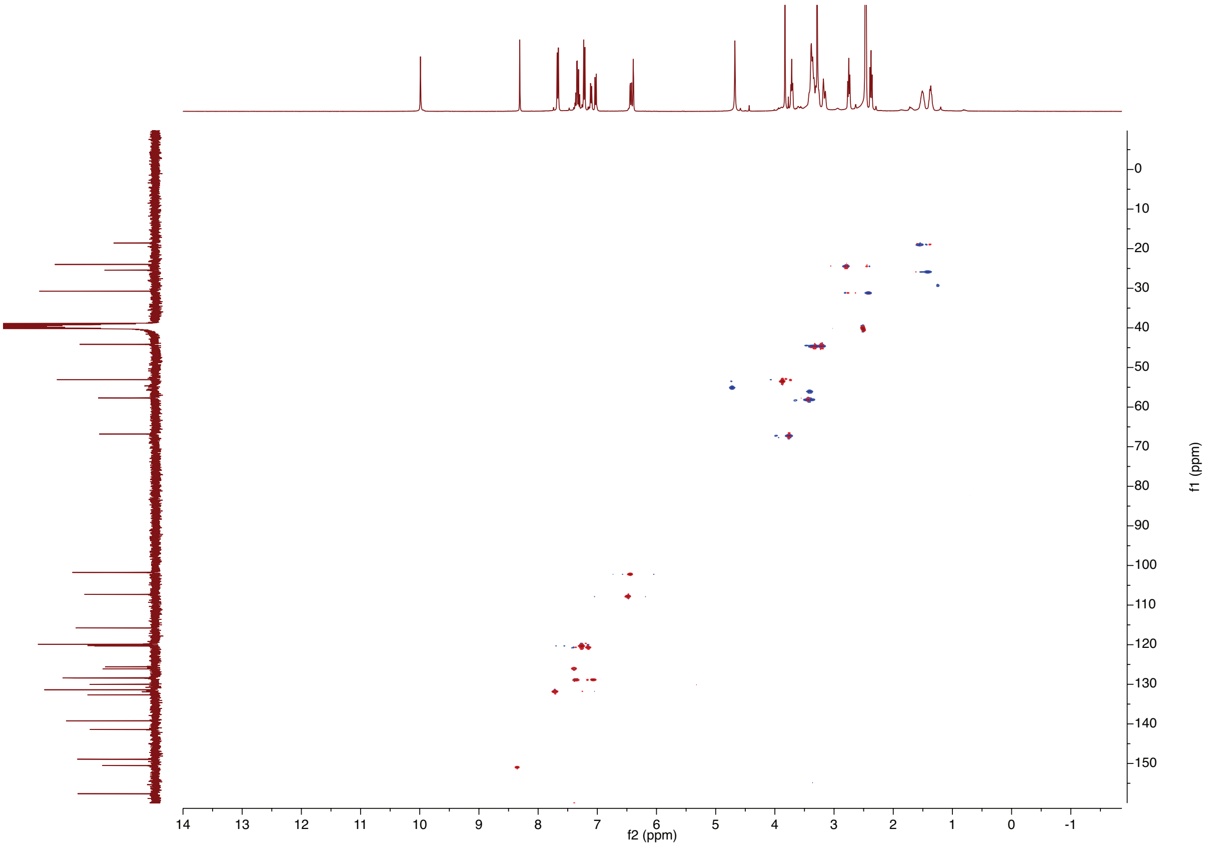

Supplementary Figure 44: gHSQC of compound **11** in d_6-DMSO.

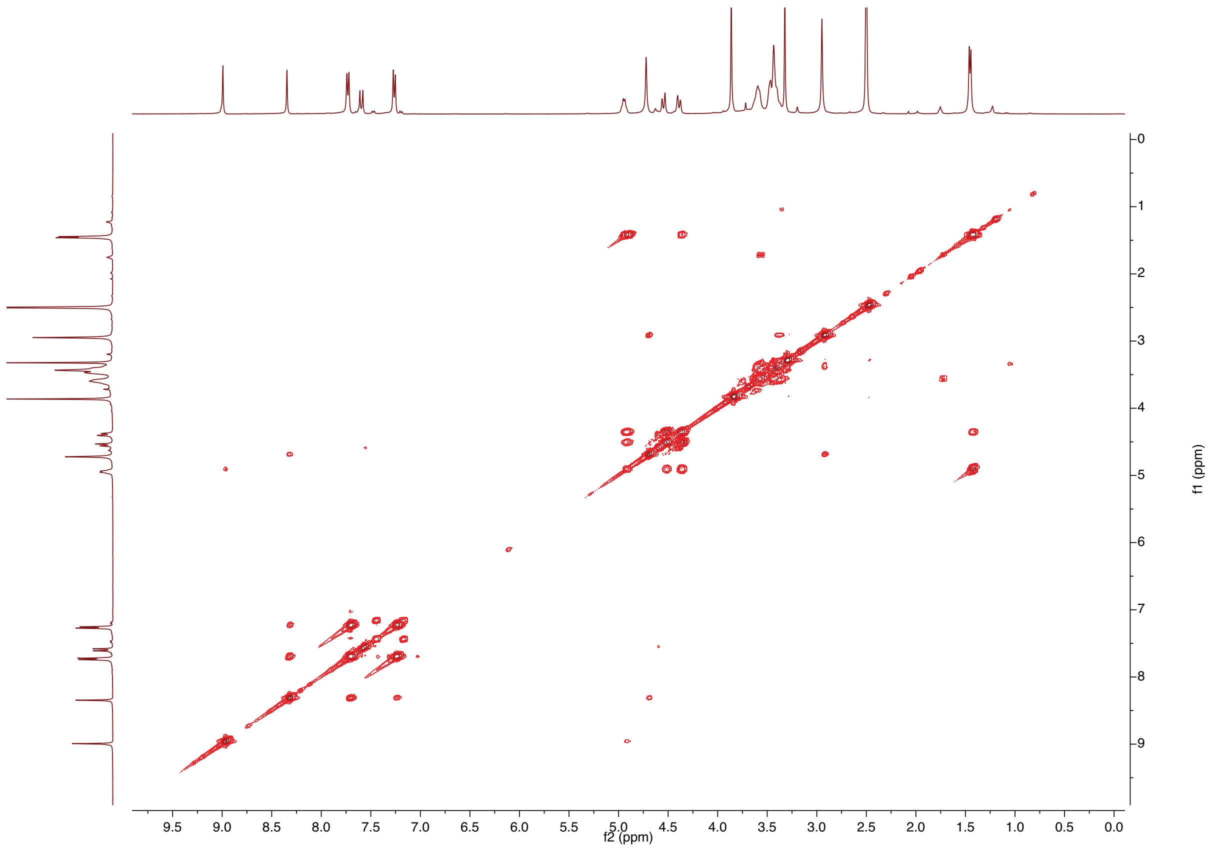

Supplementary Figure 45: gCOSY of compound **9** in d$_6$-DMSO.

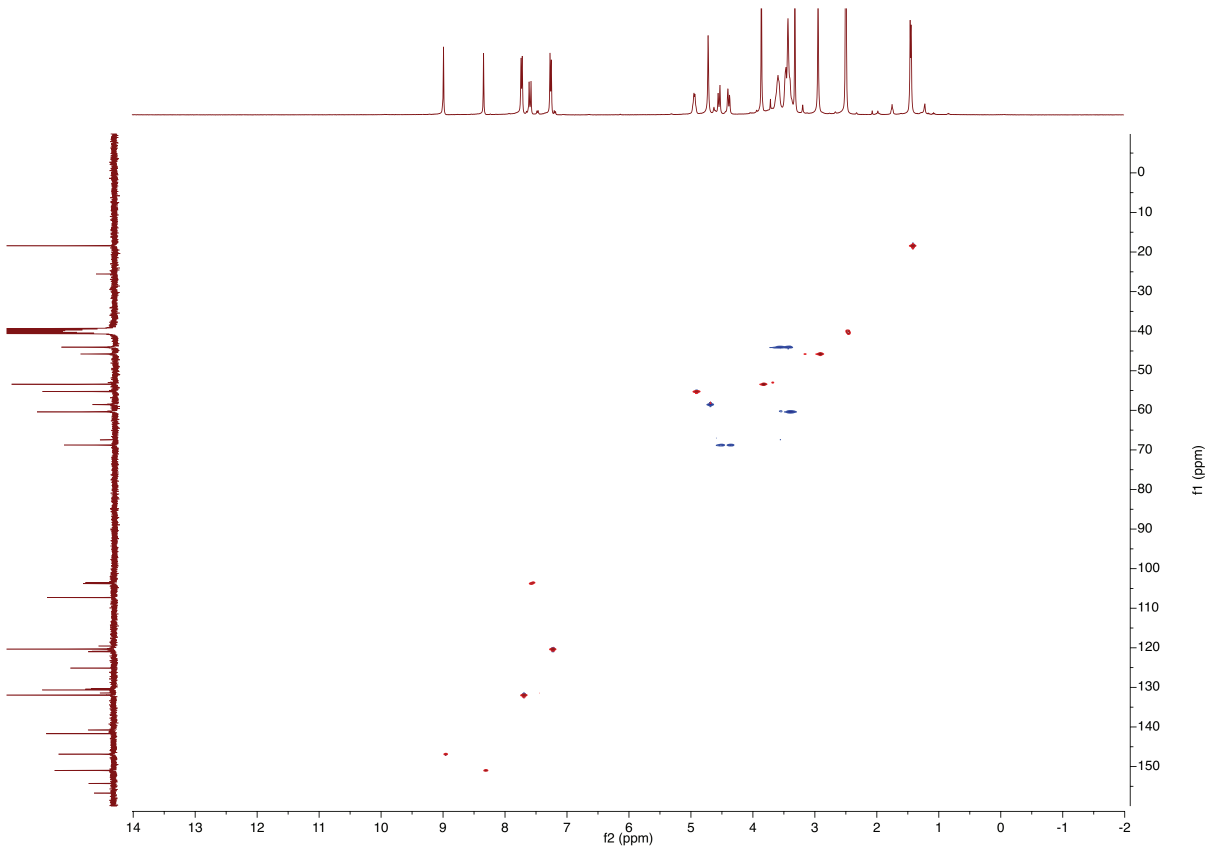

Supplementary Figure 46: gHSQC of compound **9** in d$_6$-DMSO.

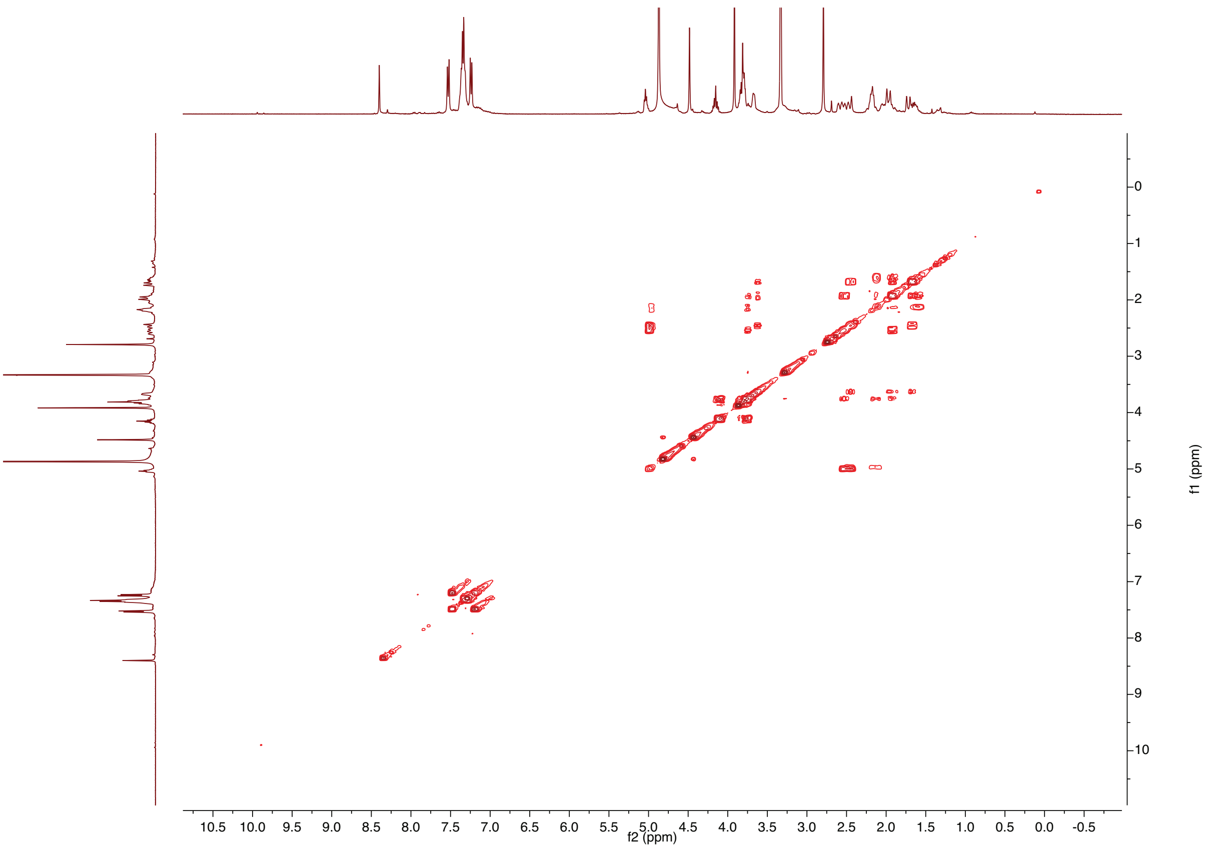

Supplementary Figure 47: gCOSY of compound **10** in MeOD.

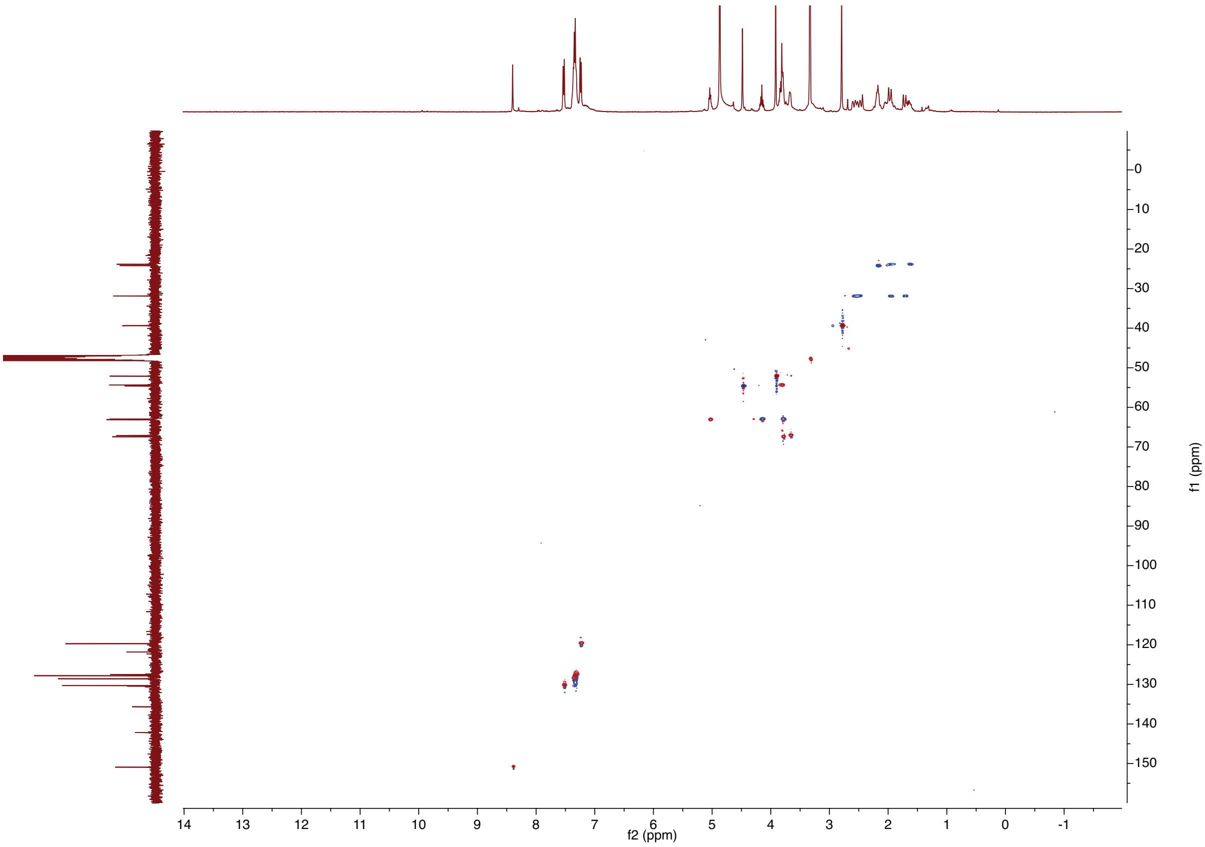

Supplementary Figure 48: gHSQC of compound **10** in MeOD.

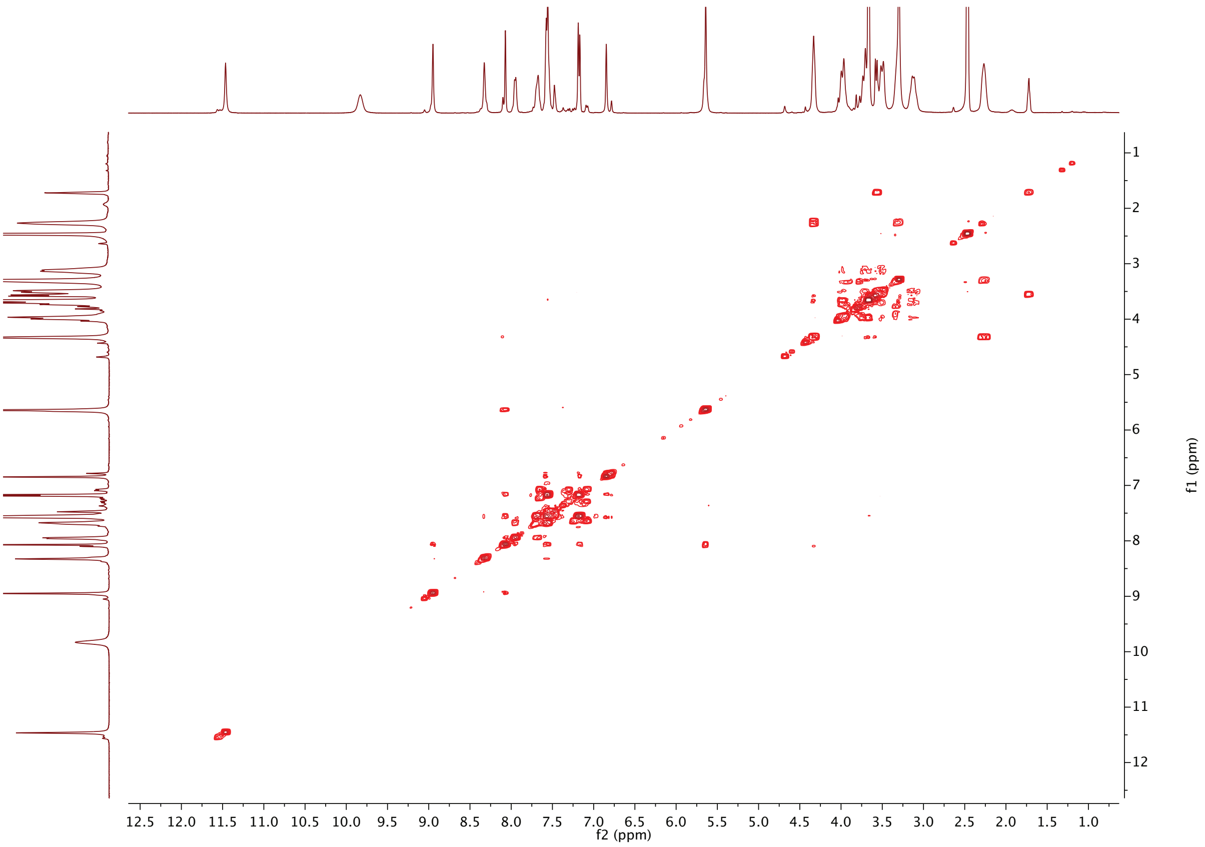

Supplementary Figure 49: gCOSY of compound **12** in d_6-DMSO.

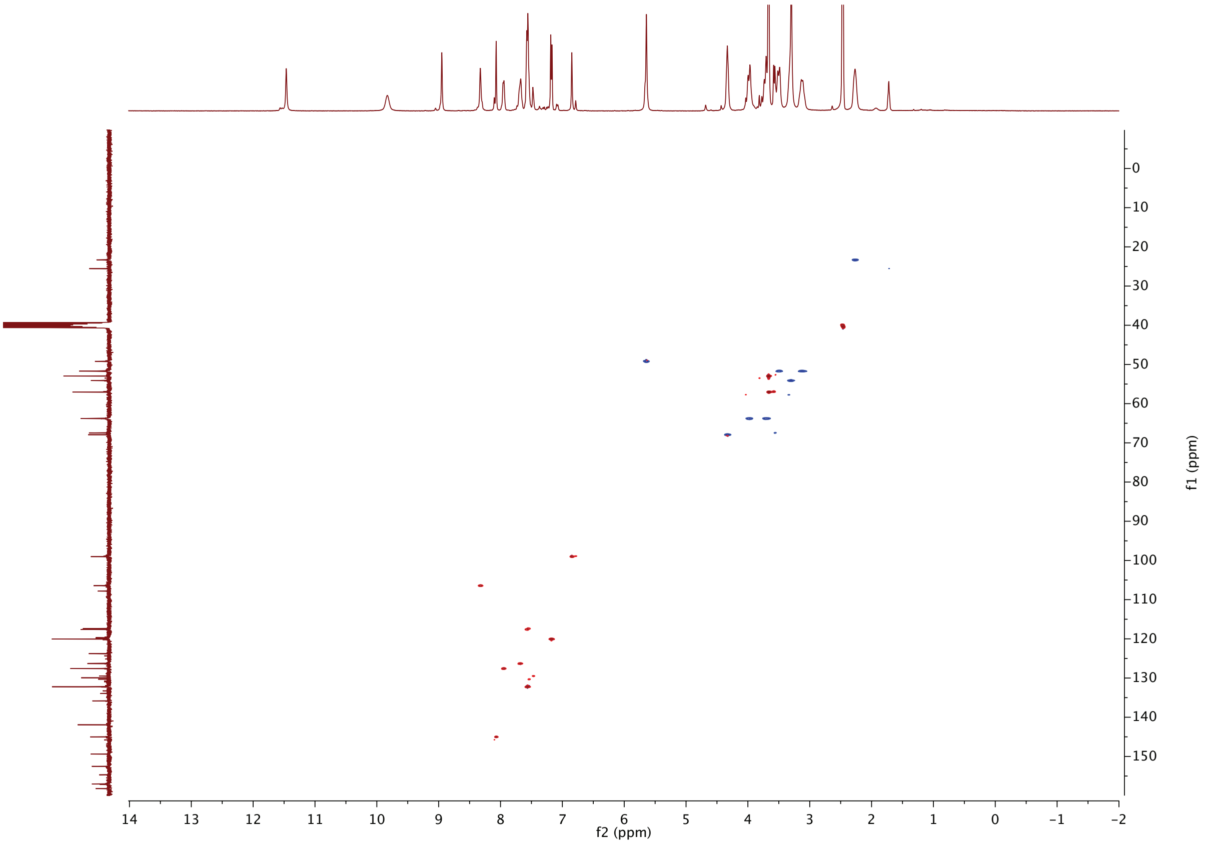

Supplementary Figure 50: gHSQC of compound **12** in d_6-DMSO.

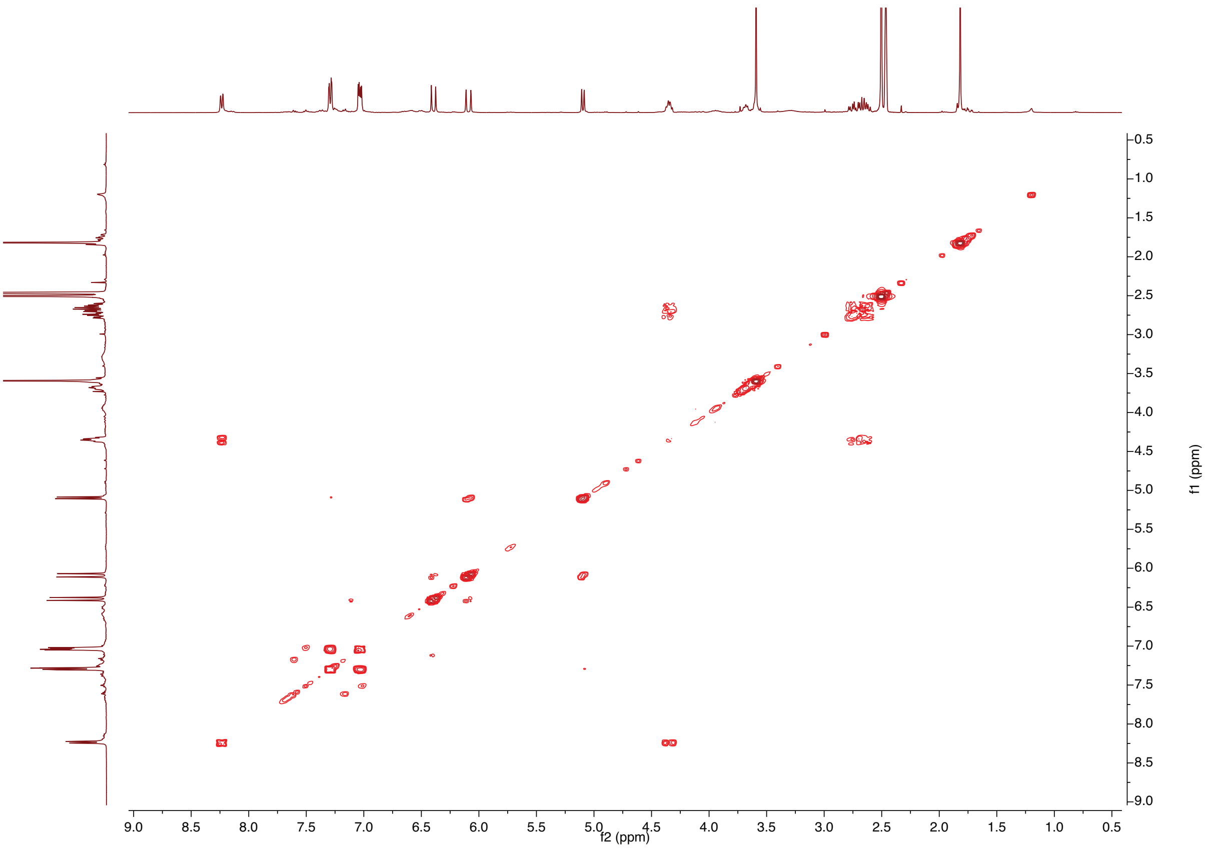

Supplementary Figure 51: gCOSY of compound **19** in d_6-DMSO.

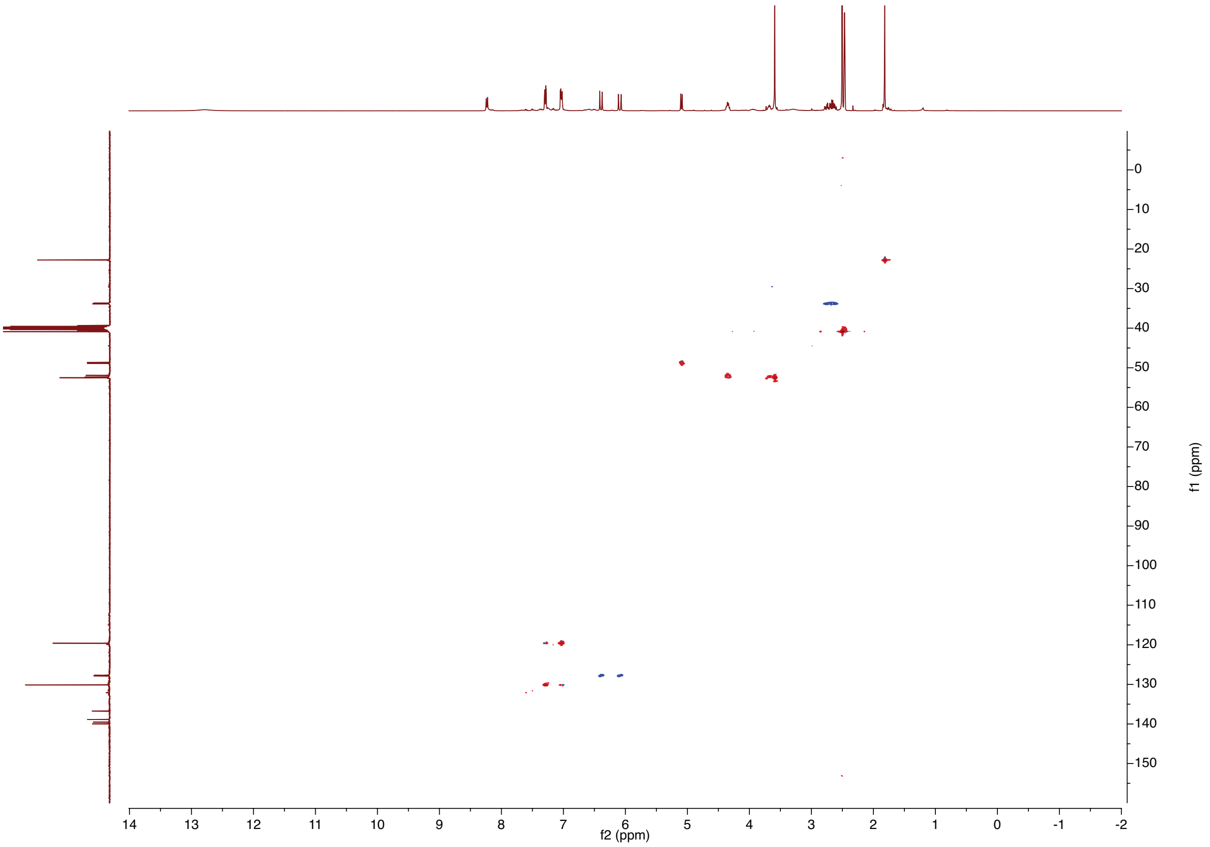

Supplementary Figure 52: gHSQC of compound **19** in d_6-DMSO.

S9.0 LC-MS data

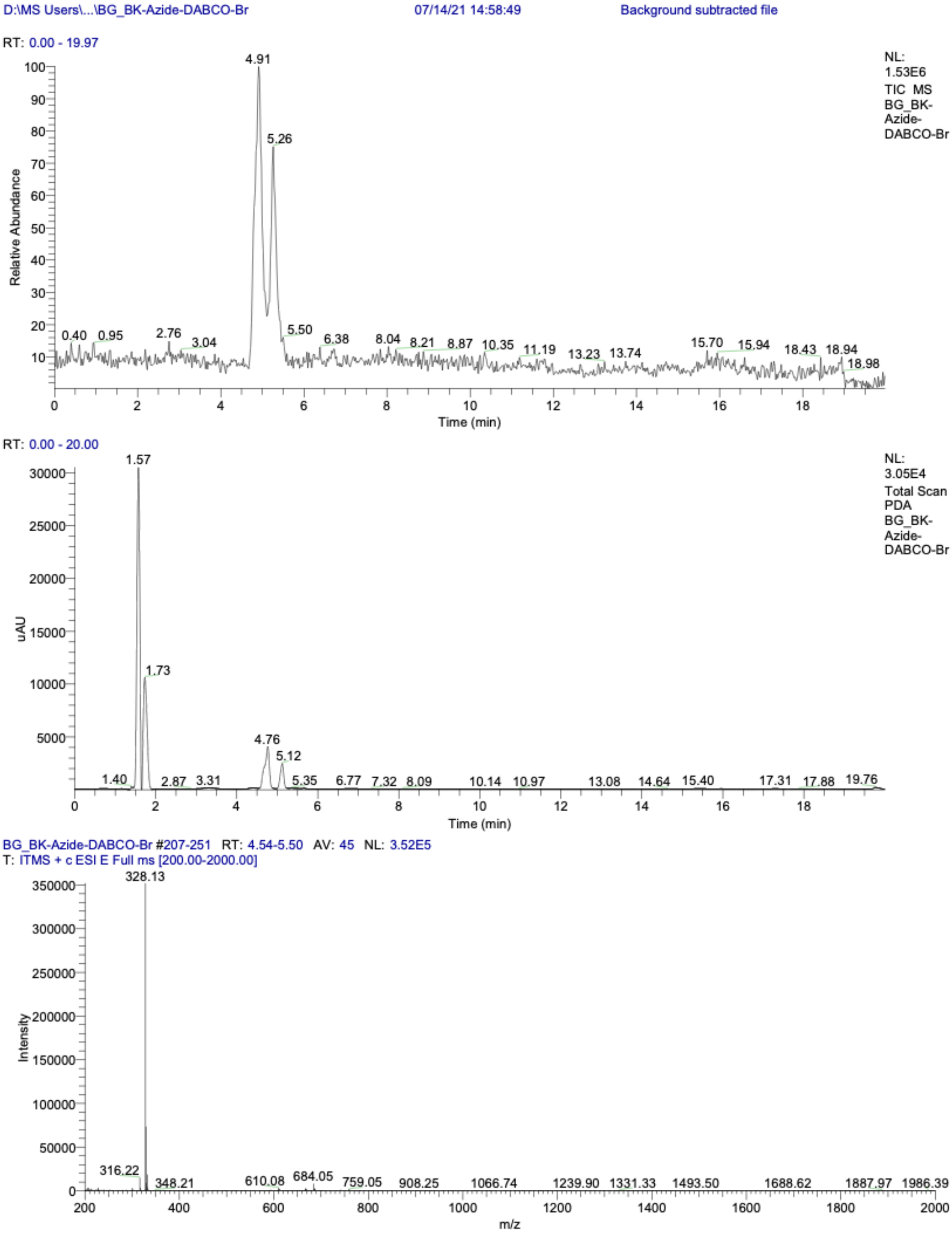

Supplementary Figure 53: LCMS-data for compound **13**.

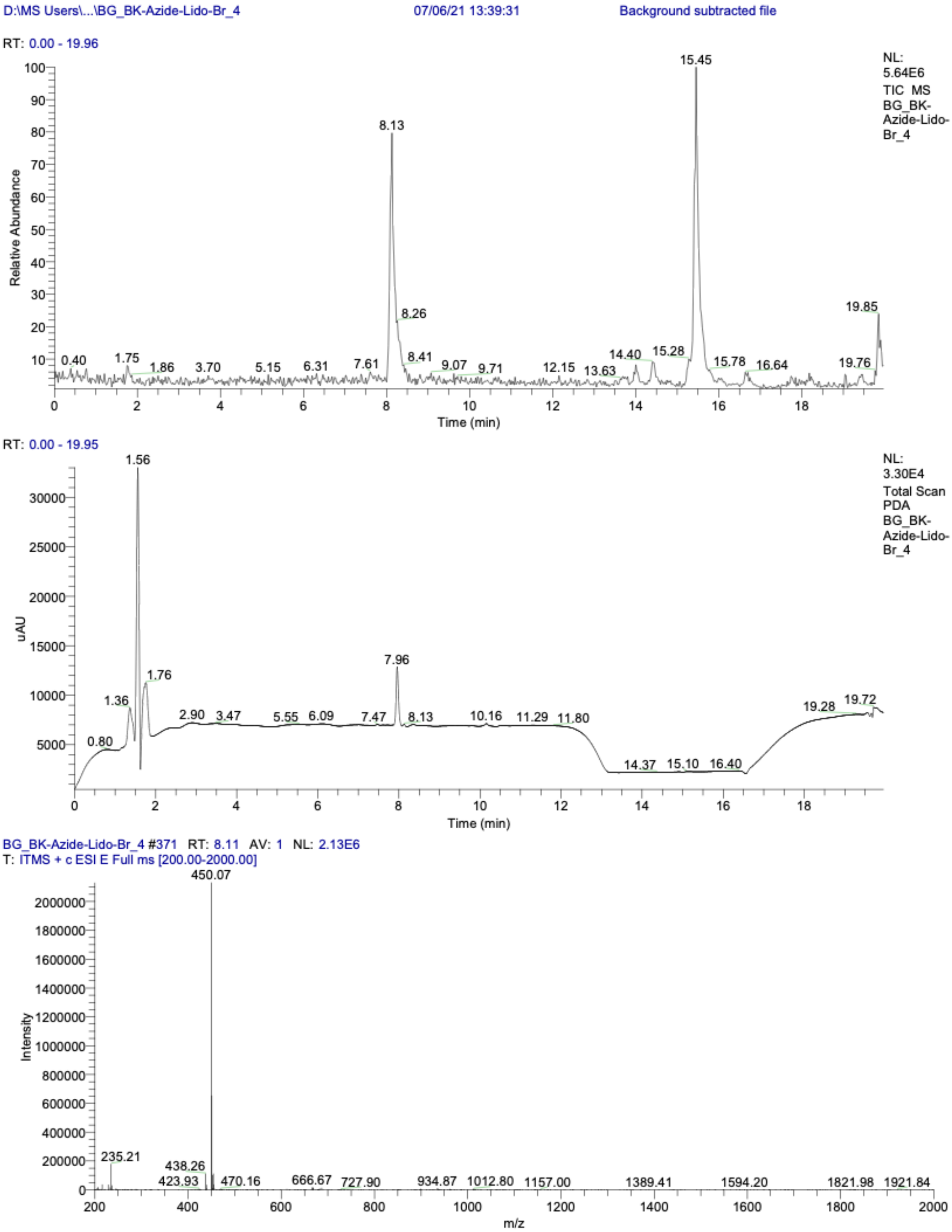

Supplementary Figure 54: LCMS-data for compound **8**.

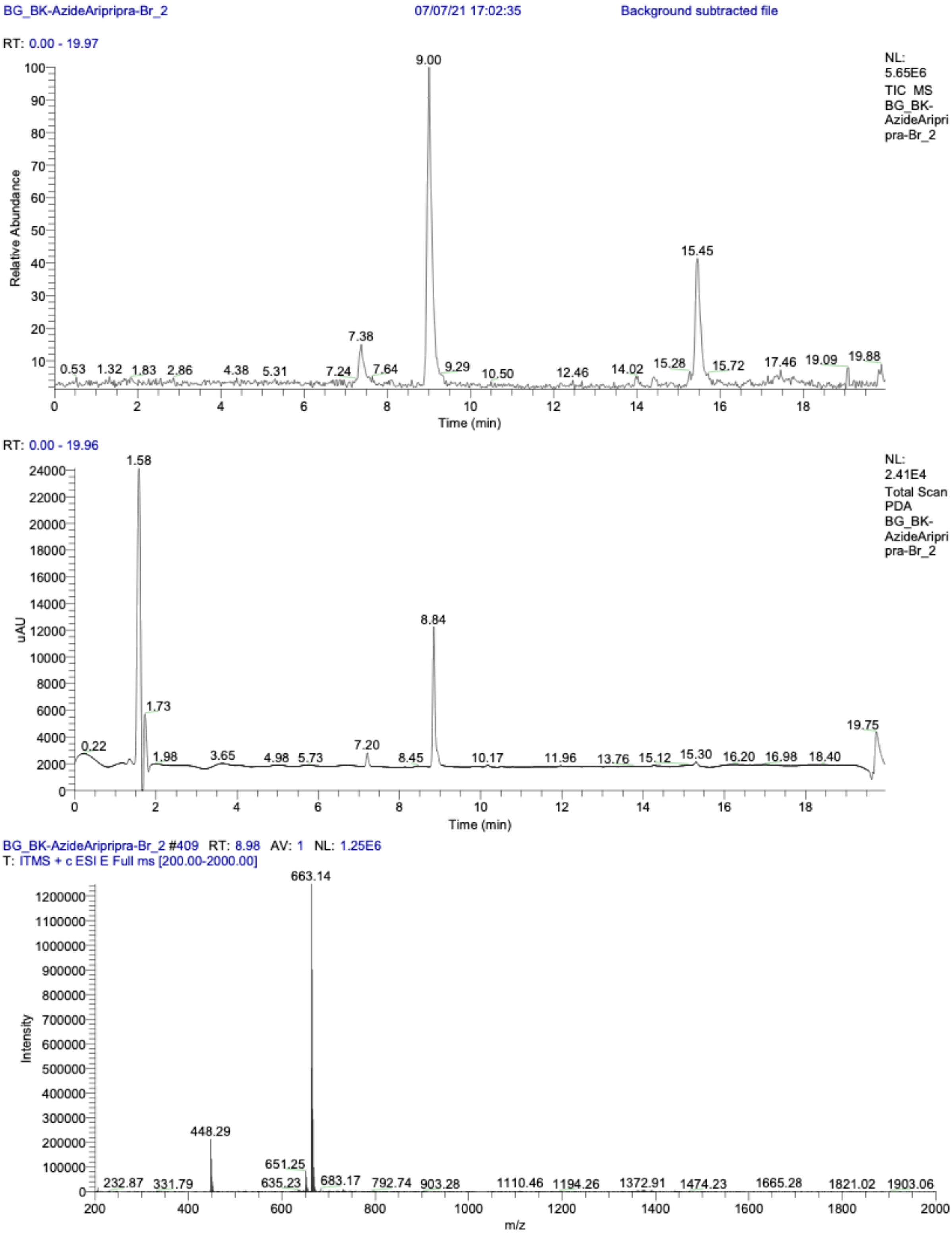

Supplementary Figure 55: LCMS-data for compound **11**.

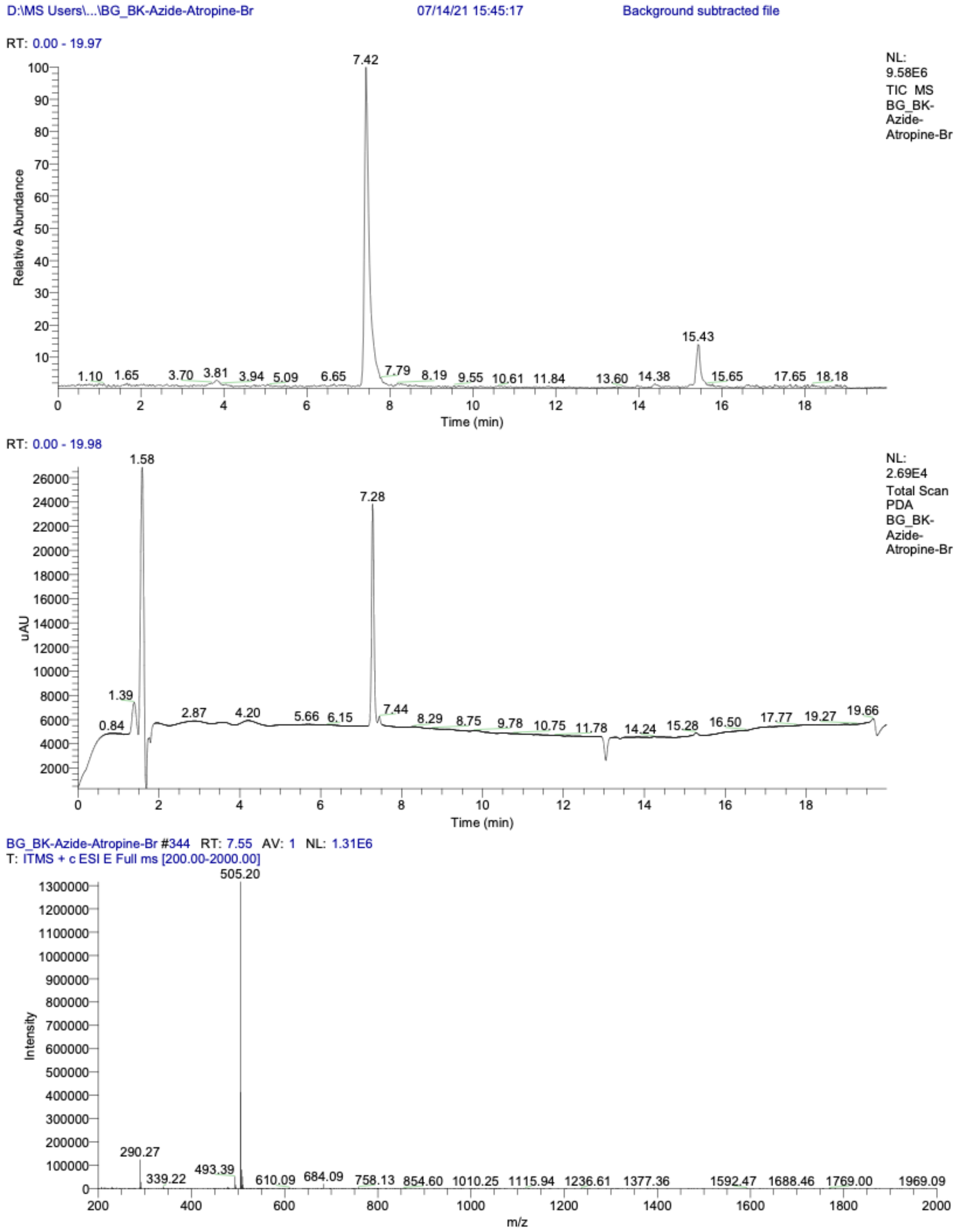

Supplementary Figure 56: LCMS-data for compound **9.**

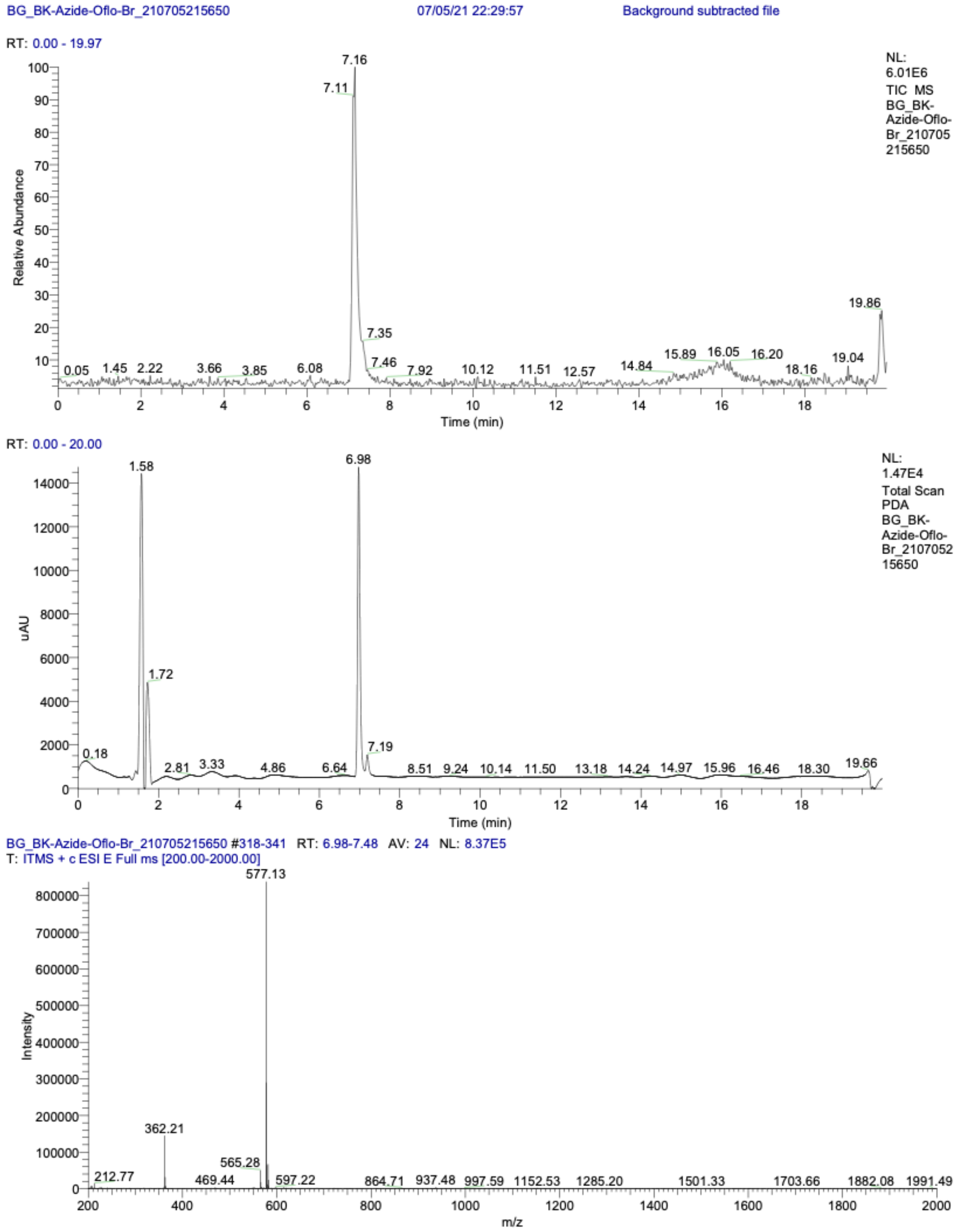

Supplementary Figure 57: LCMS-data for compound **10.**

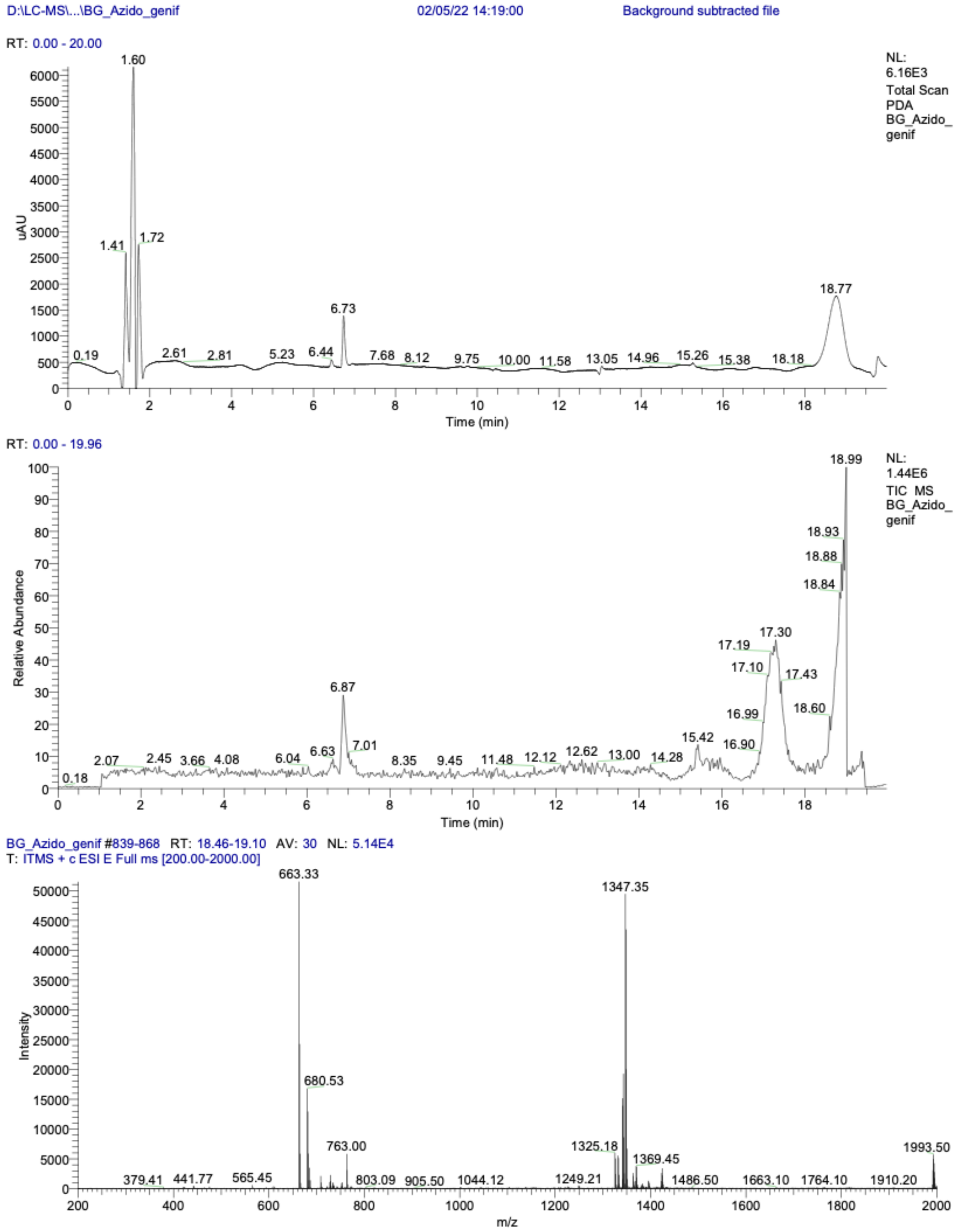

Supplementary Figure 58: LCMS-data for compound **12.**

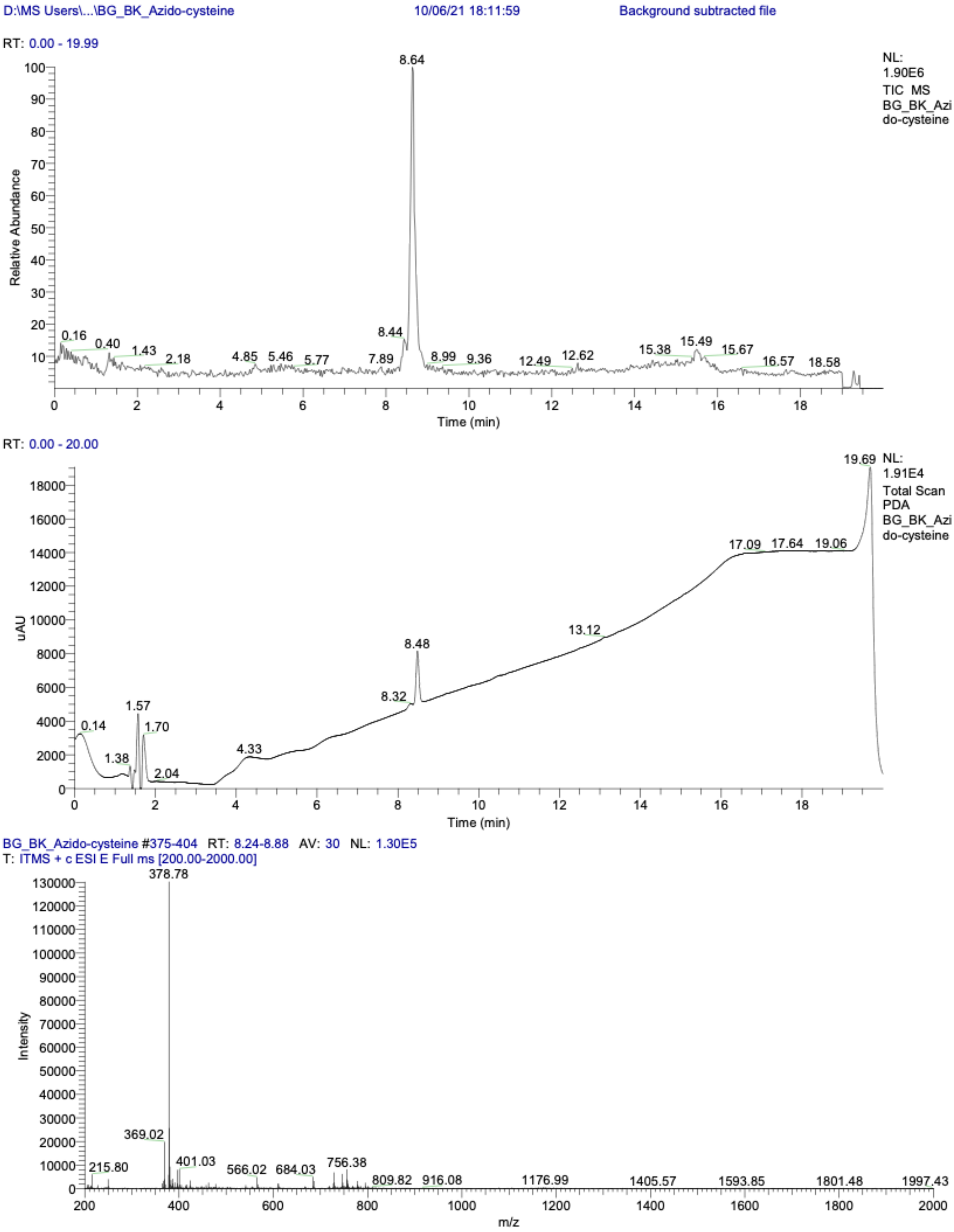

Supplementary Figure 59: LCMS-data for compound **19**.
